# Quorum sensing in *Saccharomyces cerevisiae* brewing strains: effects of 2-phenylethanol on proteomic, lipidomic, and metabolomic profile

**DOI:** 10.1093/femsyr/foaf036

**Published:** 2025-07-07

**Authors:** Scott J Britton, Jonas Niemetz, Mirjam Haensel, Jane S White, Dawn L Maskell, Florian Weiland

**Affiliations:** International Centre for Brewing and Distilling, Institute of Biological Chemistry, Biophysics, and Bioengineering, School of Engineering and Physical Sciences, Heriot-Watt University, Edinburgh EH14 4AS, Scotland; Center for Biotechnology Education (CBE), Advanced Academic Programs, Krieger School of Arts & Sciences, Johns Hopkins University, Baltimore, MD 21218, United States; Research & Development, Brouwerij Duvel Moortgat, 2870 Puurs-Sint-Amands, Belgium; Department of Bioengineering Sciences, Weihenstephan-Triesdorf University of Applied Sciences, 85354 Freising, Germany; Department of Bioengineering Sciences, Weihenstephan-Triesdorf University of Applied Sciences, 85354 Freising, Germany; International Centre for Brewing and Distilling, Institute of Biological Chemistry, Biophysics, and Bioengineering, School of Engineering and Physical Sciences, Heriot-Watt University, Edinburgh EH14 4AS, Scotland; International Centre for Brewing and Distilling, Institute of Biological Chemistry, Biophysics, and Bioengineering, School of Engineering and Physical Sciences, Heriot-Watt University, Edinburgh EH14 4AS, Scotland; Department M^2^S, Centre for Food and Microbial Technology (CLMT), Laboratory of Enzyme, Fermentation and Brewing Technology, KU Leuven – Gent, 9000 Gent, Belgium

**Keywords:** quorum sensing, sociomicrobiology, aromatic alcohols, omics, ale yeast, fungi

## Abstract

Quorum sensing (QS) is a known mechanism by which microbial populations adjust gene expression and coordinate community-wide social behaviors based on the proximate population density. This regulatory system has garnered significant interest in both scientific research and the food industry. However, a central question remains whether industrial strains of *Saccharomyces cerevisiae*, the yeast species predominantly utilized in brewing, employ quorum signalling mechanisms similar to those observed in laboratory strains and other fungi. Despite the potential relevance of microbial social behavior regulators to brewing practices, studies examining QS in *Saccharomyces* spp. are limited. In this investigation, three industrial brewing strains of *S. cerevisiae* were cultivated on SLAD (nitrogen-restrictive) and SHAD (nitrogen-sufficient) agar media supplemented with 200 μM of the aromatic alcohol 2-phenylethanol (2-PE) over 72 h at 24°C. Subsequent analyses of the harvested biomass included proteomic, lipidomic, and metabolomic assessments. Results indicated that two of the industrial strains showed minimal differences in their profiles upon exposure to 2-PE, while the third strain exhibited significant differences. These findings imply that the impact of the QS molecule 2-PE on the proteome, lipidome, and metabolome of industrial *S. cerevisiae* may be strain-specific rather than universally applicable to the species.

## Teaser

The findings indicate that the effect of the quorum-sensing molecule 2-phenylethanol on the proteomic, lipidomic, and metabolomic profiles of industrial *Saccharomyces cerevisiae* brewing strains is strain-specific, with some strains exhibiting significant biochemical changes while others remain largely unaffected.

## Introduction

Quorum sensing (QS) is a population density-dependent mechanism of intercellular communication, in which microorganisms utilize small diffusible hormone-like signalling molecules to synchronize community-wide gene expression and cooperative social behaviors (Hogan 2006a, b, Waters and Bassler 2005, West et al. 2006, Albuquerque and Casadevall 2012, Darch et al. 2012, Albuquerque et al. 2014, Özkaya et al. 2017, Schuster et al. 2017). This molecular regulatory mechanism involves the synthesis, release, and detection of signalling molecules that increase proportionally in concentration with cell replication. Once a critical concentration of the signalling molecule is reached, a coordinated physiological reaction is elicited, enabling microbial communities in close proximity to regulate gene expression and engage synchronously in a coordinated social response (Waters and Bassler 2005, Albuquerque and Casadevall 2012, Özkaya et al. 2017, Tian et al. 2021).

QS is widely recognized to enhance the benefits of cooperative social behaviors and ensures that these actions are most advantageous when the density of cooperators is elevated, leading to more effective social behaviors as compared to lower density environments (Bassler 2002, Henke and Bassler 2004, West et al. 2006, Albuquerque and Casadevall 2012, Darch et al. 2012, Schuster et al. 2017, Charlesworth et al. 2020, Thompson et al. 2023). The nature by which these social behaviors influence microbial fitness are varied, and encompass biomaterials for constructing protective str+uctures, such as exopolysaccharides that facilitate biofilm formation; toxins designed to eliminate competitors; enzymes that aid in the digestion of substrates into nutrients; biosurfactants that enhance collective motility; proteins that neutralize environmental toxins; and molecules dedicated to nutrient scavenging, among others (Crespi 2001, Velicer 2003, Parsek and Greenberg 2005, Schuster et al. 2017).

The idea of cell-density-dependent QS regulation of fungal social behaviors was first identified by Lingappa et al. (1968) while studying the filamentation response to 2-phenylethanol (2-PE) and tryptophol in the human fungal pathogen *Candida albicans*. Since then, a wide range of structurally diverse fungal QS molecules have been identified, which regulate numerous social characteristics across evolutionarily diverse fungal species (Chen and Fink 2006, Lee et al. 2007, Horowitz Brown et al. 2008, Raina et al. 2010, Berrocal et al. 2012, 2014, Williams et al. 2012, Albuquerque et al. 2013, Homer et al. 2016, Vitale et al. 2019, Britton et al. 2024). In one example, *Saccharomyces cerevisiae* strain Σ1278b was shown to undergo a reversible yeast-to-filamentous morphogenic response upon exposure to the active QS molecules 2-PE and tryptophol under nitrogen-restrictive conditions, which subsequently relies on the cyclic adenosine monophosphate (AMP)-dependent protein kinase A (PKA) subunit Tpk2 to promote Flo8-mediated transcriptional activation of Flo11, a glycosylphosphatidylinositol (GPI)-anchored cell surface glycoprotein involved in pseudohyphal (diploid) and haploid invasive growth, flocculation, and biofilm formation (Guo et al. 2000, Bayly et al. 2005, Chen and Fink 2006, Britton et al. 2021, 2022, Bouyx et al. 2021). This social behavior, a quorum-controlled phenotype, is thought to be a foraging strategy in response to nutrient stress, and is characterized by elongated cell morphology, unipolar budding, and incomplete scission of mother–daughter cells (Gimeno et al. 1992).

However, two more recent investigations have demonstrated that the filamentous growth response is a generalized, highly variable strain-specific phenotype in both natural populations, clinical isolates, fermentation reactions, and domesticated brewing strains (Lenhart et al. 2019, Britton et al. 2023). In the latter study, almost 38% of domesticated industrial brewing strains examined failed to exhibit any filamentation upon exposure to nitrogen-restrictive media containing 0, 100, and 200 μM concentrations of 2-PE. However, despite the recently documented effects of 2-PE on quorum-controlled filamentous social behavior, no comprehensive investigation has yet been undertaken to examine how this QS molecule influences the proteome, lipidome, and metabolome of domesticated brewing yeasts. Beer fermentation and production take place in a diffusion-limited, densely populated environment, where the physiology and metabolic activity of yeast significantly contributes to the formation of ethanol and various other flavour-active compounds critical to the beer’s flavour profile and consumer acceptability (Hough and Stevens 1961, Peddie 1990, Shimizu et al. 2001, Verstrepen et al. 2003, Lodolo et al. 2008, Saerens et al. 2008, 2010, Pires et al. 2014, Wauters et al. 2021, Simbaña et al. 2022, Methner et al. 2022, Kato and Takahashi 2023, Cui et al. 2023, Ogata and Saito 2024, Maruyama et al. 2024, Murath et al. 2025). Therefore, it is reasonable to suggest that QS mechanisms could fundamentally influence the fermentation dynamics modification of gene expression and cellular activity within the yeast and, consequently, the quality of the final product.

In this investigation, three industrially relevant brewing strains were cultivated on SLAD (nitrogen-limiting) and SHAD (nitrogen-sufficient) microbiological media, either without supplementation or with 200 μM (24.43 mg/l) of the aromatic alcohol 2-PE, a concentration representative in beer and other closely related beverage alcoholic fermentation processes, for 72 h at 24°C (Ayrapaa 1961, Szlavko 1973, Li et al. 2008, Zupan et al. 2013, Avbelj et al. 2015). Afterwards, the accumulated biomass from each condition was harvested from the agar plates and underwent proteomic, lipidomic, and metabolomic analysis. Results suggest that the influence of the QS molecule 2-PE on the proteome, lipidome, and metabolome may also be a strain-specific phenomenon.

## Materials and methods

All chemicals, reagents, and materials used in this study were purchased from Merck KGaA (Darmstadt, Germany), unless otherwise noted. For clarity, any materials sourced from other suppliers are explicitly mentioned in the respective sections of the methodology.

### Yeast strains

The yeast strains YMD4537 and YMD4544 were sourced from Brouwerij Duvel Moortgat (Puurs-Sint-Amands, Belgium). YMD4529 was sourced from White Labs, LLC (San Diego, CA, USA, catalogue number WLP006). Taxonomic identities were confirmed as top-fermenting ale yeast (*S. cerevisiae*) using real-time PCR (GEN-IAL, Troisdorf, Germany, catalogue number Q072).

### Solid and liquid media

Yeast extract peptone sucrose (YPS) agar plates [10% (w/v) sucrose (Colruyt Group, Halle, Belgium), 1% (w/v) yeast extract (Thermo Fisher, Waltham, MA, USA, catalogue number LP0021B), 0.5% (w/v) peptone (Thermo Fisher, catalogue number LP0034B), 9 mM (NH_4_)_2_SO_4_ (catalogue number A5132), 8 mM NaCl (Colruyt Group), 7 mM KH_2_PO_4_ × 2 H_2_O (catalogue number 104873), 3 mM MgCl_2_ × 7 H_2_O (Acros Organics BV, Geel, Belgium, catalogue number 197530010), 700 μM CaCl_2_ × 2 H_2_O (Acros Organics, catalogue number 207780010), 22 μM ZnCl_2_ (Acros Organics, catalogue number AC198945000), 2% (w/v) agar (VWR international, Radnor, PA, USA)], SLAD agar plates [2% (w/v) glucose (catalogue number 158968), 0.17% (w/v) yeast nitrogen base (without amino acids, without ammonium sulphate, Thermo Fisher, catalogue number 11326759), 50 μM (NH_4_)_2_SO_4_, 2% (w/v) agar (VWR international)], SHAD agar plates [2% (w/v) glucose, 0.17% (w/v) yeast nitrogen base (without amino acids, without ammonium sulphate, Thermo Fisher), 32 mM (NH_4_)_2_SO_4_, 2% (w/v) agar (VWR international)], and Yeast extract peptone dextrose (YPD) liquid medium [2% (w/v) glucose, 2% (w/v) peptone (Thermo Fisher), 1% (w/v) yeast extract (VWR international)] were prepared as described in Britton et al. (2023).

### Yeast cell culture

Yeasts strains were cultivated on YPS agar plates and incubated for 72 h at 24°C. Afterwards five individual single colonies per yeast strain were selected and precultured in 100 ml liquid YPD medium each for 24 h at 24°C. Yeast cells from each strain and replicate were then washed twice in phosphate-buffereds aline (PBS) [137 mM NaCl, 100 mM Na_2_HPO_4_ (catalogue number S0876), 17.6 mM KH_2_PO_4_ × 2 H_2_O, 2.7 mM KCl (catalogue number 1.04936.0250)] and 1 × 10^5^ cells were plated on either SLAD (*n* = 10) or SHAD (*n* = 6) agar plates, or their 200 μM 2-PE (catalogue number 77861) supplemented counterparts. The plates were then incubated for 72 h at 24°C. Yeast cells were scraped off the surface of the agar plates (4 biological replicates for each of the 4 growth conditions = 16 samples per yeast strain), washed in PBS and split into two aliquots for proteomics and lipidomics/metabolomic analysis. Resulting cell pellets were snap frozen in liquid nitrogen and stored at −80°C until further usage.

### Sample preparation for proteome analysis

The frozen yeast cell pellet was ground in liquid nitrogen using a mortar and pestle. The resulting powder was then resuspended in ice-cold solution composed of 80% (v/v) acetone (catalogue number 1070212511), and 20% (v/v) trichloroacetic acid (catalogue number T0699-100ML). The mixture was then incubated overnight at 4°C to facilitate protein precipitation. Samples were centrifuged at −10°C, 7000 × *g* for 20 min (Micro-Centrifuge CD-3124, Phoenix Instruments, Germany). The supernatant was discarded, ice-cold 100% (v/v) acetone was added to the protein pellet and the samples were incubated for 30 min at −20°C, with intermittent vortexing every 10 min. Samples were centrifuged at −10°C, 7000 *× g* for 20 min and 8 M Urea (catalogue number 1.08487.0500), 50 mM NH_4_HCO_3_ (catalogue number 09830–500 G) was added to the pellet. Proteins were resuspended using a 120 W sonicator (Thermo Fisher, catalogue number 12337338), for 2 × 30 s with an amplitude of 50% and a pulse duration of 1 s. Afterwards, the samples were centrifuged at 7000 × *g* and 4°C for 20 min and the supernatant was retained. Protein concentration was determined using a bicinchoninic acid (BCA) protein assay kit (Thermo Fisher, catalogue number 23225) according to the manufacturer’s protocol.

### Tryptic digest

15 μg of each sample was reduced and alkylated for 45 min in the dark using 10 mM Tris(2-carboxyethyl)phosphine-hydrochloride (catalogue number C4706) and 25 mM chloroacetamide (catalogue number C0267). Samples were then diluted 1:4 in 50 mM NH_4_HCO_3_, 300 ng trypsin (Thermo Fisher, catalogue number 90058) was added and proteolysis was conducted for 18 h at 25°C. Afterwards, the digestion was stopped by addition of trifluoroacetic acid (TFA, catalogue number 1.08262.0025) to a final concentration of 0.1% (v/v).

### Labelling of peptides using tandem mass tags

Peptides (10 μg) were desalted using home-packed C18 tips (SPE discs, 3 M, Saint Paul, MN, USA, catalogue number 66883-U), snap frozen in liquid nitrogen, freeze-dried (Alpha 2–4 LSC, Martin Christ Gefriertrocknungsanlagen GmbH, Osterode, Germany) and resuspended in 71.4 mM HEPES, pH 8.5 (catalogue number 54457–10 G). TMT16plex labels (Thermo Fisher, catalogue number A44521) were resuspended in anhydrous ACN (catalogue number 271004), added to the samples (one TMT16plex for the 16 samples of each yeast strain) and final ACN concentration in the samples was adjusted to 30% (v/v). The labelling reaction was conducted for 2 h at room temperature. The labelling reaction was stopped by the addition of hydroxylamine (catalogue number 467804) to a concentration of 0.5% (w/v) and incubation for 15 min at room temperature. Afterwards, the tandem mass tags (TMT)-labelled samples of each strain were combined, resulting in three sets of TMT16plexes, snap frozen, freeze dried, and stored at −80°C until later use.

### Liquid chromatography coupled tandem mass spectrometry

The samples were resuspended in 2% (v/v) acetonitrile (ACN), 0.1% formic acid (catalogue number 5.33002.0050), incubated in an ultrasonic bath (Digitec DT52, BANDELIN electronic GmbH & Co. KG, Berlin, Germany) for 15 min, and analysed by the proteomics core facility of the Vlaamse Institute for Biotechnology (VIB, Gent, Belgium).

Peptides were redissolved in 20 µl loading solvent A [0.1% TFA in water/ACN (98:2, v/v)], of which 15 µl was injected for liquid chromatography coupled tandem mass spectrometry (LC–MS/MS) analysis on an Ultimate 3000 RSLCnano system in-line connected to a Q Exactive HF mass spectrometer (Thermo). Trapping was performed at 20 μl/min for 2 min in loading solvent A on a 5 mm trapping column (Thermo scientific, 300 μm internal diameter, 5 μm beads). The peptides were separated on a 250 mm Aurora ultimate, 1.7 µm C18, 75 µm inner diameter (Ionopticks) kept at a constant temperature of 45°C. Peptides were eluted by a nonlinear gradient starting at 0.5% MS solvent B reaching 33% MS solvent B [0.1% FA in water/ACN (2:8, v/v)] in 135 min, 55% MS solvent B [0.1% FA in water/ACN (2:8, v/v)] in 155 min, 70% MS solvent B in 160 min followed by a 5-min wash at 70% MS solvent B and reequilibration with MS solvent A (0.1% FA in water). The mass spectrometer was operated in data-dependent mode, automatically switching between MS and MS/MS acquisition for the 12 most abundant ion peaks per MS spectrum. Full-scan MS spectra (350–1500 m/z) were acquired at a resolution of 60 000 in the Orbitrap analyser after accumulation to a target value of 3 000 000. The 12 most intense ions above a threshold value of 15 000 were isolated with a width 1.5 m/z for fragmentation at a normalized collision energy of 33% after filling the trap at a target value of 13 000 for maximum 80 ms. MS/MS spectra (with a fixed first mass of 120 m/z) were acquired at a resolution of 45 000 in the Orbitrap analyser. The polydimethylcyclosiloxane background ion at 445.120 028 Da was used for internal calibration (lock mass) and QCloud (Chiva et al. 2018, Olivella et al. 2021) has been used to control instrument longitudinal performance during the project.

### Data analysis—proteomics

The MS raw data was searched against a *S. cerevisiae* FASTA (downloaded from Uniprot on 12 December 2023, 6060 entries) to which decoy entries and contaminants were added by FragPipe (version 22.0). Peptide identification was carried out using MSFragger (version 4.1) (Kong et al. 2017, Teo et al. 2021) using the default TMT16 workflow. In detail, the precursor mass tolerance was set to ± 20 ppm, the fragment mass tolerance to 20 ppm, as digestion enzyme Trypsin with a maximum of two missed cleavages and omission of proline rule was set. As fixed modifications, TMTpro labelling of lysine, and carbamidomethylation of cysteine was set. Variable modifications were set to TMTpro labelling of the peptide N-terminus, protein N-terminus and serine, with additionally oxidation of methionine and acetylation of the protein N-terminus. Peptide identifications were rescored using MSBooster (Yang et al. 2023), under application of the Prosit 2020 iRT TMT and Prosit 2020 Intensity TMT deep learning models (Gabriel et al. 2022) with subsequent application of the Percolator algorithm (Käll et al. 2007, The et al. 2016) for false discovery rate (FDR) calculation, which was set to 1% for peptide-to-spectrum matches (PSM). Protein identifications were carried out using ProteinProphet (version 5.1.1) (Nesvizhskii et al. 2003). Results (PSM, peptides, and proteins) were filtered with a 1% FDR using Philosopher (da Veiga Leprevost et al. 2020).

Peptide quantification data was processed and analysed in R (version 4.3.1) (R Core Team 2023) using the RStudio GUI (version 2024.04.1 Build 748) (Posit team 2024). In brief, TMT reporter ion intensities were extracted from the MS raw data and isotope corrected. Peptides which were identified several times (based on their sequence inclusive modifications) were averaged and the resulting intensities were VSN transformed and TMT channels affine calibrated (Huber et al. 2002, 2003). Statistical testing was carried out using limma (Ritchie et al. 2015) under application of robust hyperparameter estimation (Phipson et al. 2016). FDR for differentially abundant peptides was set to 5%. Network analysis was carried out using Reactome (Milacic et al. 2024) and STRING (Szklarczyk et al. 2023). Following R libraries were used: AnnotationHub (Morgan and Shepherd 2024), dplyr (Wickham et al. 2023a), extrafont (Chang 2023), ggnetwork (Briatte 2023), ggplot2 (Wickham 2016), (Kremer 2019), ggrepel (Slowikowski 2024), gtools (Warnes et al. 2023), igraph (Csardi and Nepusz 2006, Csárdi et al. 2024), limma (Ritchie et al. 2015), matrixStats (Bengtsson 2024), plyr (Wickham 2011), ReactomePA (Yu and He 2016, Milacic et al. 2024), reshape2 (Wickham 2007), scales (Wickham et al. 2023b), seqinr (Charif and Lobry 2007), STRINGdb (Szklarczyk et al. 2023), stringr (Wickham 2023), viridisLite (Garnier et al. 2023), and vsn (Huber et al. 2002). All proteomics mass spectrometry raw data and FragPipe search output can be accessed from jPOSTrepo with the project identifier JPST003689 and ProteomeXchange with the identifier PXD061716. All R scripts used for data processing, statistical testing and analysis can be downloaded from Zenodo with the URL https://doi.org/10.5281/zenodo.15040183.

### Sample preparation for lipidomics and metabolomics—methanol extraction

Samples were stored at −80°C from delivery to the start of the sample preparation. The samples were thawed on ice and 100 µl of sample was transferred to a fresh tube. To these tubes 900 µl of extraction buffer (composed of 80% methanol in H_2_O) was added. This mixture was kept overnight at −80°C to precipitate the present proteins.

### Liquid–liquid extraction

The methanol extract was employed for a liquid–liquid extraction to fractionate the samples. The methanol extract was centrifuged at 17 000 *× g* for 10 min, after which 400 µl of the supernatant was transferred to a fresh tube. To this, 400 µl of chloroform was added, followed by thorough vortexing for 60 s. An additional 130 µl of H_2_O was then added to induce phase separation, followed by another brief vortexing period of 15 s. This mixture was then subjected to centrifugation for 5 min at 5000 *× g*. Hereafter, 200 µl of the upper polar phase was transferred to an MS autosampler tube, while an equal volume of the lower apolar phase was dried using a CentriVap system (Labconco, USA). The resulting dried pellet was then reconstituted in 100% methanol, before being transferred to an MS autosampler tube. Lastly, a QC sample was prepared by pooling 10 µl of each phase.

### MS method—lipidomics and metabolomics

Mass spectrometry measurements were performed using an Infinity ll autosampler and pump System (Agilent, USA) coupled to a ScimaX 7T Magnetic Resonance Mass Spectrometer (Bruker, USA) operated in both negative and positive mode. 10 µl sample was injected by flow injection analysis. The pushing solvent used was 80% 2-propanol. The samples were measured in both positive and negative mode and in technical triplicates. A QC sample was measured before, in the middle and after the samples to account for batch control variation. The mass spectrometer operated in full scan mode [range (107.0000–1000.0000)], 35 scans were averaged to one mass spectrum at a resolution of ±1 million at 120 m/z, using the following parameters: Spray voltage was set to 4.2 kV (positive mode) or −4.8 kV (negative mode) with a drying gas flow of 4 l/min. Nebulizer gas pressure was 1.5 bar. DC extract bias was set to −1.3 (positive mode) or 1 (negative mode). The transfer capillary was operated at 200°C. Ion accumulation time was set to 0.05 s, while the collision voltage was set to 1.5 or −1.5 V for positive and negative mode, respectively, TOF was set to 0.5 s.

### Data analysis—lipidomics

The mass spectrometry raw data was processed using the ‘Statistical’ algorithm within the ftmsControl software (version 2.3.0–01_21_2020_10, Bruker, Billerica, MA, USA). Peak picking was performed using a minimal signal-to-noise ratio of 3 and a minimum intensity of 1 × 10^5^. The annotation of the detected features was conducted using MetaboScep 2022b (version 9.0.1, Bruker) with the MCube Lipid Species library (Bruker), with a mass error of maximum 5 ppm and a mSigma value < 250. Raw feature intensity data was further processed in R (v. 4.3.1) (R Core Team 2023) using the RStudio GUI (version 2024.04.1 Build 748) (Posit team 2024). In brief, the intensities of lipids with replicate measurements within a sample were averaged and transformed using EigenMS (Karpievitch et al. 2014). Statistical testing was carried out using limma (Ritchie et al. 2015) under application of robust hyperparameter estimation (Phipson et al. 2016). Lipids were regarded as differentially abundant if they exhibited an adjusted *P*-value ≤ .05 (5% FDR). Network analysis of differentially abundant lipid molecular species and lipid classes was carried out using bioPAN (Ni and Fedorova 2020, Gaud et al. 2021) (https://www.lipidmaps.org/biopan/). All MS raw data and database search engine output can be downloaded from Metabolights (MTBLS11283). All R scripts used for data processing, statistical testing and analysis can be downloaded from Zenodo with the URL https://doi.org/10.5281/zenodo.15040183. Following R libraries were used: ProteoMM (Karpievitch et al. 2023) and xlsx (Dragulescu and Arendt 2020).

### Data analysis—metabolomics

Raw mass spectrometry data and feature intensity data was processed as described in the lipidomics section. For feature matching, the Human Metabolome Database (downloaded 26 October 2022 from https://hmdb.ca/) with a maximum mass error of 0.8 ppm and a mSigma value <250 was applied. Network analysis was carried out using FELLA (Picart-Armada et al. 2017, 2018). Mass spectrometry raw data and metabolite compound search engine output can be accessed from Metabolights (MTBLS11283). All R scripts used for data processing, statistical testing and analysis can be downloaded from Zenodo with the URL https://doi.org/10.5281/zenodo.15040183. Following R libraries were used: biomaRt (Durinck et al. 2005, 2009) and FELLA (Picart-Armada et al. 2017, 2018).

## Results

**Figure 1.**
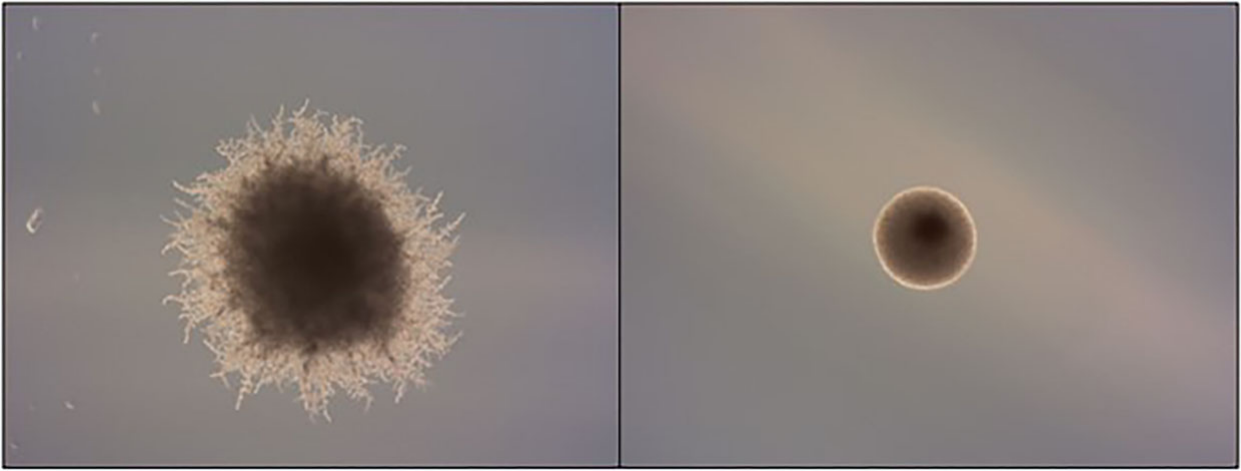
Examples of colony morphology of industrial *S. cerevisiae* strains cultivation on nitrogen-limiting SLAD medium. Strains YMD4537 (left) and YMD4544 (right) cultivated on nitrogen-limiting SLAD medium, photographed at 2.5x magnification.

Industrial strains of *S. cerevisiae* (*n* = 3) were cultivated on SLAD and SLAD supplemented with 2-PE (200 μM) to investigate the effects of fungal QS molecule 2-PE under nitrogen-restrictive conditions. This was compared to growth on nitrogen-rich agar plates (SHAD and SHAD-2-PE). Pseudofilamentous growth can be induced under nitrogen-limiting conditions and is further enhanced by exogenous 2-PE exposure (Britton et al. 2023). Nitrogen starvation in the presence of 2-PE was previously shown to boost filamentous growth in strains YMD4529 and YMD4537, while strain YMD4544 failed to yield filaments under any condition (Britton et al. 2023), as illustrated by the examples of filamentation and nonfilamentation in Fig. 1. This notable variation in behavior among the strains was further explored through proteomic, lipidomic, and metabolomic analyses, aiming to elucidate the relationship between these biochemical changes and the observed phenotypic morphogenic transitions.

### Proteomics

Each yeast strain was labelled using TMT16plex, thus four biological replicates of the four growth conditions for each strain could be analysed in a single experiment (3 yeast strains = 3 individual mass spectrometry analyses). Proteome analysis resulted in the identification and quantification of 46 826 PSM (10 086 unique peptides) and 1975 unique proteins (5% FDR). The analysis of differentially abundant peptides revealed significant variations between SLAD and SHAD growth conditions in strains YMD4529 and YMD4537 (refer to Fig. S1). Interestingly, the yeast strain YMD4544 demonstrated no changes in peptide abundance, a result that is consistent with the lack of morphogenic switching and filamentous growth observed under nitrogen starvation conditions (Britton et al. 2022, 2023). Table 1 presents the top five peptides exhibiting differential abundance between the SLAD and SHAD conditions (all peptides are shown in Tables S1–S3). There was no consistent detectable impact of 2-PE on the proteome across the three yeast strains, at concentrations ordinarily observed in fermentation environments (Ayrapaa 1961, Szlavko 1973, Li et al. 2008, Zupan et al. 2013, Avbelj et al. 2015), under both nitrogen-rich and nitrogen-starved conditions. (see Fig. S1).

**Table 1.** Top five differentially abundant peptides under all studied conditions. The logFC gives the change in abundance of the identified peptide (adj. *P*-value ≤ .05), with a positive logFC indicating a higher abundant peptide in the first named condition in the column ‘Comparison’.

Comparison	Yeast	Uniprot account number	Gene name	Protein name	Peptide sequence	adj. *P*-value	logFC
SLAD vs SHAD	YMD4529	P22943	*HSP12*	12 kDa heat shock protein	LNDAVEYVSGR	0.0020	3.14
		P40169	*YNL194C*	Uncharacterized plasma membrane protein YNL194C	TVTSAGDEPDRVQEER	0.0003	3.07
		P09232	*PRB1*	Cerevisin	NANVVAVK	0.0020	2.97
		P19145	*GAP1*	General amino-acid permease GAP1	ALAAQGR	0.0013	2.68
		P28240	*ICL1*	Isocitrate lyase	AYAQNVQQR	0.0013	2.59
		P30605	*ITR1*	Myo-inositol transporter 1	SYTDTSEEIIER	0.0003	−2.59
		P10127	*ADH4*	Alcohol dehydrogenase 4	AALPLFAINTTAGTASEMTR	0.0005	−1.95
		P39015	*STM1*	Suppressor protein STM1	NNFNNRR	0.0032	−1.81
		P38061	*RPL32*	Large ribosomal subunit protein eL32	HHSDRYHR	0.0363	−1.75
		P17967	*PDI1*	Protein disulfide-isomerase	NMAPEYVK	0.0048	−1.73
	YMD4537	P36139	*PET10*	Protein PET10	ASREDQTNSKPAAVSTN	0.0012	3.66
		Q06177	*TMA10*	Translation machinery-associated protein 10	RGSNSQNNERR	0.0001	3.23
		P22943	*HSP12*	12 kDa heat shock protein	SKLNDAVEYVSGR	0.0022	3.20
		P38109	*ATG42*	Vacuolar serine-type carboxypeptidase ATG42	GPCEDNSTDGMCYTGLR	0.0000	2.94
		Q03558	*OYE2*	NADPH dehydrogenase 2	TLIGYGR	0.0001	2.92
		P11986	*INO1*	Inositol-3-phosphate synthase	EGVKQPNYFGSMTQCSTLK	0.0157	−2.61
		P42948	*SET4*	SET domain-containing protein 4	TSPESLSSR	0.0034	−2.12
		P38061	*RPL32*	Large ribosomal subunit protein eL32	DRYHR	0.0176	−2.12
		P10127	*ADH4*	Alcohol dehydrogenase 4	ALIVTDPGIAAIGLSGR	0.0081	−2.05
		P05750	*RPS3*	Small ribosomal subunit protein uS3	FKLLNGLAIRR	0.0005	−1.84
	YMD4544	–	–	–	–	–	–
SLAD-2PE vs SHAD-2PE	YMD4529	P40169	*YNL194C*	Uncharacterized plasma membrane protein YNL194C	TVTSAGDEPDRVQEER	0.0003	3.04
		P09232	*PRB1*	Cerevisin	NANVVAVK	0.0014	2.79
		P22943	*HSP12*	12 kDa heat shock protein	LNDAVEYVSGR	0.0032	2.67
		P56508	*SNA2*	Protein SNA2	YSHLSSSDDNYGSLA	0.0003	2.50
		P28240	*ICL1*	Isocitrate lyase	AYAQNVQQR	0.0014	2.45
		P30605	*ITR1*	Myo-inositol transporter 1	SYTDTSEEIIER	0.0001	−2.98
		P39015	*STM1*	Suppressor protein STM1	NNFNNRR	0.0005	−2.26
		P38061	*RPL32*	Large ribosomal subunit protein eL32	HHSDRYHR	0.0085	−2.17
		Q08412	*CUE5*	Ubiquitin-binding protein CUE5	RRHNPNER	0.0203	−2.01
		P10127	*ADH4*	Alcohol dehydrogenase 4	AALPLFAINTTAGTASEMTR	0.0003	−1.92
	YMD4537	P22943	*HSP12*	12 kDa heat shock protein	GKDNAEGQGESLADQAR	0.0293	3.65
		P38109	*ATG42*	Vacuolar serine-type carboxypeptidase ATG42	GPCEDNSTDGMCYTGLR	0.0000	3.07
		Q06177	*TMA10*	Translation machinery-associated protein 10	RGSNSQNNER	0.0064	3.04
		P00445	*SOD1*	Superoxide dismutase [Cu–Zn]	GNVKTDENGVAK	0.0008	2.75
		P36139	*PET10*	Protein PET10	TKLNETYQR	0.0001	2.55
		P11986	*INO1*	Inositol-3-phosphate synthase	KVDHCIVIK	0.0073	−2.15
		P10127	*ADH4*	Alcohol dehydrogenase 4	EQVVAIIKK	0.0117	−2.11
		P30605	*ITR1*	Myo-inositol transporter 1	SYTDTSEEIIER	0.0457	−1.91
		P02406	*RPL28*	Large ribosomal subunit protein uL15	HRGHVSAGK	0.0011	−1.90
		P00360	*TDH1*	Glyceraldehyde-3-phosphate dehydrogenase 1	LISWYDNEYGYSAR	0.0094	−1.89
	YMD4544	P04076	*ARG4*	Argininosuccinate lyase	NADSLELLRGK	0.0285	1.99
		P53252	*PIL1*	Sphingolipid long chain base-responsive protein PIL1	SMELTANERR	0.0224	1.78
		P47137	*YJR096W*	Uncharacterized oxidoreductase YJR096W	LRLETWR	0.0224	1.71
		P16649	*TUP1*	General transcriptional corepressor TUP1	LSDDSAANNHR	0.0224	1.55
		P40446	*YIL165C*	Putative nitrilase-like protein YIL165C	FDLDPVGHYAR	0.0306	1.52
		P11986	*INO1*	Inositol-3-phosphate synthase	YMKPVGDSK	0.0363	−3.17
		P00360	*TDH1*	Glyceraldehyde-3-phosphate dehydrogenase 1	LISWYDNEYGYSAR	0.0224	−2.71
		P17967	*PDI1*	Protein disulfide-isomerase	NSDVNNSIDYEGPR	0.0224	−2.64
		P49166	*RPL37A*	Large ribosomal subunit protein eL37A	HNKSHTLCNR	0.0337	−2.62
		P10127	*ADH4*	Alcohol dehydrogenase 4	KAALPLFAINTTAGTASEMTR	0.0470	−2.53
SLAD-2PE vs SHAD	YMD4529	P22943	*HSP12*	12 kDa heat shock protein	LNDAVEYVSGR	0.0009	3.27
		P40169	*YNL194C*	Uncharacterized plasma membrane protein YNL194C	TVTSAGDEPDRVQEER	0.0003	3.20
		Q06177	*TMA10*	Translation machinery-associated protein 10	RGSNSQNNERR	0.0090	2.52
		P28240	*ICL1*	Isocitrate lyase	AYAQNVQQR	0.0011	2.48
		P36139	*PET10*	Protein PET10	LNETYQR	0.0003	2.47
		P30605	*ITR1*	Myo-inositol transporter 1	SYTDTSEEIIER	0.0001	−3.11
		P39015	*STM1*	Suppressor protein STM1	NNFNNRR	0.0004	−2.29
		P00950	*GPM1*	Phosphoglycerate mutase 1	GQQEAAR	0.0076	−2.04
		P10127	*ADH4*	Alcohol dehydrogenase 4	AALPLFAINTTAGTASEMTR	0.0003	−1.93
		Q08412	*CUE5*	Ubiquitin-binding protein CUE5	RRHNPNER	0.0353	−1.80
	YMD4537	P38109	*ATG42*	Vacuolar serine-type carboxypeptidase ATG42	GPCEDNSTDGMCYTGLR	0.0000	3.03
		P22943	*HSP12*	12 kDa heat shock protein	ASEALKPDSQK	0.0003	3.01
		P09232	*PRB1*	Cerevisin	TIPLNDEDLDGNGHGTHCAGTIASK	0.0000	2.96
		P00445	*SOD1*	Superoxide dismutase [Cu–Zn]	GNVKTDENGVAK	0.0008	2.94
		P36139	*PET10*	Protein PET10	ASREDQTNSKPAAVSTN	0.0231	2.65
		P10127	*ADH4*	Alcohol dehydrogenase 4	EQVVAIIKK	0.0078	−2.25
		P11986	*INO1*	Inositol-3-phosphate synthase	KVDHCIVIK	0.0087	−2.08
		P42948	*SET4*	SET domain-containing protein 4	TSPESLSSR	0.0068	−2.05
		P30605	*ITR1*	Myo-inositol transporter 1	SYTDTSEEIIER	0.0381	−2.01
		P38061	*RPL32*	Large ribosomal subunit protein eL32	DRYHR	0.0405	−1.84
	YMD4544	P40446	*YIL165C*	Putative nitrilase-like protein YIL165C	NIAYEGR	0.0213	2.46
		P54867	*SLG1*	Protein SLG1	ADSYNWQSSSHCNSECSAK	0.0222	2.29
		P09232	*PRB1*	Cerevisin	GVEYAAK	0.0262	2.20
		P05030	*PMA1*	Plasma membrane ATPase 1	VSTQHEKET	0.0481	2.12
		Q03558	*OYE2*	NADPH dehydrogenase 2	FFISNPDLVDRLEK	0.0262	2.10
		P11986	*INO1*	Inositol-3-phosphate synthase	VVTDKCTYKDNELLTK	0.0203	−3.47
		P0CX49	*RPL18A*	Large ribosomal subunit protein eL18A	GIDHTSKQHKR	0.0194	−2.75
		P00359	*TDH3*	Glyceraldehyde-3-phosphate dehydrogenase 3	YAGEVSHDDKHIIVDGKK	0.0481	−2.69
		P10127	*ADH4*	Alcohol dehydrogenase 4	KAALPLFAINTTAGTASEMTR	0.0201	−2.64
		P00360	*TDH1*	Glyceraldehyde-3-phosphate dehydrogenase 1	LISWYDNEYGYSAR	0.0142	−2.59

**Figure 2.**
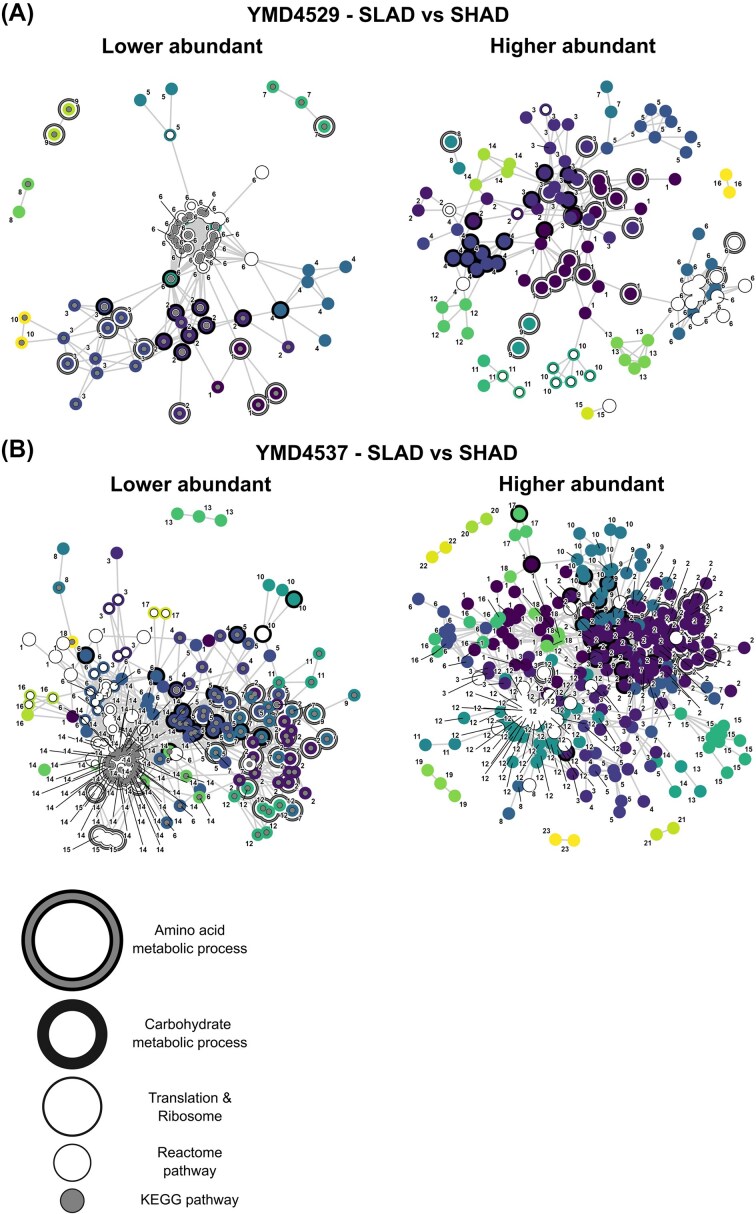
Interaction networks of proteins with differentially abundant peptides. (A) Yeast strain YMD4529 and (B) YMD4537. Comparison of SLAD versus SHAD conditions, with left side lower abundant peptides under SLAD conditions, and right side higher abundant peptides under growth on SLAD medium (adj. *P*-value ≤ .05). Protein with known interactions are connected by edges, numbers, and colours indicate membership of respective network cluster, decorations indicate protein membership in GO terms associated with amino acid- and carbohydrate metabolic processes, translation and ribosomes as indicated below the figure. Proteins involved in specific Reactome and KEGG pathways are indicated as well. Individual proteins within each network graph can be seen in Fig. S2, while GO terms and pathways are listed in Table S4.

Gene ontology (GO) term analysis conducted via the STRING database revealed significant enrichment (5% FDR) in protein biological processes, molecular functions, and cellular components exclusively under conditions of nitrogen starvation, regardless of the presence of 2-PE. In proteins comprised of lower abundant peptides (see Tables S1–S3), significant GO terms were detected for growth conditions in YMD4537 and YMD4539. The terms exhibit considerable overlap among the strains. Within the biological process GO category, the most significant shared high-level terms between strains YMD4529 and YMD4537 include GO:1901566 (organonitrogen compound biosynthetic process), GO:1901576 (organic substance biosynthetic process), GO:0044249 (cellular biosynthetic process), GO:1901564 (organonitrogen compound metabolic process), and GO:0006412 (translation). The analysis of molecular function and cellular components through GO revealed a notable enrichment of GO terms associated with ribosomes in the same two strains (YMD4537 and YMD4529). In contrast, for strain YMD4544, ribosomal-related GO terms were enriched exclusively in the comparison between SLAD-2-PE and SHAD (refer to Table 2). While significant enrichment via GO terms was not consistently observed—specifically in the cases of YMD4529 and YMD4537 when comparing SLAD-2-PE to SHAD, as well as for YMD4544 across all comparisons involving significant cellular component GO terms—the STRING network interaction graphs underscore a robust participation of ribosomal proteins across all growth conditions examined (see Fig. 2). As for proteins with more abundant peptides under nitrogen starvation, only in the SLAD-2-PE versus SHAD comparison in YMD4537 a substantial enrichment in the molecular function ‘oxidoreductase activity’ was identified (GO:0016491).

**Table 2.** Top five enriched GO terms, KEGG, and Reactome pathways. Peptide regulation group indicates if the peptide is higher or lower abundant in the first named condition in the comparison column. The term column contains the GO terms, or pathway abbreviations. FDR depicts the false-discovery rate of the enrichment, the enrichment factor the magnitude of the enrichment.

Comparison	Yeast	Class	Category	Peptide regulation group	Term	Description	FDR	FC
SLAD vs SHAD	YMD4529	GO	Process	Down	GO:0032787	Monocarboxylic acid metabolic process	0.0138	3.13
			Component	Down	GO:0022626	Cytosolic ribosome	0.0006	3.08
			Function	Down	GO:0003735	Structural constituent of ribosome	0.0103	2.82
			Component	Down	GO:0044391	Ribosomal subunit	0.0039	2.73
			Process	Down	GO:0002181	Cytoplasmic translation	0.0124	2.61
		KEGG		Down	sce00680	Methane metabolism	0.0356	5.22
				Down	sce00620	Pyruvate metabolism	0.0223	4.14
				Down	sce00010	Glycolysis/gluconeogenesis	0.0356	3.35
				Down	sce03010	Ribosome	0.0006	3.1
				Down	sce01230	Biosynthesis of amino acids	0.0223	2.53
		Reactome		Down	R-SCE-70263	Gluconeogenesis	0.0134	3.77
				Down	R-SCE-70326	Glucose metabolism	0.0241	2.88
				Down	R-SCE-72689	Formation of a pool of free 40S subunits	0.0000	2.85
				Down	R-SCE-72706	GTP hydrolysis and joining of the 60S ribosomal subunit	0.0000	2.85
				Down	R-SCE-1799339	SRP-dependent cotranslational protein targeting to membrane	0.0000	2.81
				Up	R-SCE-382551	Transport of small molecules	0.0005	3.63
	YMD4537	GO	Function	Down	GO:0140662	ATP-dependent protein folding chaperone	0.0396	3.61
			Component	Down	GO:0022627	Cytosolic small ribosomal subunit	0.0007	3.26
			Component	Down	GO:0022626	Cytosolic ribosome	0.0000	2.72
			Component	Down	GO:0044391	Ribosomal subunit	0.0000	2.56
			Function	Down	GO:0003735	Structural constituent of ribosome	0.0000	2.56
		KEGG		Down	sce03010	Ribosome	0.0000	2.68
				Down	sce01230	Biosynthesis of amino acids	0.0092	2.11
				Down	sce01110	Biosynthesis of secondary metabolites	0.0203	1.61
				Down	sce01100	Metabolic pathways	0.0368	1.39
		Reactome		Down	R-SCE-72706	GTP hydrolysis and joining of the 60S ribosomal subunit	0.0000	2.71
				Down	R-SCE-72689	Formation of a pool of free 40S subunits	0.0000	2.65
				Down	R-SCE-975956	Nonsense mediated decay (NMD) independent of the exon junction complex (EJC)	0.0000	2.64
				Down	R-SCE-1799339	SRP-dependent cotranslational protein targeting to membrane	0.0000	2.58
				Down	R-SCE-927802	Nonsense-mediated decay (NMD)	0.0000	2.54
	YMD4544	–	–	–	–	–	–	–
SLAD-2PE vs SHAD	YMD4529	GO	Function	Down	GO:0019843	rRNA binding	0.0397	4.65
			Process	Down	GO:0032787	Monocarboxylic acid metabolic process	0.0381	3.25
			Process	Down	GO:0044283	Small molecule biosynthetic process	0.0136	2.59
			Process	Down	GO:0019752	Carboxylic acid metabolic process	0.0136	2.31
			Component	Down	GO:0005829	Cytosol	0.0010	1.92
		KEGG		Down	sce00010	Glycolysis/gluconeogenesis	0.0183	3.98
				Down	sce01230	Biosynthesis of amino acids	0.0035	3.01
				Down	sce01110	Biosynthesis of secondary metabolites	0.0001	2.52
				Down	sce01100	Metabolic pathways	0.0004	1.94
		Reactome		Down	R-SCE-70263	Gluconeogenesis	0.0097	4.63
				Down	R-SCE-211859	Biological oxidations	0.0125	4.32
				Down	R-SCE-70171	Glycolysis	0.0354	3.41
				Down	R-SCE-72689	Formation of a pool of free 40S subunits	0.0022	2.63
				Down	R-SCE-72706	GTP hydrolysis and joining of the 60S ribosomal subunit	0.0022	2.63
				Up	R-SCE-382551	Transport of small molecules	0.0008	3.53
	YMD4537	GO	Process	Down	GO:0006407	rRNA export from nucleus	0.0265	7.47
			Process	Down	GO:0097064	ncRNA export from nucleus	0.0265	5.75
			Process	Down	GO:0046394	Carboxylic acid biosynthetic process	0.0265	2.59
			Process	Down	GO:0044283	Small molecule biosynthetic process	0.0265	2.18
			Process	Down	GO:1901566	Organonitrogen compound biosynthetic process	0.0000	2.04
			Function	Up	GO:0016491	Oxidoreductase activity	0.0031	2.02
		KEGG		Down	sce01230	Biosynthesis of amino acids	0.0136	2.43
				Down	sce03010	Ribosome	0.0136	2.43
				Down	sce01110	Biosynthesis of secondary metabolites	0.0074	1.98
				Down	sce01100	Metabolic pathways	0.0108	1.63
				Up	sce00010	Glycolysis/gluconeogenesis	0.0411	2.61
				Up	sce01110	Biosynthesis of secondary metabolites	0.0157	1.69
				Up	sce01100	Metabolic pathways	0.0157	1.46
		Reactome		Down	R-SCE-72706	GTP hydrolysis and joining of the 60S ribosomal subunit	0.0005	2.67
				Down	R-SCE-72689	Formation of a pool of free 40S subunits	0.0010	2.54
				Down	R-SCE-72695	Formation of the ternary complex, and subsequently, the 43S complex	0.0230	2.5
				Down	R-SCE-1799339	SRP-dependent cotranslational protein targeting to membrane	0.0011	2.47
				Down	R-SCE-975956	Nonsense-mediated decay (NMD) independent of the exon junction complex (EJC)	0.0012	2.4
				Up	R-SCE-1430728	Metabolism	0.0046	1.57
	YMD4544	GO	Process	Down	GO:0006407	rRNA export from nucleus	0.0301	5.31
			Process	Down	GO:0000028	Ribosomal small subunit assembly	0.0381	4.95
			Component	Down	GO:0022627	Cytosolic small ribosomal subunit	0.0002	3.63
			Process	Down	GO:0042255	Ribosome assembly	0.0332	3.1
			Component	Down	GO:0022626	Cytosolic ribosome	0.0000	2.9
		KEGG		Down	sce01210	2-Oxocarboxylic acid metabolism	0.0219	3.36
				Down	sce00010	Glycolysis/gluconeogenesis	0.0072	3.1
				Down	sce03010	Ribosome	0.0001	2.7
				Down	sce01200	Carbon metabolism	0.0013	2.68
				Down	sce01230	Biosynthesis of amino acids	0.0009	2.47
				Up	sce01210	2-Oxocarboxylic acid metabolism	0.0350	3.49
		Reactome		Down	R-SCE-211859	Biological oxidations	0.0247	2.64
				Down	R-SCE-72695	Formation of the ternary complex, and subsequently, the 43S complex	0.0001	2.61
				Down	R-SCE-72689	Formation of a pool of free 40S subunits	0.0000	2.52
				Down	R-SCE-72706	GTP hydrolysis and joining of the 60S ribosomal subunit	0.0000	2.52
				Down	R-SCE-70171	Glycolysis	0.0222	2.51
SLAD-2PE vs SHAD-2PE	YMD4529	GO	Component	Down	GO:0022626	Cytosolic ribosome	0.0309	2.74
			Component	Down	GO:0005829	Cytosol	0.0000	2.02
			Process	Down	GO:1901566	Organonitrogen compound biosynthetic process	0.0373	1.75
			Process	Down	GO:0009058	Biosynthetic process	0.0008	1.73
			Process	Down	GO:1901576	Organic substance biosynthetic process	0.0008	1.73
		KEGG		Down	sce00010	Glycolysis/Gluconeogenesis	0.0330	3.82
				Down	sce01110	Biosynthesis of secondary metabolites	0.0034	2.25
				Down	sce01100	Metabolic pathways	0.0092	1.76
		Reactome		Down	R-SCE-70263	Gluconeogenesis	0.0082	4.63
				Down	R-SCE-72689	Formation of a pool of free 40S subunits	0.0019	2.63
				Down	R-SCE-72706	GTP hydrolysis and joining of the 60S ribosomal subunit	0.0019	2.63
				Down	R-SCE-1799339	SRP-dependent cotranslational protein targeting to membrane	0.0019	2.59
				Down	R-SCE-975956	Nonsense-mediated decay (NMD) independent of the exon junction complex (EJC)	0.0023	2.49
				Up	R-SCE-382551	Transport of small molecules	0.0169	3.34
	YMD4537	GO	Component	Down	GO:0022627	Cytosolic small ribosomal subunit	0.0256	3.58
			Component	Down	GO:0022626	Cytosolic ribosome	0.0132	2.59
			Function	Down	GO:0003735	Structural constituent of ribosome	0.0468	2.55
			Component	Down	GO:0044391	Ribosomal subunit	0.0200	2.44
			Process	Down	GO:0002181	Cytoplasmic translation	0.0266	2.4
			Function	Up	GO:0016491	Oxidoreductase activity	0.0177	1.93
		KEGG		Down	sce01230	Biosynthesis of amino acids	0.0172	2.59
				Down	sce03010	Ribosome	0.0172	2.59
				Down	sce01110	Biosynthesis of secondary metabolites	0.0172	1.94
				Down	sce01100	Metabolic pathways	0.0172	1.59
				Up	sce01110	Biosynthesis of secondary metabolites	0.0339	1.65
		Reactome		Down	R-SCE-70263	Gluconeogenesis	0.0050	4.18
				Down	R-SCE-70326	Glucose metabolism	0.0139	3.1
				Down	R-SCE-72695	Formation of the ternary complex, and subsequently, the 43S complex	0.0008	3
				Down	R-SCE-72649	Translation initiation complex formation	0.0007	2.88
				Down	R-SCE-72662	Activation of the mRNA upon binding of the cap-binding complex and eIFs, and subsequent binding to 43S	0.0007	2.88
	YMD4544	GO	Process	Down	GO:0046394	Carboxylic acid biosynthetic process	0.0042	3.64
			Process	Down	GO:0044283	Small molecule biosynthetic process	0.0001	3.47
			Process	Down	GO:1901605	Alpha-amino acid metabolic process	0.0436	3.16
			Process	Down	GO:0019752	Carboxylic acid metabolic process	0.0009	2.78
			Component	Down	GO:0005829	Cytosol	0.0001	2.22
		KEGG		Down	sce03010	Ribosome	0.0031	3.43
				Down	sce01230	Biosynthesis of amino acids	0.0160	3.06
				Down	sce01110	Biosynthesis of secondary metabolites	0.0003	2.78
				Down	sce01100	Metabolic pathways	0.0031	1.98
				Up	sce01210	2-Oxocarboxylic acid metabolism	0.0076	6.54
				Up	sce01230	Biosynthesis of amino acids	0.0284	2.96
				Up	sce01110	Biosynthesis of secondary metabolites	0.0284	2.15
				Up	sce01100	Metabolic pathways	0.0434	1.7
		Reactome		Down	R-SCE-70263	Gluconeogenesis	0.0300	4.36
				Down	R-SCE-72695	Formation of the ternary complex, and subsequently, the 43S complex	0.0300	2.74
				Down	R-SCE-72689	Formation of a pool of free 40S subunits	0.0179	2.48
				Down	R-SCE-72706	GTP hydrolysis and joining of the 60S ribosomal subunit	0.0179	2.48
				Down	R-SCE-1799339	SRP-dependent cotranslational protein targeting to membrane	0.0179	2.44

The effect of nitrogen starvation on ribosomes is supported by KEGG pathway analysis, which identified the term sce03010 (ribosome) for proteins exhibiting reduced peptide abundance under nitrogen-limited conditions in YMD4537 and YMD4529. Additionally, in the presence of 2-PE, this trend was also observed in YMD4544. Intriguingly, the addition of 2-PE under nitrogen starvation conditions in YMD4529 resulted in the absence of the ribosome KEGG pathway. However, pathways related to metabolite synthesis and glycolysis/gluconeogenesis continued to demonstrate significance (see Table 2). Despite the lack of significant GO term enrichment, a clear cluster of ribosomal interaction partners is detected here in the network analysis graph as well (see Fig. 2). In instances, where notable KEGG terms were identified in proteins featuring highly abundant peptides under nitrogen starvation conditions (specifically YMD4537 and YMD4544 SLAD-2-PE in comparison to SHAD, as well as YMD4537 SLAD-2-PE versus SHAD-2-PE), the same pathways emerged, albeit with the exclusion of those related to ribosomal functions. Nevertheless, the accompanying network graphs reveal the presence of clusters associated with ribosomal proteins for these comparisons (refer to Fig. 2). Further pathway analysis utilizing Reactome (with a 5% FDR) revealed that processes such as translation, ribosomal assembly, and nonsense-mediated decay were significantly enriched in proteins characterized by peptides in lower-abundance during nitrogen starvation, regardless of the addition of 2-PE. In contrast, pathways related to gluconeogenesis and glucose metabolism were notably significant in YMD4529, while this association was observed in YMD4537 solely in the presence of 2-PE (see Table 2). As for proteins with peptides that were more abundant under nitrogen starvation, transport of small molecules (R-SCE-382551) was the only significant term in YMD4529 (independent of 2-PE addition) and while in YMD4537 (SLAD-2-PE versus SHAD) the term metabolism (R-SCE-1430728) emerged.

Finally, A STRING interaction network analysis was conducted to identify protein interaction clusters among the differentially abundant peptides for each experimental condition, utilizing only high-confidence interactions with a score of 700 or higher. This network was combined with the GO term and pathway analysis to reveal differences in the yeast strains in response to nitrogen starvation and in combination with 2-PE. In line with the GO term and pathway analysis, interaction clusters of ribosomal and translation related proteins [GO:0006412 (translation) and offspring terms], glucose metabolism, glycolysis, and gluconeogenesis [GO:0005975 (carbohydrate metabolic process) and offspring terms], and amino acid metabolism [GO:0006520 (amino acid metabolic process) and offspring terms] are evident (see Fig. 2 and Table S4). Only a minor set of proteins exhibited peptides which were simultaneously detected in the lower and higher abundance groups under nitrogen starvation. Most of these are involved in amino acid- and ribosome metabolism (see Table 3). Two proteins (LEU2 and ICL1) exhibited a set of peptides in lower abundance and a second set in higher abundance under nitrogen starvation in YMD4529 and YMD4537. Interestingly, in the comparison YMD4544 SLAD-2PE versus SHAD, the only E1 Ubiquitin activating enzyme of *S. cerevisiae* UBA1 was detected with a lower abundant peptide (GNTQVIIPR, AA573-581) under nitrogen starvation, but in YMD4537 SLAD versus SHAD a more abundant peptide (VEVLGPFAFR, AA230–239) for this protein was detected under these conditions. This can be interpreted as a hint for possible posttranslational modifications of this protein (all proteins with peptides up- and downregulated under the same experimental conditions are listed in Table S13).

**Table 3.** Top five differentially abundant metabolites under all studied conditions. The logFC gives the change in abundance of the identified metabolite (adj. *P*-value ≤ .05), with a positive logFC indicating a higher abundant peptide in the first named condition in the column ‘Comparison’. The m/z column shows the experimentally observed mass-to-charge ratio of the metabolite.

Comparison	Yeast	m/z	Metabolite name	adj. *P-*value	logFC
SLAD vs SHAD	YMD4529	191.01982	Diketogulonic acid	2.92E-06	4.01
		203.05261	d-Glucose	3.51E-06	2.32
		275.12493	Saccharopine	2.98E-05	2.20
		230.99023	Citric acid	6.53E-05	2.16
		229.03206	Homocitric acid	2.92E-06	2.12
		146.04597	l-Glutamic acid	2.08E-07	−1.81
		346.05593	2'-Deoxyguanosine 5'-monophosphate	8.14E-06	−1.78
		233.12436	l-Acetopine_negative	0.0395	−1.69
		214.04846	Glycerylphosphorylethanolamine	4.66E-05	−1.45
		152.03186	Pyroglutamic acid_negative	2.92E-06	−1.44
	YMD4537	191.01982	Diketogulonic acid	8.44E-09	3.53
		243.05279	l-Tryptophan	1.80E-09	2.39
		132.03024	d-Aspartic acid	5.30E-08	2.14
		302.06623	Deoxyguanosine	1.45E-06	2.14
		175.06127	2-Isopropylmalic acid	4.44E-07	2.02
		233.12436	l-Acetopine_negative	0.0020	−1.92
		346.05593	2'-Deoxyguanosine 5'-monophosphate	4.50E-08	−1.92
		131.0104	Glycerol	0.0001	−1.43
		131.01037	1_2-Benzoquinone	0.0025	−0.87
		173.04228	d-Ribose	0.0000	−0.84
	YMD4544	191.01982	Diketogulonic acid	7.77E-12	4.09
		203.05261	d-Glucose	3.99E-06	3.86
		147.11278	l-Lysine	1.31E-11	2.15
		243.06233	Uridine	6.39E-11	2.08
		268.10407	Adenosine	0.0087	1.76
		142.05111	Trimethadione	1.65E-13	−3.79
		233.12436	l-Acetopine_negative	3.12E-09	−3.59
		229.01211	5-Keto-d-gluconate	6.89E-13	−3.42
		190.05027	5-Phenyl-1_3-oxazinane-2_4-dione	1.23E-10	−3.13
		175.00174	Hypoxanthine	7.77E-12	−2.97
SLAD-2PE vs SHAD-2PE	YMD4529	191.01982	Diketogulonic acid	1.34E-07	5.29
		203.05261	d-Glucose	6.36E-06	2.11
		230.99023	Citric acid	0.0008	1.77
		229.03206	Homocitric acid	7.30E-06	1.72
		275.12493	Saccharopine	0.0004	1.58
		146.04597	l-Glutamic acid	6.03E-09	−2.35
		346.05593	2'-Deoxyguanosine 5'-monophosphate	3.86E-07	−2.34
		214.04846	Glycerylphosphorylethanolamine	1.95E-06	−1.85
		152.03186	Pyroglutamic acid_negative	3.05E-07	−1.75
		186.01645	l-Glutamic acid	5.64E-06	−1.55
	YMD4537	191.01982	Diketogulonic acid	5.45E-10	4.28
		243.05279	l-Tryptophan	5.45E-10	2.31
		230.99023	Citric acid	0.0022	2.12
		132.03024	d-Aspartic acid	8.93E-08	2.04
		147.11278	l-Lysine	5.52E-08	1.92
		233.12436	l-Acetopine_negative	0.0002	−2.46
		346.05593	2'-Deoxyguanosine 5'-monophosphate	4.02E-09	−2.27
		131.0104	Glycerol	1.56E-05	−1.77
		133.09705	Ornithine	0.0003	−1.49
		173.04228	d-Ribose	1.45E-07	−1.42
	YMD4544	191.01982	Diketogulonic acid	1.19E-11	3.86
		203.05261	d-Glucose	7.16E-05	2.84
		147.11278	l-Lysine	5.82E-11	1.91
		268.10407	Adenosine	0.0445	1.29
		281.05473	Aspartyl-Hydroxyproline	5.63E-05	1.16
		142.05111	Trimethadione	1.60E-13	−3.61
		214.04846	Glycerylphosphorylethanolamine	9.36E-14	−3.45
		229.01211	5-Keto-d-gluconate	1.90E-12	−3.35
		306.07656	Glutathione	6.89E-14	−3.26
		275.12493	Saccharopine	1.19E-11	−2.99
SLAD-2PE vs SHAD	YMD4529	191.01982	Diketogulonic acid	4.19E-06	3.71
		203.05261	d-Glucose	3.75E-06	2.35
		230.99023	Citric acid	0.0002	1.93
		229.03206	Homocitric acid	4.19E-06	1.90
		275.12493	Saccharopine	0.0001	1.87
		146.04597	l-Glutamic acid	2.34E-08	−2.13
		346.05593	2'-Deoxyguanosine 5'-monophosphate	3.75E-06	−1.96
		152.03186	Pyroglutamic acid_negative	1.20E-06	−1.63
		173.04228	d-Ribose	0.0149	−1.59
		186.01645	l-Glutamic acid	1.14E-05	−1.47
	YMD4537	191.01982	Diketogulonic acid	9.46E-09	3.50
		243.05279	l-Tryptophan	6.78E-10	2.56
		302.06623	Deoxyguanosine	3.83E-07	2.42
		132.03024	d-Aspartic acid	2.66E-08	2.34
		175.06127	2-Isopropylmalic acid	2.94E-07	2.07
		233.12436	l-Acetopine_negative	0.0020	−1.90
		346.05593	2'-Deoxyguanosine 5'-monophosphate	4.52E-08	−1.85
		131.0104	Glycerol	4.21E-05	−1.53
		131.01037	1_2-Benzoquinone	0.0009	−0.90
		173.04228	d-Ribose	3.66E-05	−0.83
	YMD4544	203.05261	d-Glucose	9.65E-06	3.47
		191.01982	Diketogulonic acid	6.17E-10	2.94
		147.11278	l-Lysine	5.43E-11	1.91
		243.06233	Uridine	1.24E-07	1.19
		281.05473	Aspartyl-hydroxyproline	8.09E-06	0.96
		142.05111	Trimethadione	3.10E-14	−4.02
		229.01211	5-Keto-d-gluconate	3.39E-13	−3.72
		214.04846	Glycerylphosphorylethanolamine	3.26E-14	−3.54
		306.07656	Glutathione	2.12E-14	−3.49
		233.12436	l-Acetopine_negative	5.92E-09	−3.35
SHAD vs SHAD-2PE	YMD4529	–	–	–	–
	YMD4537	191.01982	Diketogulonic acid	0.0230	0.78
		302.06623	Deoxyguanosine	8.79E-05	−1.74
		268.10407	Adenosine	0.0004	−1.42
		214.04846	Glycerylphosphorylethanolamine	1.03E-05	−1.28
		280.09196	Glycerophosphocholine	2.30E-06	−1.05
		133.09705	Ornithine	0.0126	−1.03
	YMD4544	191.01982	Diketogulonic acid	0.0206	0.92
		214.04743	hydroxyvalerylglycine	0.0052	0.75
		173.02092	Deoxyribose	0.0248	0.71
		147.06629	(R)-mevalonate	0.0206	0.48
		182.07874	5-Acetamidovalerate	0.0115	0.42
SLAD vs SLAD-2PE	YMD4529	–	–	–	–
	YMD4537	–	–	–	–
	YMD4544	346.05593	2'-Deoxyguanosine 5'-monophosphate	1.93E-06	1.62
		167.02305	*N*-Carbamoylsarcosine	1.12E-05	1.59
		171.01894	l-Asparagine	3.95E-06	1.51
		243.05279	l-Tryptophan	3.96E-07	1.50
		175.06127	2-Isopropylmalic acid	5.55E-06	1.49

Apart from changes to cellular networks and pathways resulting in filamentous outgrowth under nitrogen starvation, the differential expression of cellular signalling proteins, which might be the driving forces behind the phenotypic changes were also of interest. In strains YMD4529 and YMD4537, peptides matching a kinase involved in pseudohyphal growth (TPK2) were more abundant under nitrogen starvation (independent of 2-PE addition). In the individual strains, further differentially abundant peptides of kinases and phosphatases involved in pseudohyphal growth and filamentation were detected: in YMD4529, a peptide of the phosphatase PTP1, described as a negative regulator of filamentation was detected as being in lower abundance under nitrogen starvation conditions. In YMD4537, peptides belonging to the kinases SNF1 and KSP1 were detected as lower abundant under nitrogen starvation, while a peptide of STE20 was more abundant under these conditions (see Table S2). In line with the observed phenotype, no differential abundance of peptides from kinases or phosphatases involved in pseudohyphal or filamentous outgrowth were detected in YMD4544. The protein BCY1, a target of the SCH9 kinase (more abundant in YMD4537 SLAD versus SHAD), was also detected as differentially abundant in the YMD4529 and YMD4537 strains. Out of the five detected peptides of BCY1, four were more abundant under nitrogen starvation. However, in YMD4529 the peptide NIVLFPEPEESFSRPQSAQSQSR of BCY1 was detected as having lower abundance under nitrogen starvation in the conditions SLAD versus SHAD (adj. *P*-value = .032) and SLAD-2-PE versus SHAD (adj. *P*-value = .051) and potentially in SLAD-2-PE versus SHAD-2-PE (adj. *P*-value = .090, see Table S1). This discrepancy can be interpreted as an addition of a posttranslational modification on an amino acid within this sequence a potential explanation would be the phosphorylation of a serine in this sequence as it contains a RPQS motif, which is a consensus motif of theSCH9 kinase.

Further, ubiquitylation-related proteins, as another class of cell-signalling proteins, were investigated. Apart from the E1 enzyme UBA1 mentioned above, peptides of RSP5 (an essential NEDD4 E3 ligase involved in a.o. ribosome stability) were detected as more abundant under nitrogen starvation conditions (with and without 2-PE addition) in the yeasts YMD4529 and YMD4537, but not in YMD4544 (see Tables S1–S3).

### Metabolomics

**Figure 3.**
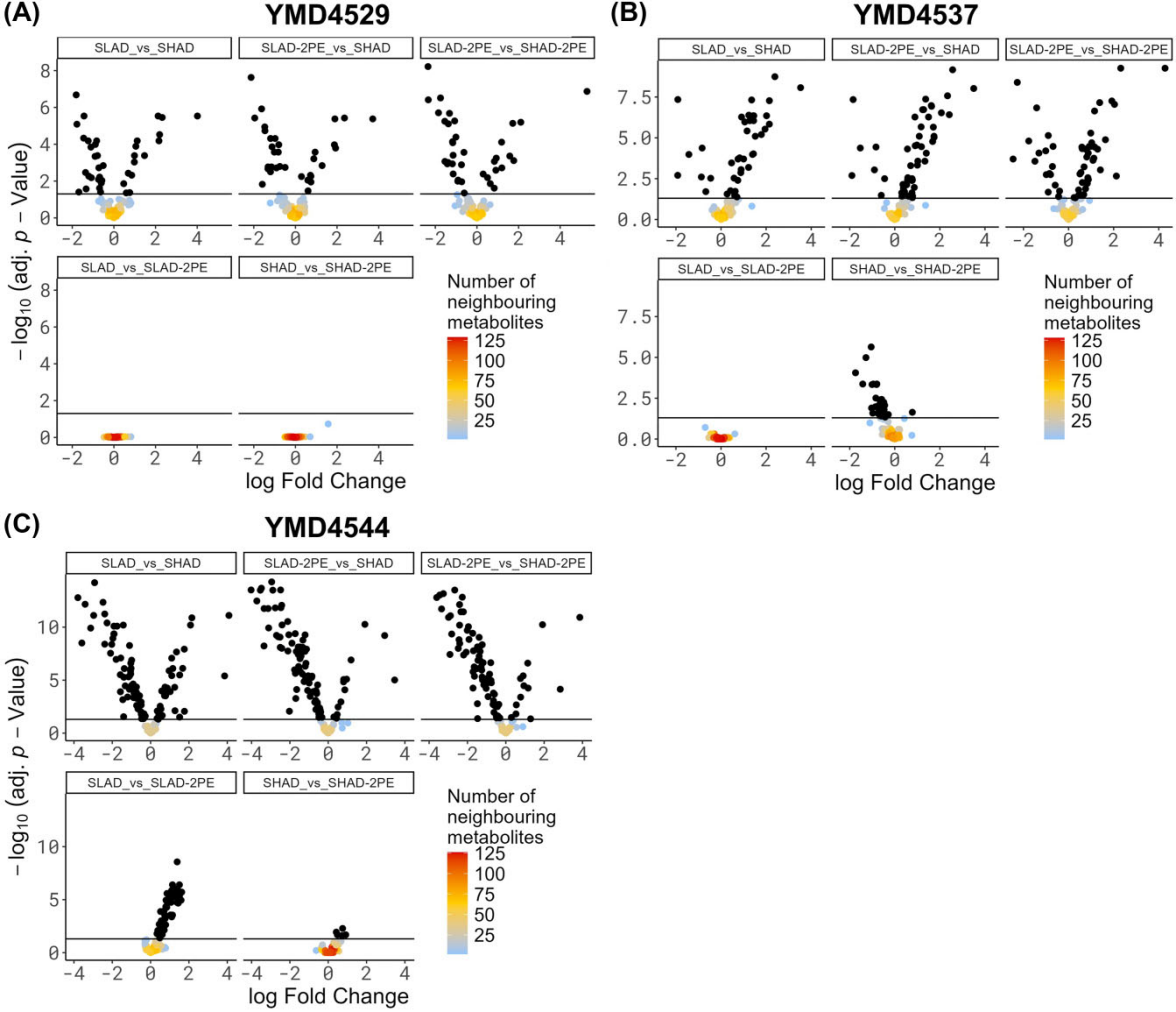
Volcano plots of differentially abundant metabolites. (A) YMD4529, (B) YMD4537, and (C) YMD4544. Horizontal lines in each subplot indicate a adj. *P*-value cut-off of 5%, differentially abundant metabolites are shown in black. Log fold change is relative to the first named condition in the subgraphs, i.e. a negative log fold change indicates a higher abundance of the metabolite in the first named condition.

Simple differential proteome analysis fails to provide insight into the technical challenge, that changes in the level of abundance of proteins does not necessarily relate to a change in the concentration of the catalytic products of proteins with enzymatic functions. For this reason, an analysis was carried out to investigate if the changes to the proteome were also (partially) reflected in the metabolome. Metabolites from the individual yeast strains were extracted, using yeast cells from the same plates as for the proteomics analysis. In total, 335 unique metabolites were identified and quantified, of which 143 compounds could be mapped to a KEGG compound number in relation to *S. cerevisiae*. The influence of nitrogen starvation, by comparison of SLAD and SHAD, showed that 15 compounds were more abundant in SLAD (YMD4529), while 23 compounds were less abundant. In YMD4537 34 compounds were detected as higher and seven as lower in abundance, while in YMD4544 31 higher and 68 compounds lower abundance (adj. *P*-value ≤ .05, see Fig. 3 and for the top 10 regulated metabolites see Table 3, all metabolites in Tables S5–S7). The identity of these compounds mostly overlaps between YMD4529 and YMD4537 (lower abundance in SLAD: 6 out of 23 in YMD4529 and 6 out of 7 in YMD4537 and higher abundance in SLAD: 11 out 15 in YMD4529 and 11 out of 34 in YMD4537). The inclusion of YMD4544 (which lacks a detectable phenotype change) into this comparison results in the overlap of 16 compounds between the three strains. However, when taking the direction of the abundance change into account, only three higher abundant compounds overlap (diketogulonic acid, uridine, and aspartyl-hydroxyproline), and two lower abundant compounds (glutathione and 2'-deoxyguanosine 5'-monophosphate). As in the proteome analysis, the yeast YMD4529 did not show differentially abundant metabolites in response to 2-PE (see Fig. 3). Surprisingly, this was different for YMD4537 and YMD4544. In YMD4537, SHAD versus SHAD-2-PE resulted in 29 metabolites, which were all more abundant under 2-PE addition, except for diketogulonic acid (logFC: 0.77, adj. *P*-value = .027) (see Fig. 3). In contrast, in YMD4544 the majority of metabolites were lower abundant after 2-PE addition, under both SLAD (56 compounds with adj. *P*-value ≤ .05) and SHAD conditions (5 compounds with adj. *P*-value ≤ .05). Regarding YMD4537, diketogulonic acid was also detected as lower abundant under SHAD-2-PE conditions in YMD4544 (logFC: 0.92, adj. *P*-value = .021). A further compound, (R)-mevalonate, overlapped between the SHAD versus SHAD-2-PE condition in YMD4537 and YMD4544, with higher abundance observed in YMD4537 under 2-PE addition (logFC: −0.65, adj. *P*-value = .003), while in YMD4544 this compound was lower abundant (logFC: 0.48, adj. *P*-value = .021).

Compounds which exhibited an adjusted *P*-value ≤ .05 were further analysed for enrichment of specific classes (compound, enzyme, module, and reaction) using FELLA (Picart-Armada et al. 2018). For the comparison of SLAD versus SHAD, involvement of amino acid metabolism was detected in YMD4529 and YMD4537. Additionally, in YMD4537 the SLAD versus SLAD-2-PE comparison revealed the reaction ‘l-cysteine:[enzyme]-cysteine sulfurtransferase’ (KEGG ID: R07460) to be significant (p.score = 0.044) (see Fig. S3 and Table S8). No metabolomic networks were detected for YMD4544, using a p.score cut-off of 0.05.

### Lipidomics

**Figure 4.**
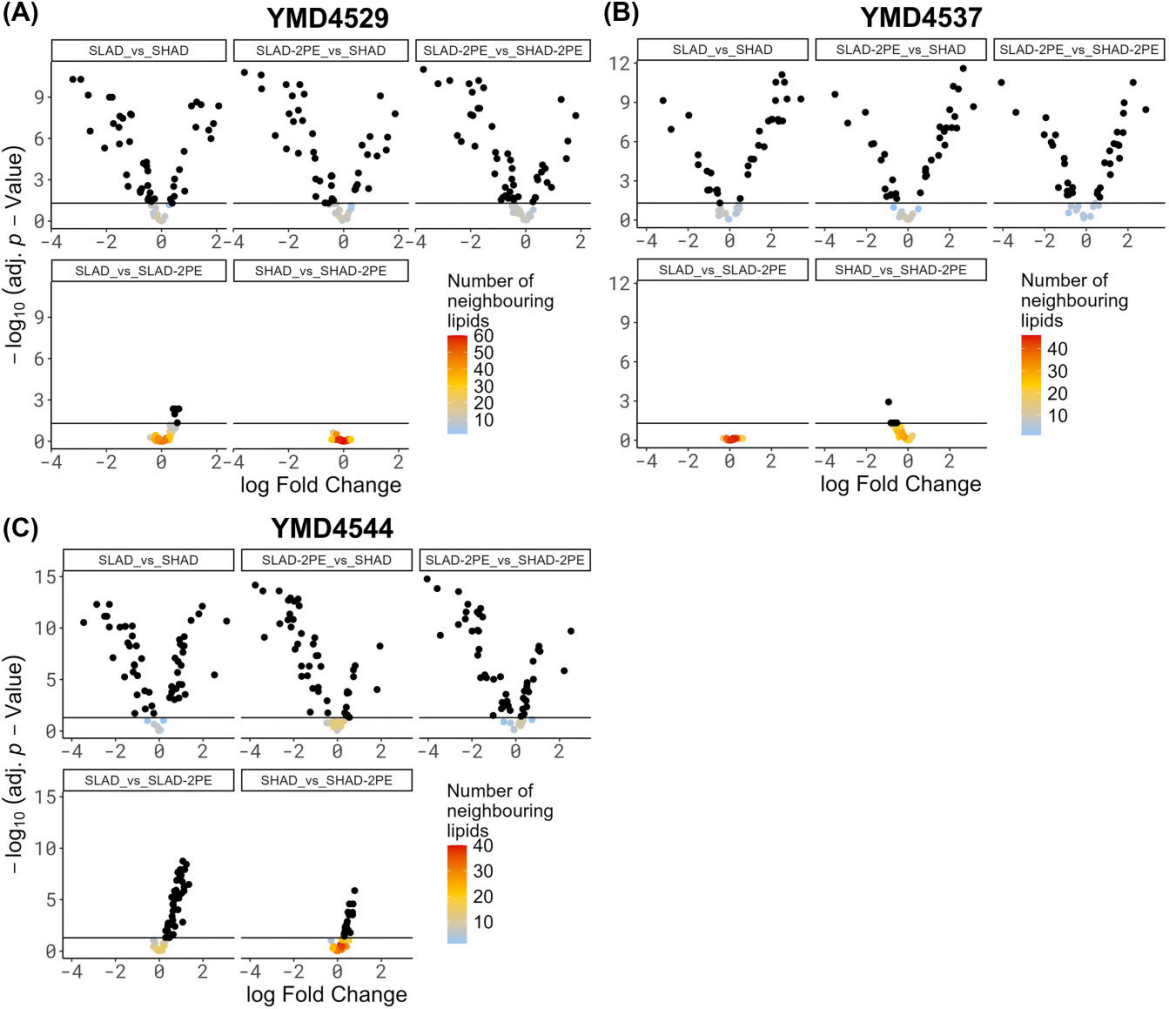
Volcano plots of differentially abundant lipids. (A) YMD4529, (B) YMD4537, and (C) YMD4544. Horizontal lines in each subplot indicate a adj. *P*-value cut-off of 5%, differentially abundant metabolites are shown in black. Log fold change is relative to the first named condition in the subgraphs, i.e. a negative log fold change indicates a higher abundance of the lipid in the first named condition.

**Figure 5.**
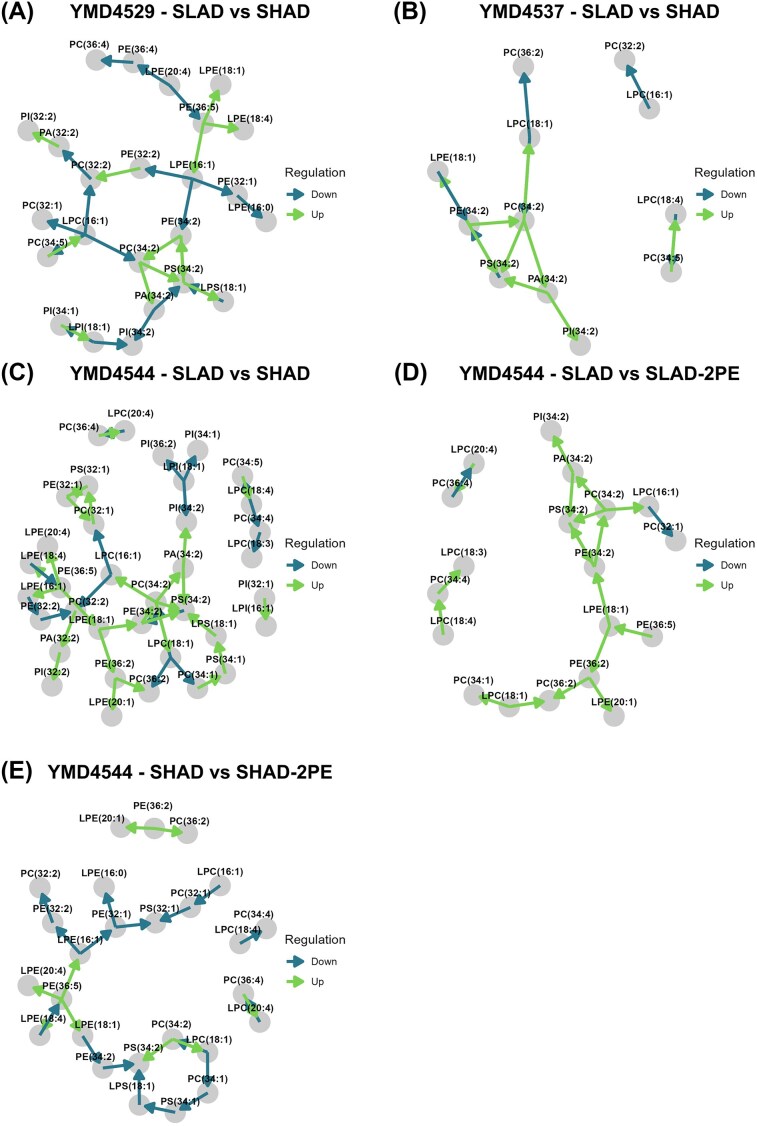
Identified pathways of differentially abundant lipids between specified conditions. SLAD versus SHAD in (A) YMD4529, (B) YMD4537, and (C) YMD4544. (D) and (E) show regulatory networks in YMD4544 under addition of 2-PE in SLAD and SHAD, respectively. Arrows indicate the direction of the pathways (precursor to product) with a reaction chain z-score > 1.64 (*P* ≤ .05) as assigned by bioPAN.

As there is potential involvement of the cell membrane in filamentous outgrowth, the differential lipidome of YMD4529, YMD4537, and YMD4544 in response to nitrogen starvation and 2-PE addition was investigated. Mass spectrometry analysis identified 89 unique lipids, which were analysed for differential abundance between the experimental conditions. Nitrogen starvation, (SLAD versus SHAD) resulted in 46 lipids to be detected as differentially abundant in YMD4529, 33 lipids in YMD4537, and 53 in YMD4544 (adj. *P*-value ≤ .05). Interestingly, changes in lipid abundance in reaction to 2-PE addition could be detected readily, as in contrast to the proteomics results, although the results were inconsistent between the yeast strains. In YMD4529, five lipids were detected as having lower abundance under 2-PE addition in SLAD versus SLAD-2-PE, while in YMD4537, six lipids were higher in abundance after 2-PE addition. In stark contrast stands YMD4544, in which 43 lipids were detected as differentially abundant in SLAD versus SLAD-2-PE while further 18 lipids were differential abundant in SHAD versus SHAD-2-PE. In both conditions, all lipids were observed to have a lower abundance after 2-PE addition (adj. *P*-value ≤ .05) (see Fig. 4, and for the top 10 regulated lipids see Table 4, all lipids in Tables S9–S12). The differentially abundant lipids were further analysed for involvement in known lipid reaction pathways and the specific genes/proteins related to these, using bioPAN (Ni and Fedorova 2020, Gaud et al. 2021) (see Fig. 5 and Fig. S4). As bioPAN utilizes human data, the resulting human genes were mapped onto yeast genes, and these genes were checked to determine if they also were detected as differentially abundant in the proteomics data. From this comparison, the protein PLB1 was detected as being involved in known lipid reaction pathways and two unique peptides of this protein were found to be more abundant (adj. *P*-value ≤ .05) under nitrogen starvation in the conditions SLAD versus SHAD and SLAD-2-PE versus SHAD in YMD4537.

**Table 4.** Top five differentially abundant lipids under all studied conditions. The logFC gives the change in abundance of the identified lipid (adj. *P*-value ≤ .05), with a positive logFC indicating a higher abundant peptide in the first named condition in the column ‘Comparison’. The m/z column shows the experimentally observed mass-to-charge ratio of the lipid.

Comparison	Yeast	m/z	Lipid name	Formula	adj. *P-*value	logFC
SLAD vs SHAD	YMD4529	597.30397	LPI 18:1	C27H51O12P	4.36E-09	2.07
		861.54822	PI 36:2	C45H83O13P	8.33E-08	1.88
		522.28314	LPS 18:1	C24H46NO9P	1.03E-06	1.79
		863.56381	PI 36:1	C45H85O13P	2.47E-07	1.71
		835.53275	PI 34:1	C43H81O13P	3.53E-09	1.43
		754.53665	PC 34:4	C42H76NO8P	5.14E-11	−3.22
		917.54499	SQDG 42:8	C51H82O12S	5.14E-11	−2.93
		782.56789	PC 36:4	C44H80NO8P	7.14E-10	−2.66
		726.50484	PC 32:4	C40H72NO8P	2.93E-07	−2.59
		452.27819	LPE 16:0	C21H44NO7P	4.99E-06	−2.06
	YMD4537	746.53297	PS O-34:1	C40H78NO9P	5.46E-10	3.41
		544.33783	LPC 20:4	C28H50NO7P	5.46E-10	2.75
		478.29349	LPE 18:1	C23H46NO7P	2.86E-11	2.63
		774.56416	PS O-36:1	C42H82NO9P	2.60E-08	2.53
		863.56381	PI 36:1	C45H85O13P	7.54E-12	2.50
		754.53665	PC 34:4	C42H76NO8P	7.15E-10	−3.20
		782.56789	PC 36:4	C44H80NO8P	1.14E-07	−2.81
		917.54499	SQDG 42:8	C51H82O12S	9.63E-09	−1.97
		752.52108	PC 34:5	C42H74NO8P	1.02E-05	−1.52
		560.43096	Cer 34:6;O4	C34H57NO5	5.85E-05	−1.51
	YMD4544	597.30397	LPI 18:1	C27H51O12P	2.10E-11	3.08
		522.28314	LPS 18:1	C24H46NO9P	3.51E-06	2.52
		863.56381	PI 36:1	C45H85O13P	7.55E-13	1.97
		746.53297	PS O-34:1	C40H78NO9P	4.21E-12	1.81
		760.5121	PS 34:1	C40H76NO10P	1.80E-11	1.45
		747.51696	PG 34:1	C40H77O10P	2.88E-11	−3.46
		754.53665	PC 34:4	C42H76NO8P	5.02E-13	−2.86
		726.50484	PC 32:4	C40H72NO8P	7.19E-12	−2.50
		782.56789	PC 36:4	C44H80NO8P	7.19E-12	−2.39
		452.27819	LPE 16:0	C21H44NO7P	7.87E-11	−2.28
SLAD-2PE vs SHAD-2PE	YMD4529	597.30397	LPI 18:1	C27H51O12P	2.18E-08	1.81
		861.54822	PI 36:2	C45H83O13P	1.55E-06	1.53
		522.28314	LPS 18:1	C24H46NO9P	2.92E-05	1.47
		746.53297	PS O-34:1	C40H78NO9P	1.48E-09	1.28
		788.54319	PS 36:1	C42H80NO10P	0.0035	0.93
		754.53665	PC 34:4	C42H76NO8P	9.74E-12	−3.71
		782.56789	PC 36:4	C44H80NO8P	1.06E-10	−3.18
		917.54499	SQDG 42:8	C51H82O12S	6.23E-11	−2.76
		726.50484	PC 32:4	C40H72NO8P	6.13E-07	−2.48
		452.27819	LPE 16:0	C21H44NO7P	1.65E-06	−2.34
	YMD4537	746.53297	PS O-34:1	C40H78NO9P	3.55E-09	2.86
		863.56381	PI 36:1	C45H85O13P	2.97E-11	2.25
		833.51698	PI 34:2	C43H79O13P	1.06E-09	1.82
		522.35584	LPC 18:1	C26H52NO7P	6.67E-09	1.79
		502.29083	LPE 20:4	C25H44NO7P	2.03E-07	1.76
		754.53665	PC 34:4	C42H76NO8P	2.97E-11	−4.07
		782.56789	PC 36:4	C44H80NO8P	5.72E-09	−3.38
		752.52108	PC 34:5	C42H74NO8P	2.95E-07	−2.02
		917.54499	SQDG 42:8	C51H82O12S	1.46E-08	−1.94
		780.55229	PC 34:2	C42H80NO8P	1.11E-06	−1.68
	YMD4544	597.30397	LPI 18:1	C27H51O12P	1.98E-10	2.52
		522.28314	LPS 18:1	C24H46NO9P	1.41E-06	2.22
		502.29083	LPE 20:4	C25H44NO7P	1.83E-08	1.11
		863.56381	PI 36:1	C45H85O13P	5.71E-09	1.06
		746.53297	PS O-34:1	C40H78NO9P	1.27E-08	1.03
		754.53665	PC 34:4	C42H76NO8P	1.70E-15	−4.05
		782.56789	PC 36:4	C44H80NO8P	1.47E-14	−3.59
		747.51696	PG 34:1	C40H77O10P	5.04E-10	−3.44
		726.50484	PC 32:4	C40H72NO8P	4.55E-11	−2.62
		732.55433	PC 32:1	C40H78NO8P	2.87E-14	−2.61
SLAD-2PE vs SHAD	YMD4529	597.30397	LPI 18:1	C27H51O12P	1.62E-08	1.87
		861.54822	PI 36:2	C45H83O13P	8.02E-07	1.59
		522.28314	LPS 18:1	C24H46NO9P	6.95E-06	1.54
		746.53297	PS O-34:1	C40H78NO9P	8.13E-10	1.33
		863.56381	PI 36:1	C45H85O13P	1.87E-05	1.21
		754.53665	PC 34:4	C42H76NO8P	1.61E-11	−3.60
		917.54499	SQDG 42:8	C51H82O12S	2.48E-11	−2.99
		782.56789	PC 36:4	C44H80NO8P	2.51E-10	−2.98
		726.50484	PC 32:4	C40H72NO8P	6.16E-07	−2.48
		780.55229	PC 34:2	C42H80NO8P	1.23E-10	−2.09
	YMD4537	746.53297	PS O-34:1	C40H78NO9P	2.11E-09	3.14
		863.56381	PI 36:1	C45H85O13P	2.50E-12	2.65
		478.29349	LPE 18:1	C23H46NO7P	9.47E-11	2.42
		774.56416	PS O-36:1	C42H82NO9P	9.15E-08	2.34
		544.33783	LPC 20:4	C28H50NO7P	1.20E-08	2.24
		754.53665	PC 34:4	C42H76NO8P	2.40E-10	−3.51
		782.56789	PC 36:4	C44H80NO8P	3.77E-08	−2.89
		917.54499	SQDG 42:8	C51H82O12S	5.70E-09	−2.05
		752.52108	PC 34:5	C42H74NO8P	1.56E-06	−1.75
		560.43096	Cer 34:6;O4	C34H57NO5	1.41E-06	−1.65
	YMD4544	597.30397	LPI 18:1	C27H51O12P	5.61E-09	1.94
		522.28314	LPS 18:1	C24H46NO9P	9.31E-05	1.81
		746.53297	PS O-34:1	C40H78NO9P	4.50E-07	0.81
		502.29083	LPE 20:4	C25H44NO7P	5.37E-06	0.73
		863.56381	PI 36:1	C45H85O13P	1.14E-06	0.73
		754.53665	PC 34:4	C42H76NO8P	6.75E-15	−3.76
		782.56789	PC 36:4	C44H80NO8P	2.49E-14	−3.41
		747.51696	PG 34:1	C40H77O10P	7.93E-10	−3.34
		732.55433	PC 32:1	C40H78NO8P	2.49E-14	−2.67
		726.50484	PC 32:4	C40H72NO8P	3.78E-11	−2.64
SLAD vs SLAD-2PE	YMD4529	758.57058	PC 34:2	C42H80NO8P	0.0045	0.63
		730.53897	PC 32:2	C40H76NO8P	0.0455	0.57
		714.50683	PE 34:2	C39H74NO8P	0.0045	0.50
		835.53275	PI 34:1	C43H81O13P	0.0105	0.48
		807.50146	PI 32:1	C41H77O13P	0.0045	0.42
	YMD4537	–	–	–	–	–
	YMD4544	889.51371	SQDG 40:8	C49H78O12S	3.30E-07	1.35
		863.56381	PI 36:1	C45H85O13P	3.69E-09	1.24
		802.59537	PS O-38:1	C44H86NO9P	1.18E-08	1.16
		774.56416	PS O-36:1	C42H82NO9P	1.18E-08	1.14
		597.30397	LPI 18:1	C27H51O12P	5.76E-07	1.14
SHAD vs SHAD-2PE	YMD4529	–	–	–	–	–
		478.29349	LPE 18:1	C23H46NO7P	0.0012	−0.93
		802.59537	PS O-38:1	C44H86NO9P	0.0469	−0.82
		776.57974	PS O-36:0	C42H84NO9P	0.0469	−0.70
		544.33783	LPC 20:4	C28H50NO7P	0.0469	−0.68
		758.57058	PC 34:2	C42H80NO8P	0.0469	−0.59
	YMD4544	833.51698	PI 34:2	C43H79O13P	1.31E-06	0.78
		805.48567	PI 32:2	C41H75O13P	2.57E-05	0.71
		522.35584	LPC 18:1	C26H52NO7P	0.0003	0.69
		861.54822	PI 36:2	C45H83O13P	0.0002	0.69
		597.30397	LPI 18:1	C27H51O12P	0.0159	0.58

## Discussion

This investigation represents a nuanced exploration of the biochemical and phenotypic responses of industrial brewing strains of *S. cerevisiae* to QS mechanisms mediated by the aromatic alcohol 2-PE, particularly under varying nitrogen conditions. By focusing on three industrial brewing strains, the research uncovers the intricate interplay between cellular signalling, environmental cues, and strain-specific genetic regulation, offering insight into the understanding of yeast behavior within the densely populated environments, like those observed in commercial brewing fermentations.

A key revelation of this investigation is the substantial strain-specific divergence in responses to 2-PE. The morphogenic transitions observed in strains YMD4529 and YMD4537 under nitrogen-depleted conditions starkly contrast with the unresponsive phenotype of strain YMD4544, despite the uniform experimental conditions. This variability suggests that the QS pathways and their downstream regulatory networks are neither universally conserved nor uniformly expressed among industrial *S. cerevisiae* strains—which is consistent with the previous conclusions of Lenhart et al. (2019) and Britton et al. (2023), who examined environmental, industrial, clinical, and industrially relevant strains. Such findings emphasize the need to reassess assumptions of homogeneity across brewing strains, as genetic divergence, domestication pressures, or environmental adaptation may have selectively attenuated or enhanced QS capabilities in individual strains.

Proteomic analysis revealed the pronounced effects of nitrogen limitation on ribosomal proteins and associated pathways, suggesting a robust cellular reprogramming under stress-induced nutrient starvation. However, the absence of a proteomic transition to 2-PE across all strains introduces intriguing questions regarding its functional role as a QS molecule in *S. cerevisiae*. This observation suggests that 2-PE’s influence may operate more subtly, potentially modulating specific biochemical pathways without eliciting broad proteomic shifts. On the other hand, lipidomic and metabolomic analyses revealed significant and more readily apparent changes, indicating that 2-PE may primarily exert its effects by reshaping membrane composition and metabolic fluxes. These alterations, though less dramatic than proteomic responses, could play critical roles in the adaptive strategies employed by yeast in high-density, resource-limited fermentations.

The practical implications of these findings are multifaceted, particularly for the brewing industry, where yeast physiology directly impacts product quality. The capacity of 2-PE to modulate lipid profiles and metabolic pathways could be strategically leveraged to improve fermentation efficiency. For instance, yeast strains exhibiting clear QS phenotypes, such as YMD4529 and YMD4537, may be particularly advantageous in brewing scenarios requiring robust metabolic adaptability and cooperative behavior. This includes fermentations exposing yeast to elevated stress conditions, such as the osmotic stress associated with high-gravity brewing, or those involved in a population of mixed cultures. Conversely, strains like YMD4544, which remain unresponsive to 2-PE, might necessitate alternative approaches beyond controlling cell density and pitching rate to elicit desired metabolic outcomes. At a minimum, this observed variability highlights the importance of strain-specific selection and tailored process optimization in achieving consistent and high-quality brewing results. However, further investigation would be required prior to utilizing aromatic alcohols for such applications at an industrial brewing scale.

Beyond its immediate industrial relevance, the study provokes broader questions about the evolutionary dynamics and ecological roles of QS in domesticated yeasts. The strain-specificity observed in response to 2-PE suggests that domestication, environmental constraints, and fermentation-specific pressures may have sculpted the QS repertoire of brewing yeasts. It is conceivable that certain strains have retained ancestral signalling mechanisms, while others have been artificially selected with adapted or attenuated pathways to better suit the industrial brewing environment. Investigating these evolutionary trajectories could uncover valuable insights into microbial adaptation, not only enriching our understanding of yeast biology but also offering clues to improve the resilience and performance of industrial strains.

The potential contributions of 2-PE extend into fundamental biology, particularly its role in mediating lipidomic and metabolomic shifts. Lipids are crucial to cellular integrity and function, particularly under the physical and chemical stresses of fermentation. The findings here suggest that the QS molecule 2-PE may act as a modulator of membrane dynamics, influencing transport processes, nutrient assimilation, and stress tolerance in select brewing strains of *S. cerevisiae*. This is not too dissimilar from the observed effects of QS molecules on Ascomycota. A recent example in *C. albicans* and *C. auris* demonstrated that 3-oxo-C12-homoserine lactone, a bacterial QS molecule, influences the upregulation and downregulation of genes involved in fatty acid and sterol biosynthesis, stress tolerance, and transmembrane transport, among other processes (Kovács et al. 2025). These subtle but critical shifts could underpin broader metabolic changes, enhancing yeast adaptability to fluctuating environmental conditions. Similarly, the metabolomic alterations observed—especially those related to amino acid and carbohydrate metabolism—further underscore the role of 2-PE as a key influencer of metabolic strategy. Together, these findings suggest that QS via 2-PE operates as a finely tuned mechanism, subtly optimizing yeast function within the constraints of high-density fermentative environments.

This study deepens the understanding of QS dynamics in industrial *S. cerevisiae*, uncovering a complex and highly specific interaction between signalling molecules, environmental conditions, and strain-dependent genetic architecture. The findings underscore the importance of considering individual strain characteristics in the context of industrial applications, particularly in brewing, where yeast behavior is integral to product quality. By unravelling the nuanced roles of 2-PE and other QS molecules, this research not only advances the scientific discourse on microbial communication but also lays the groundwork for more deliberate and innovative fermentation strategies. The insights gained here hold promise for both enhancing beer production and broadening our comprehension of microbial sociobiology within applied and natural contexts. These differences may hold profound implications for their industrial utility, particularly in optimizing fermentation processes that rely on cooperative yeast behaviors.

## Supplementary Material

foaf036_Supplemental_Files
