# Supplementary material for: Quorum sensing in Saccharomyces cerevisiae brewing strains: effects of 2-phenylethanol on proteomic, lipidomic, and metabolomic profile
Source: FEMS Yeast Res. 2025 Jul 7;25:foaf036. doi: 10.1093/femsyr/foaf036 (PMC12254953; doi:10.1093/femsyr/foaf036)

YMD4529-SLAD_vs_SHAD_down

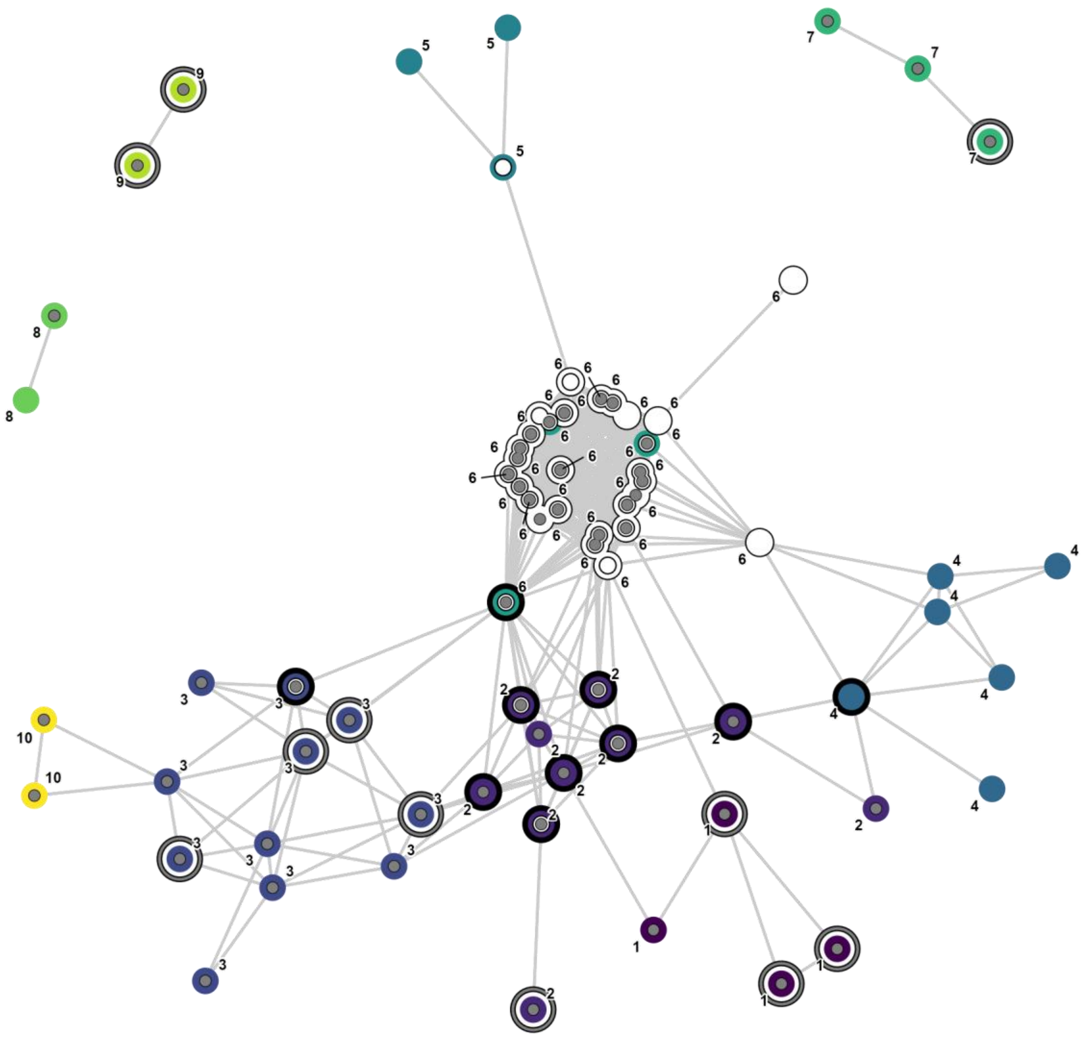

YMD4529-SLAD_vs_SHAD_down

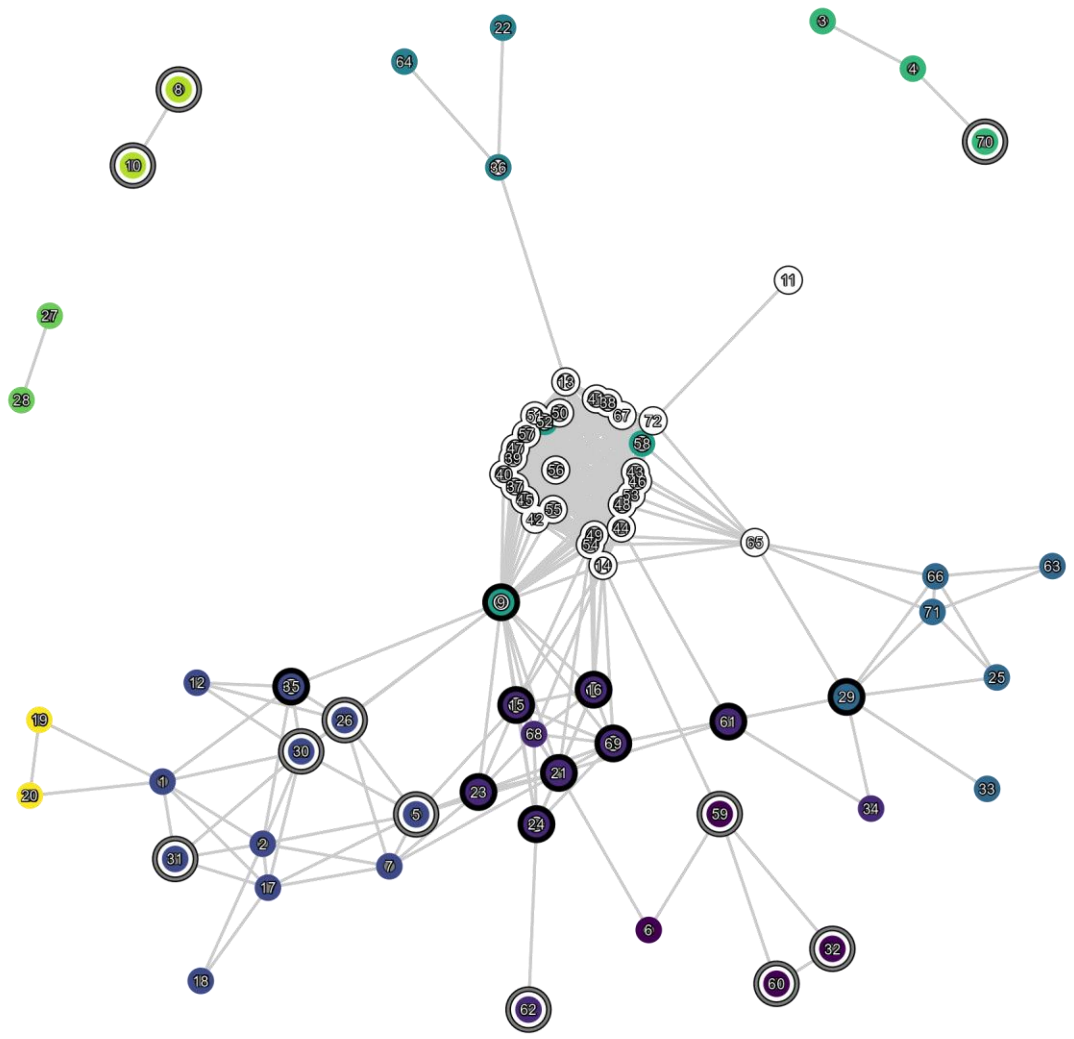

YMD4529-SLAD_vs_SHAD_up

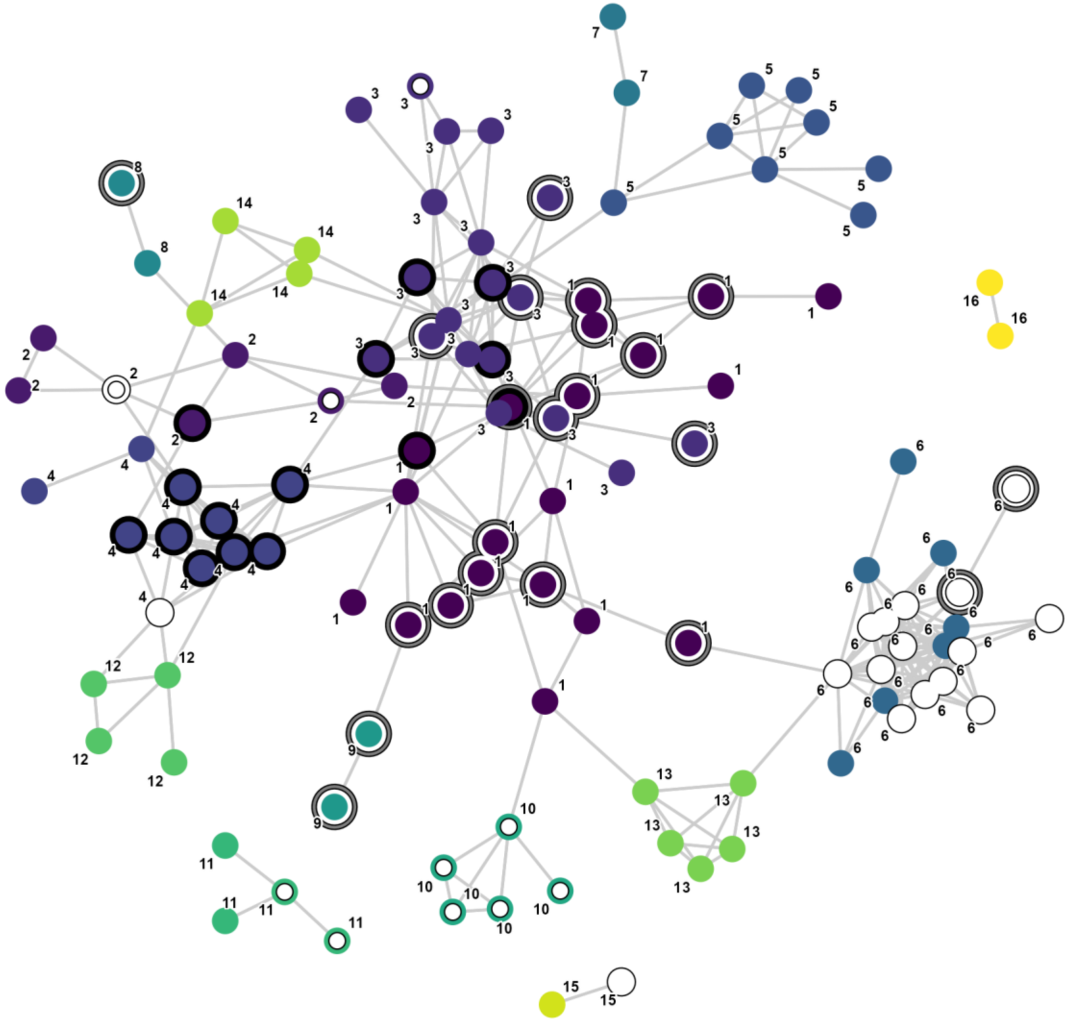

YMD4529-SLAD_vs_SHAD_up

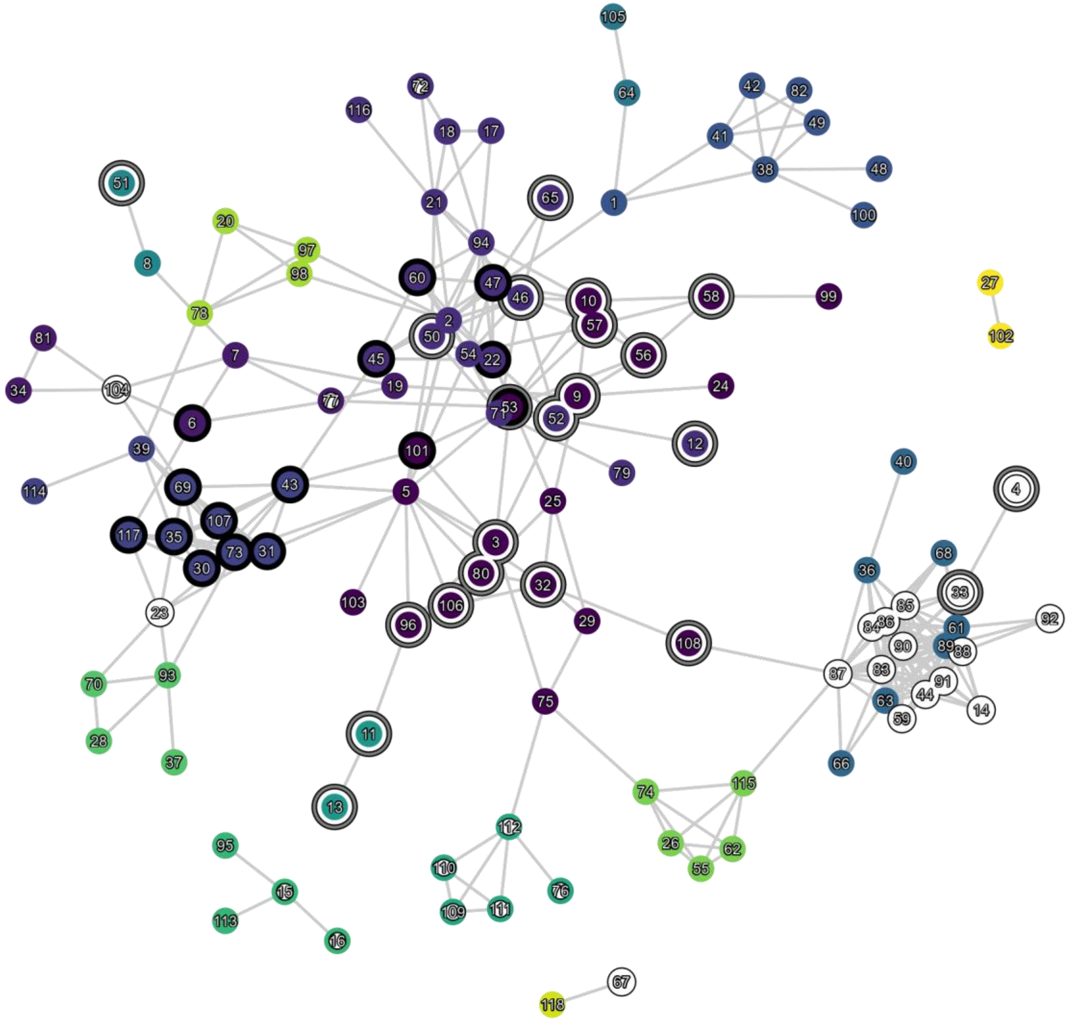

YMD4529-SLAD-2PE_vs_SHAD_down

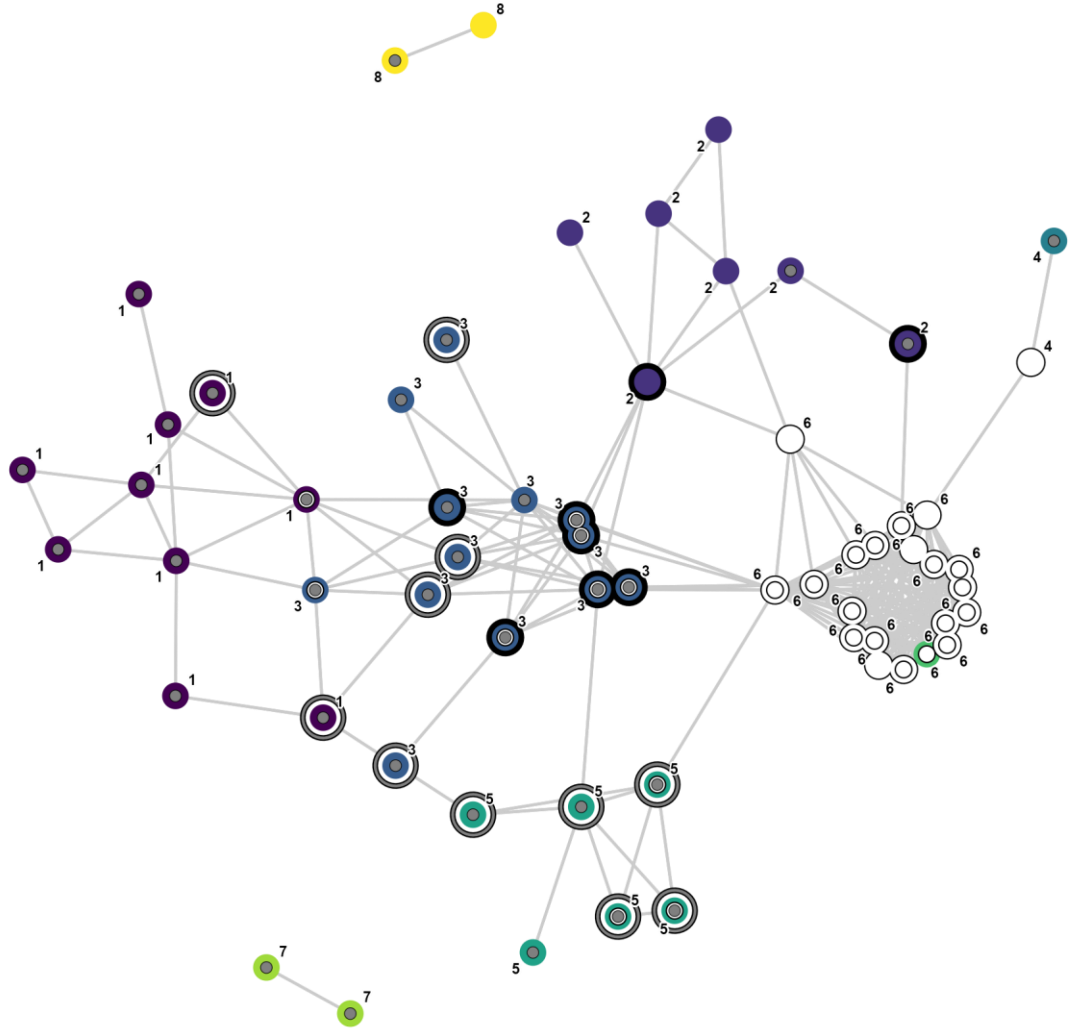

YMD4529-SLAD-2PE_vs_SHAD_down

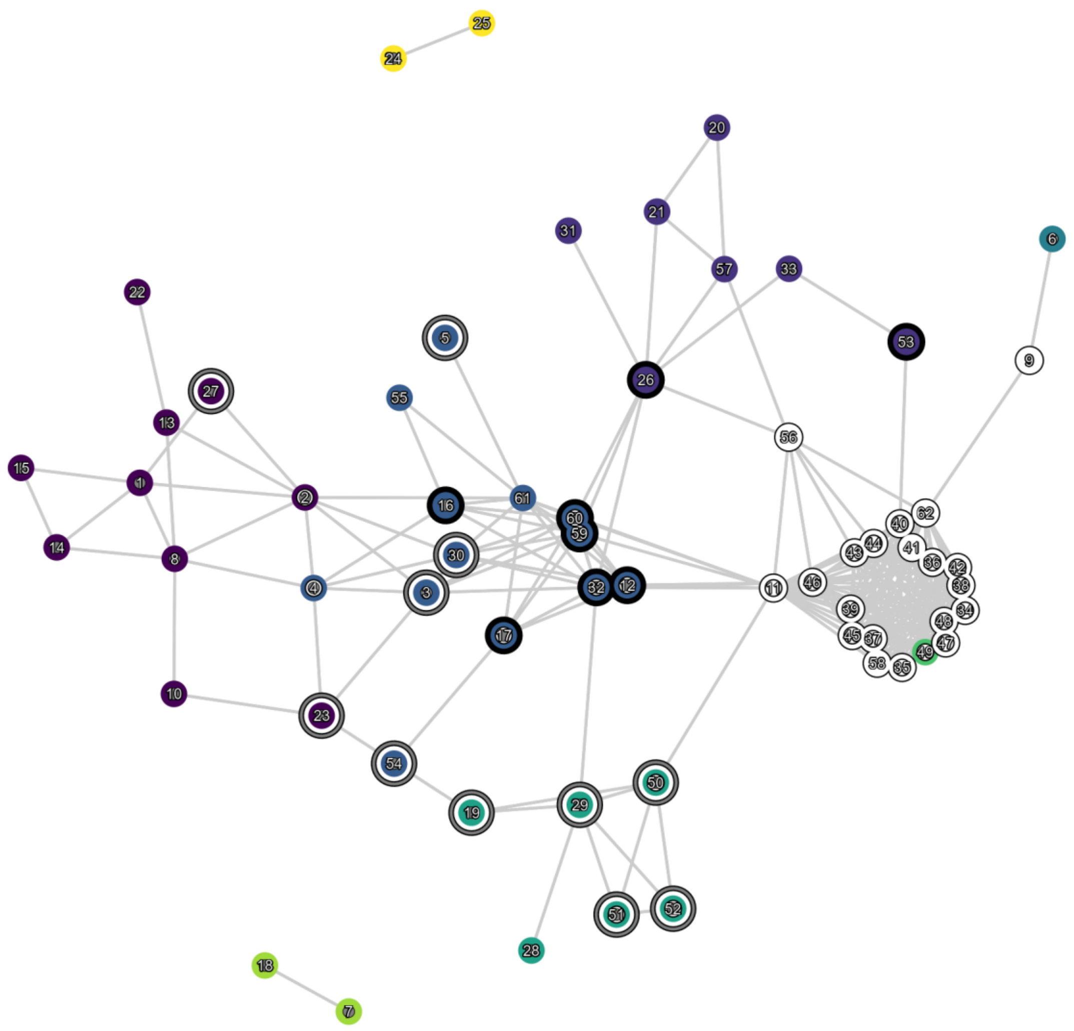

YMD4529-SLAD-2PE_vs_SHAD_up

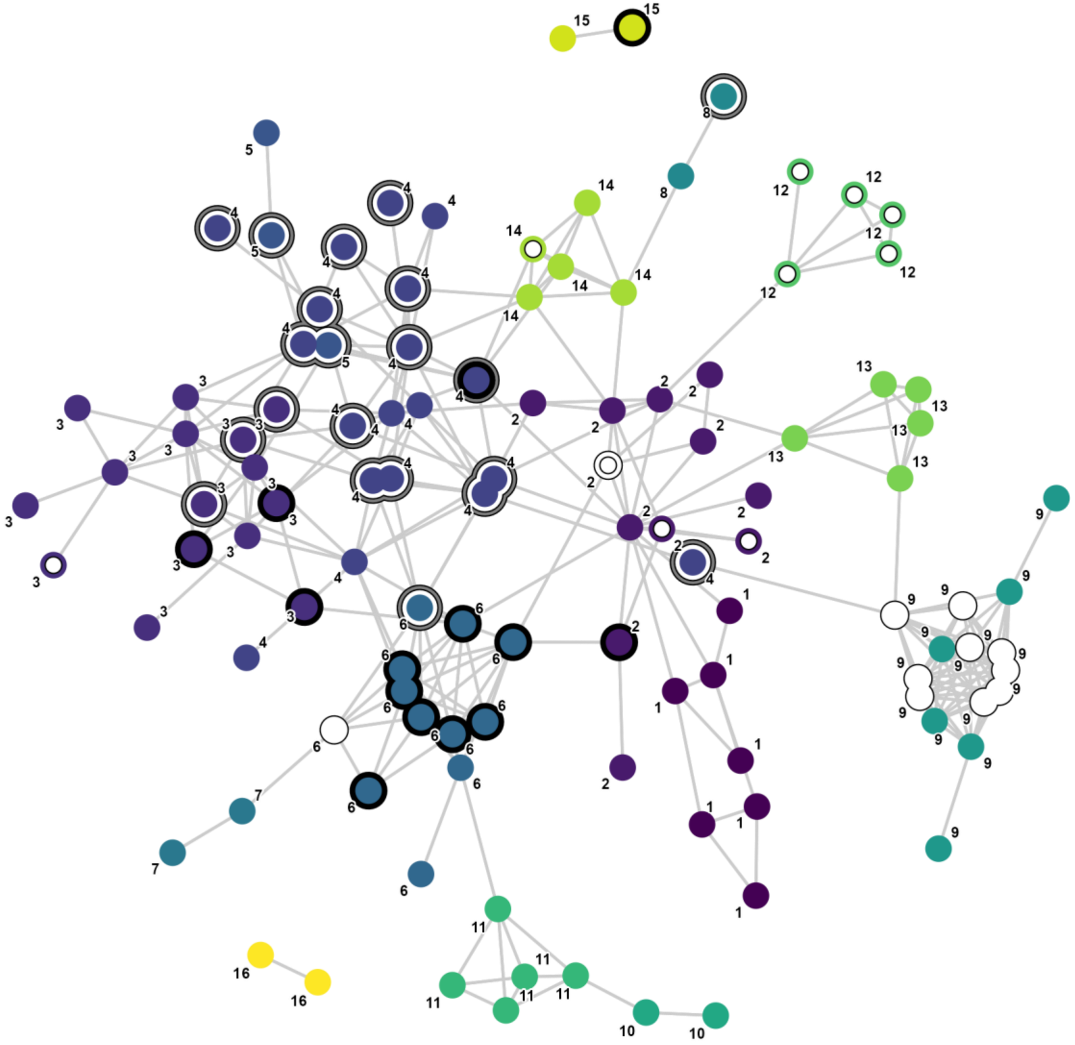

YMD4529-SLAD-2PE_vs_SHAD_up

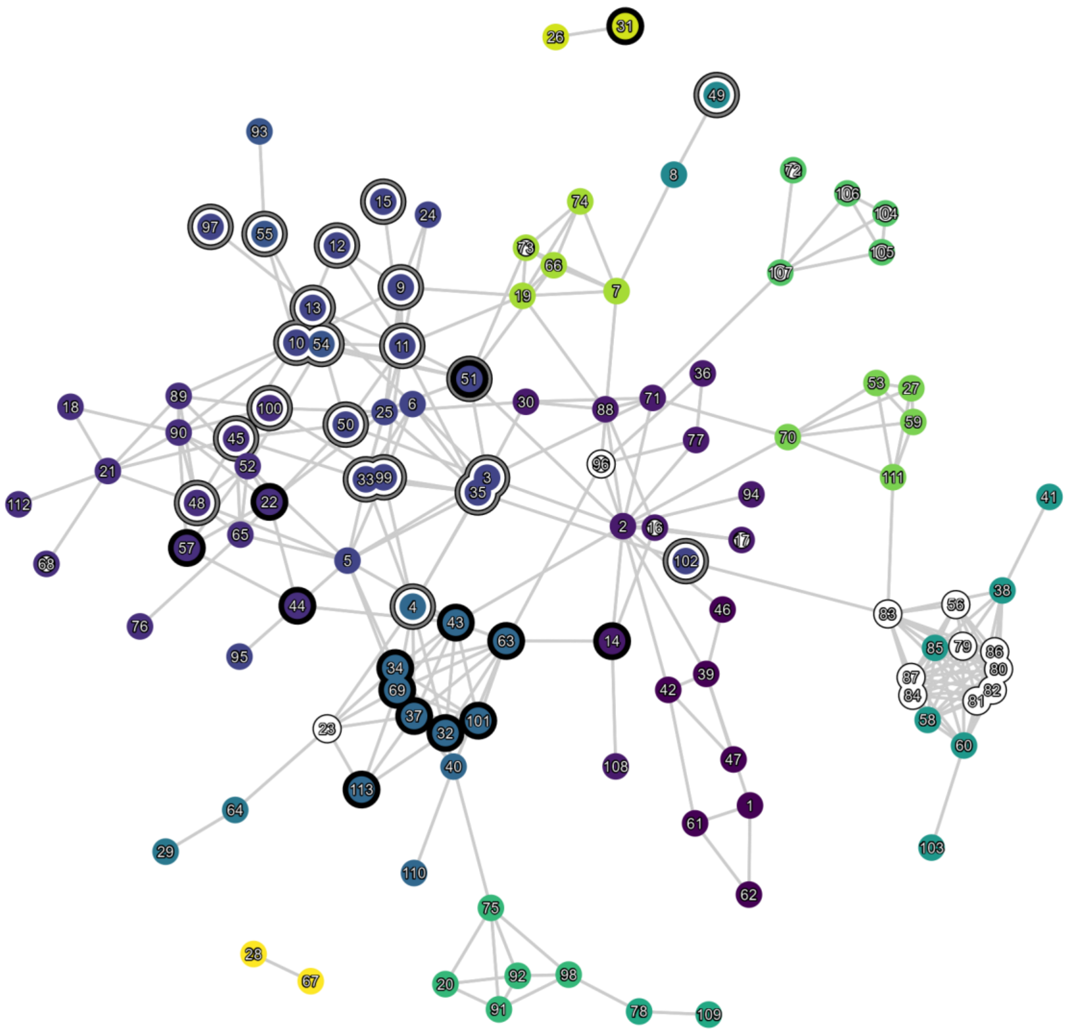

YMD4529-SLAD-2PE_vs_SHAD-2PE_down

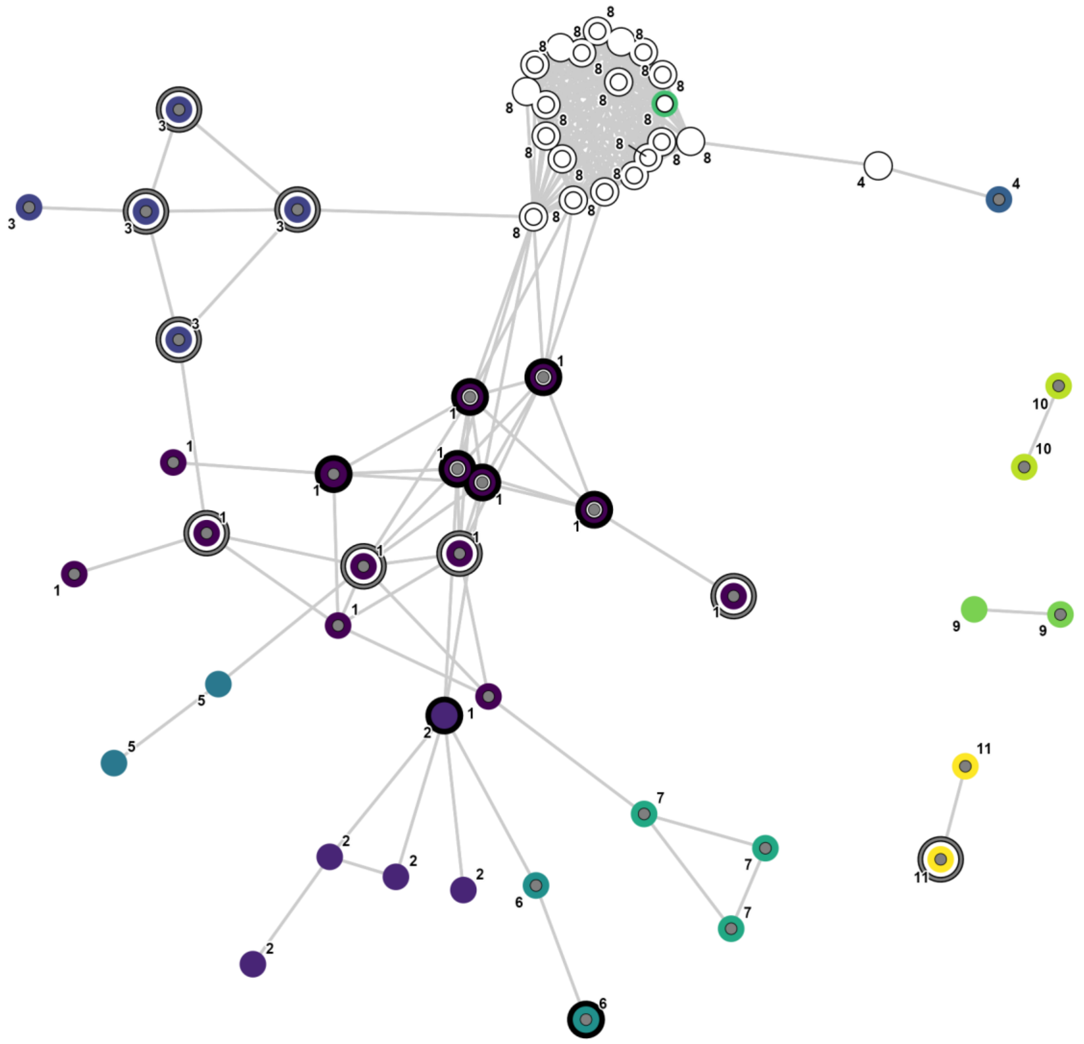

YMD4529-SLAD-2PE_vs_SHAD-2PE_down

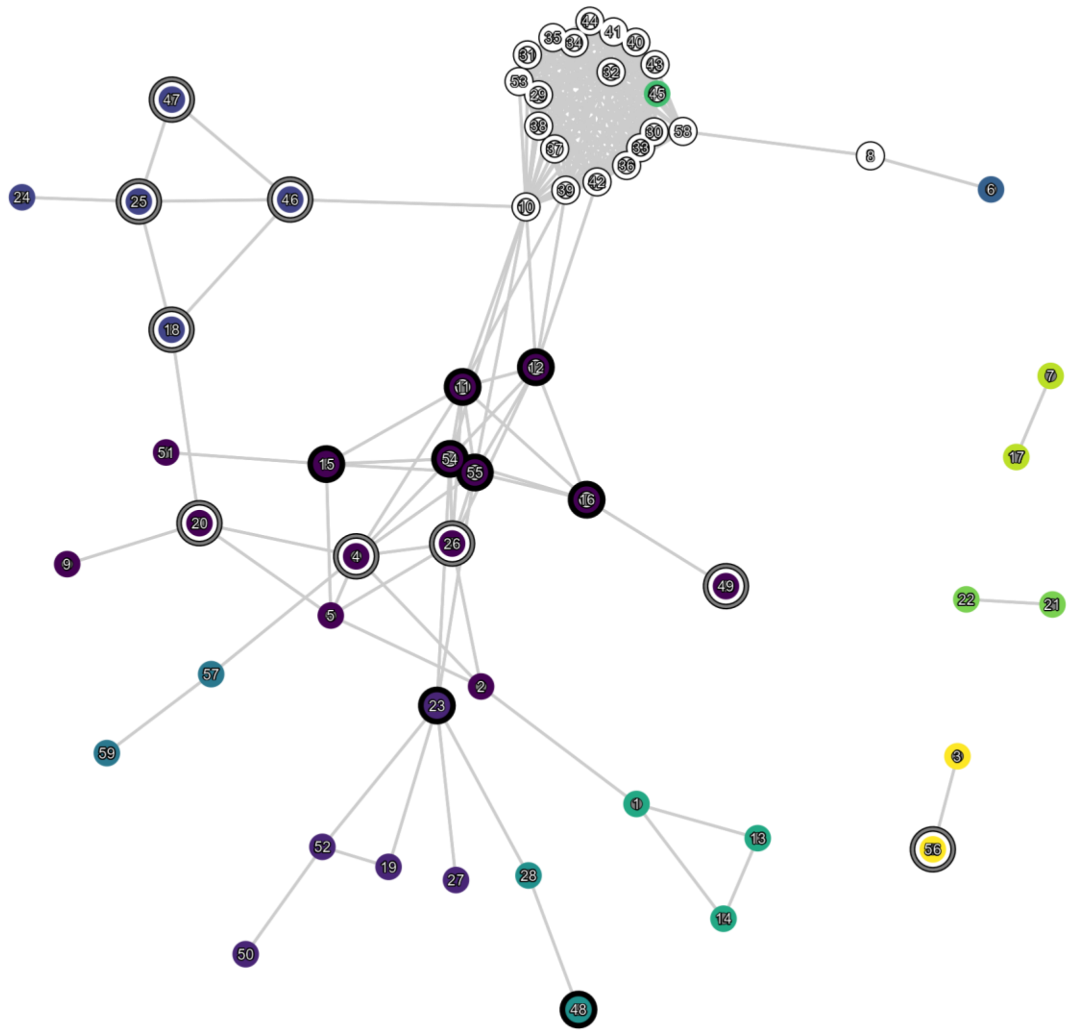

YMD4529-SLAD-2PE_vs_SHAD-2PE_up

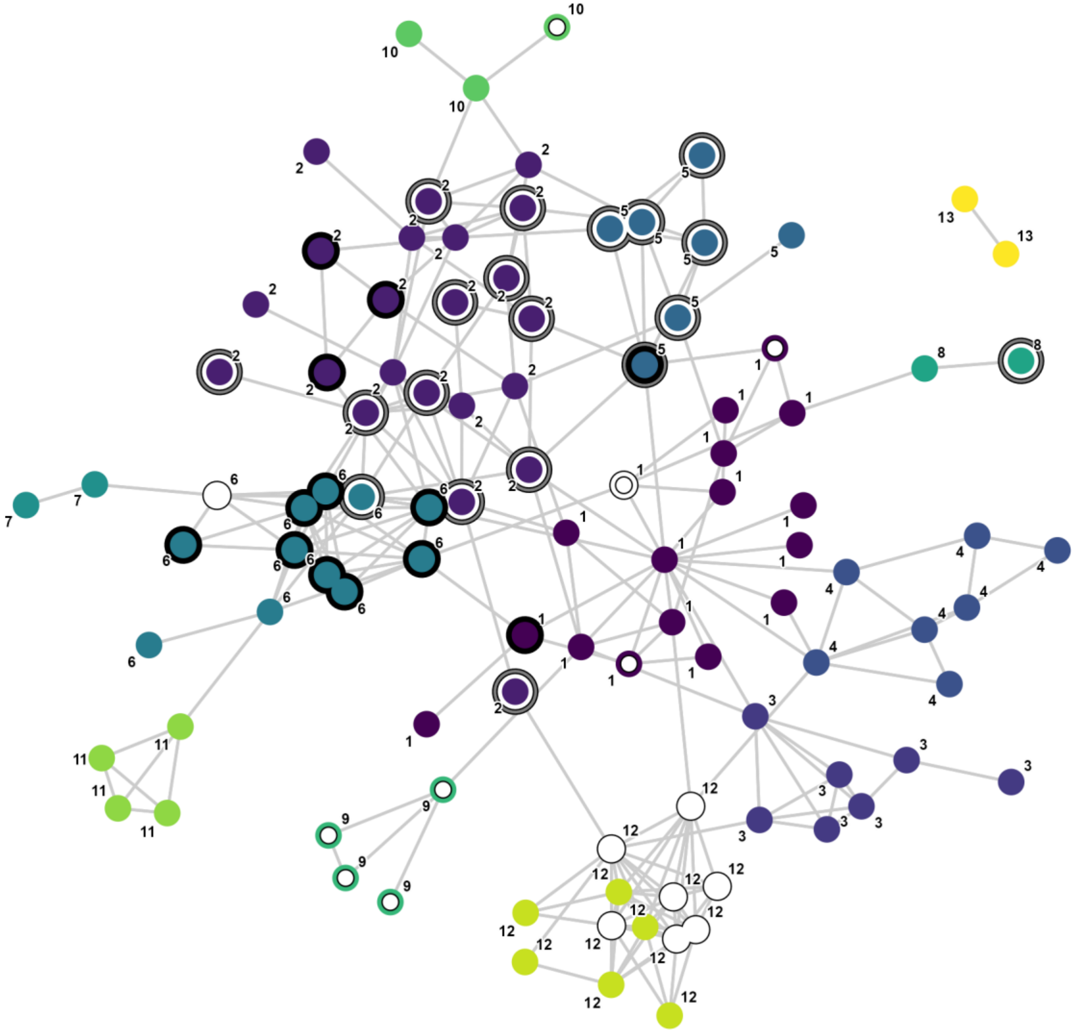

YMD4529-SLAD-2PE_vs_SHAD-2PE_up

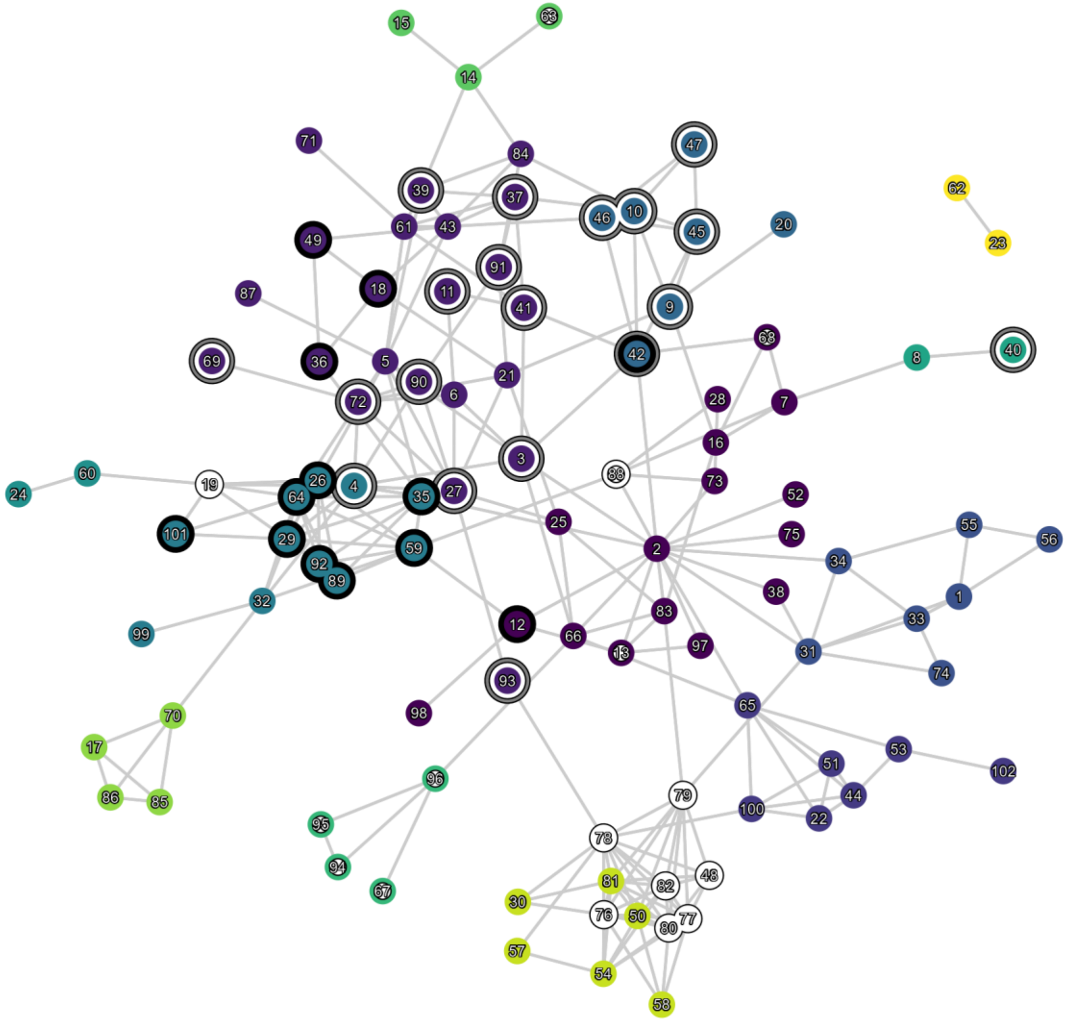

YMD4537-SLAD_vs_SHAD_down

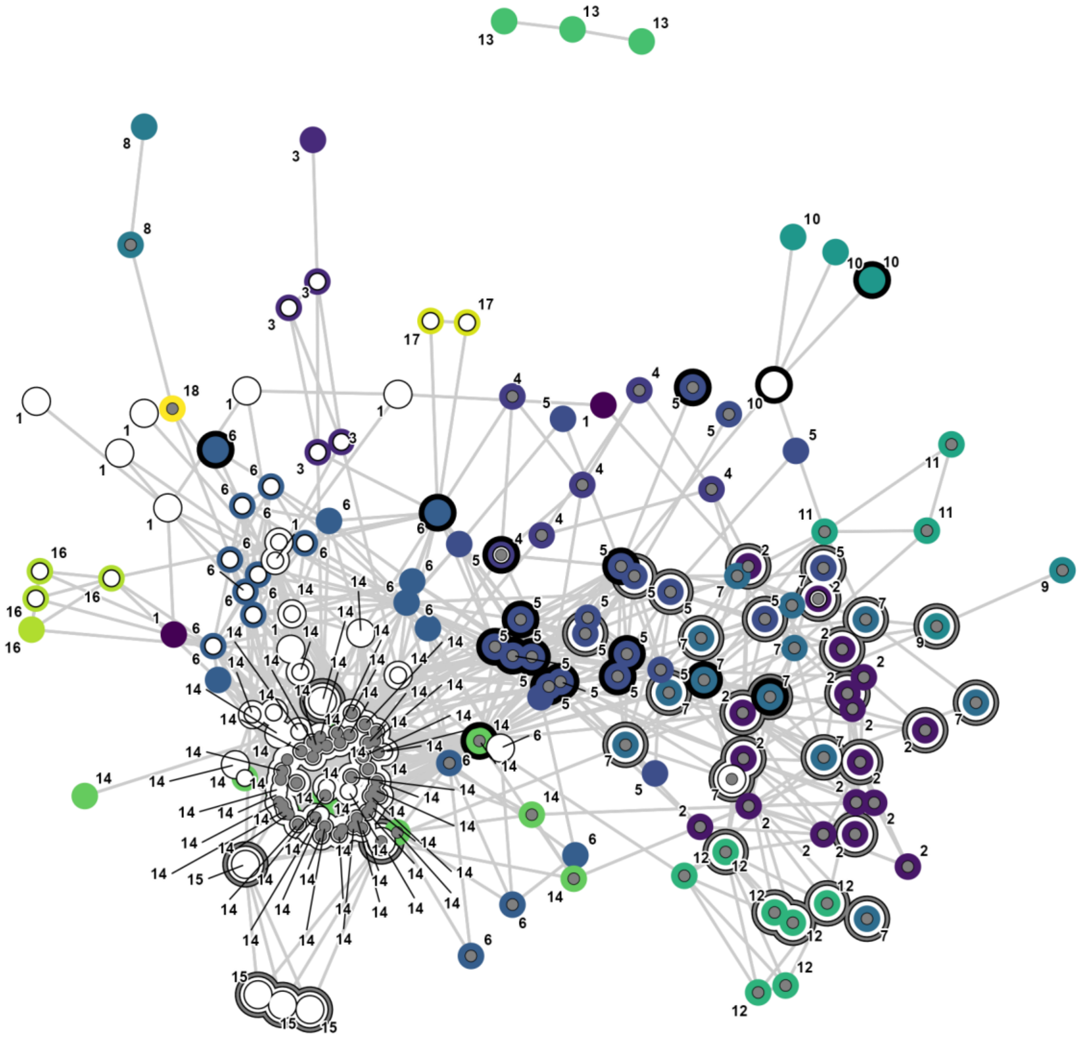

YMD4537-SLAD_vs_SHAD_down

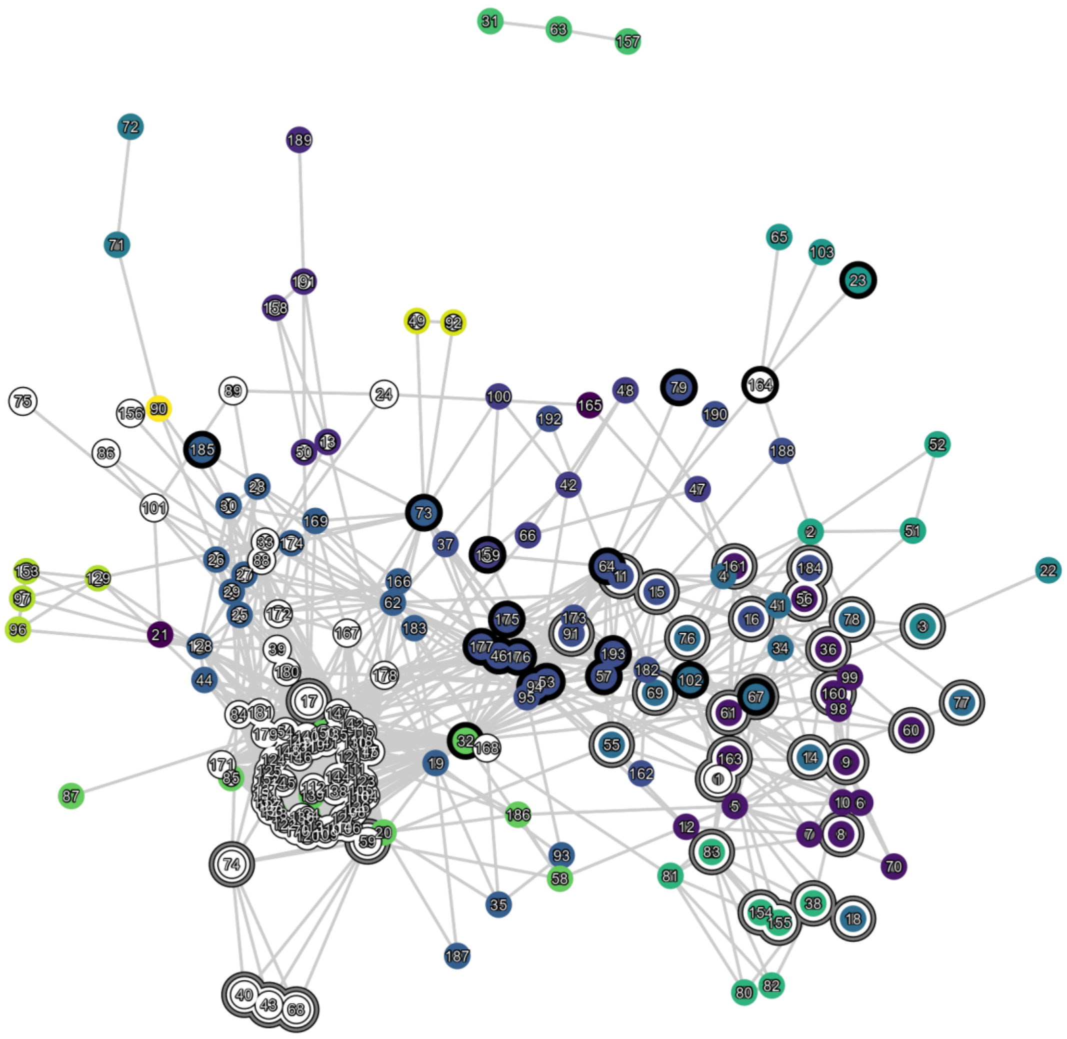

YMD4537-SLAD_vs_SHAD_up

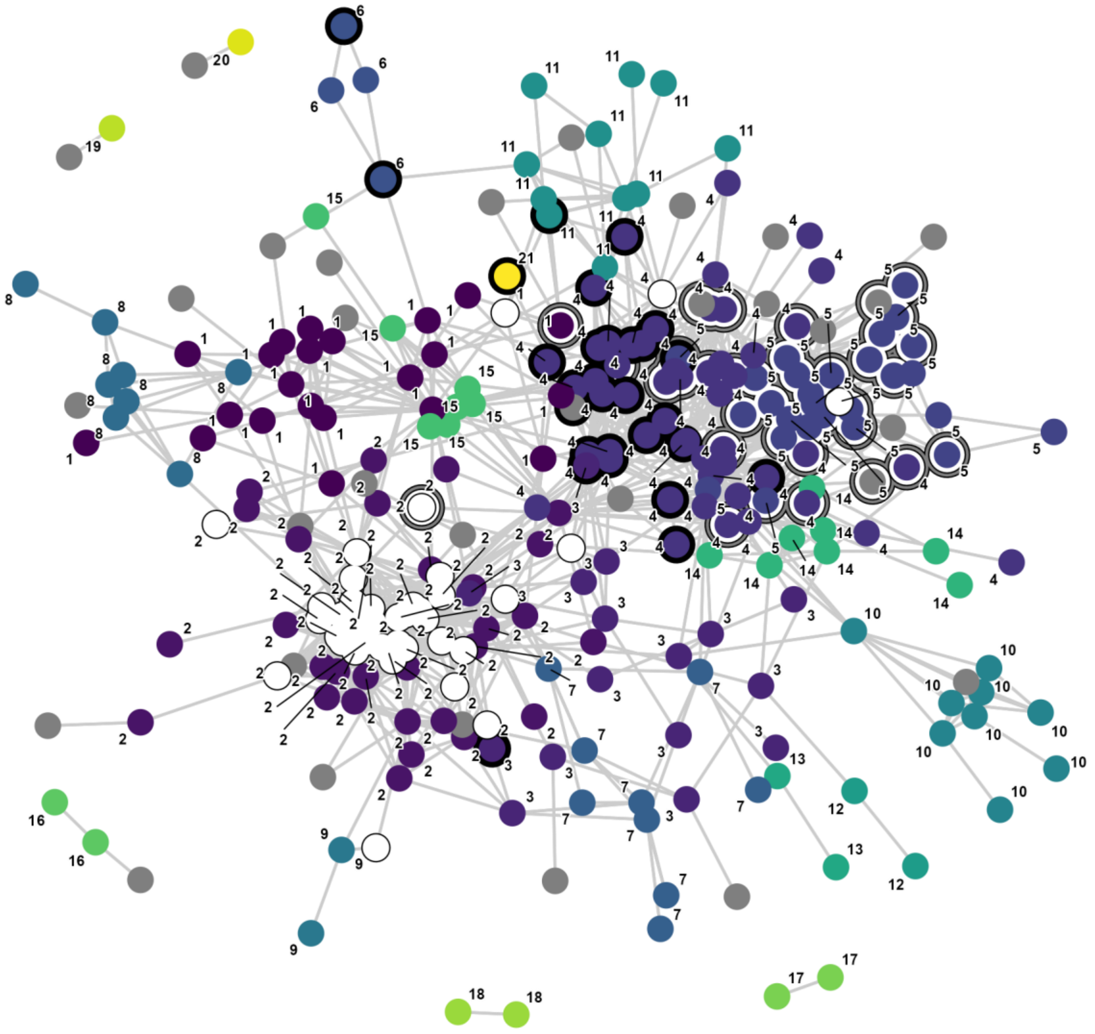

YMD4537-SLAD_vs_SHAD_up

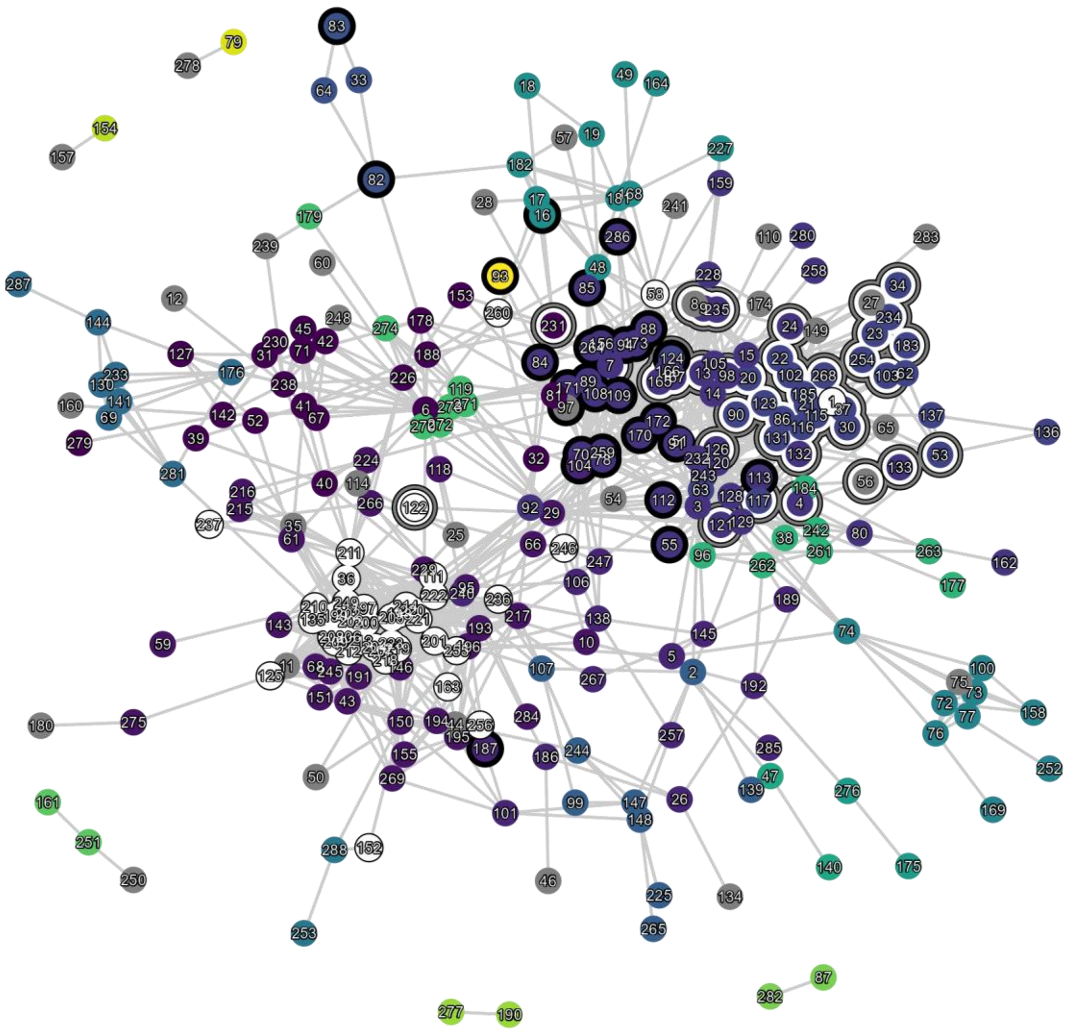

YMD4537-SLAD-2PE_vs_SHAD_down

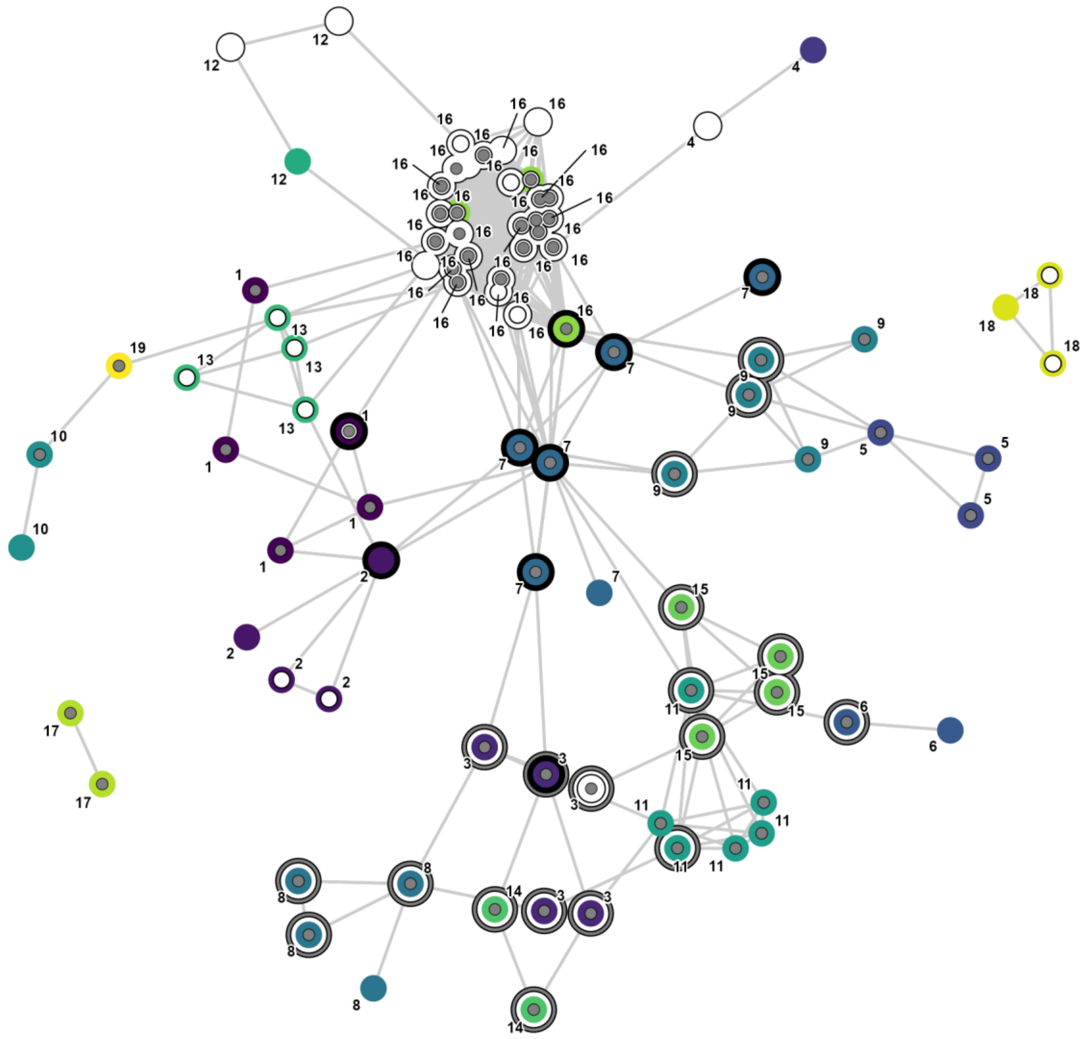

YMD4537-SLAD-2PE_vs_SHAD_down

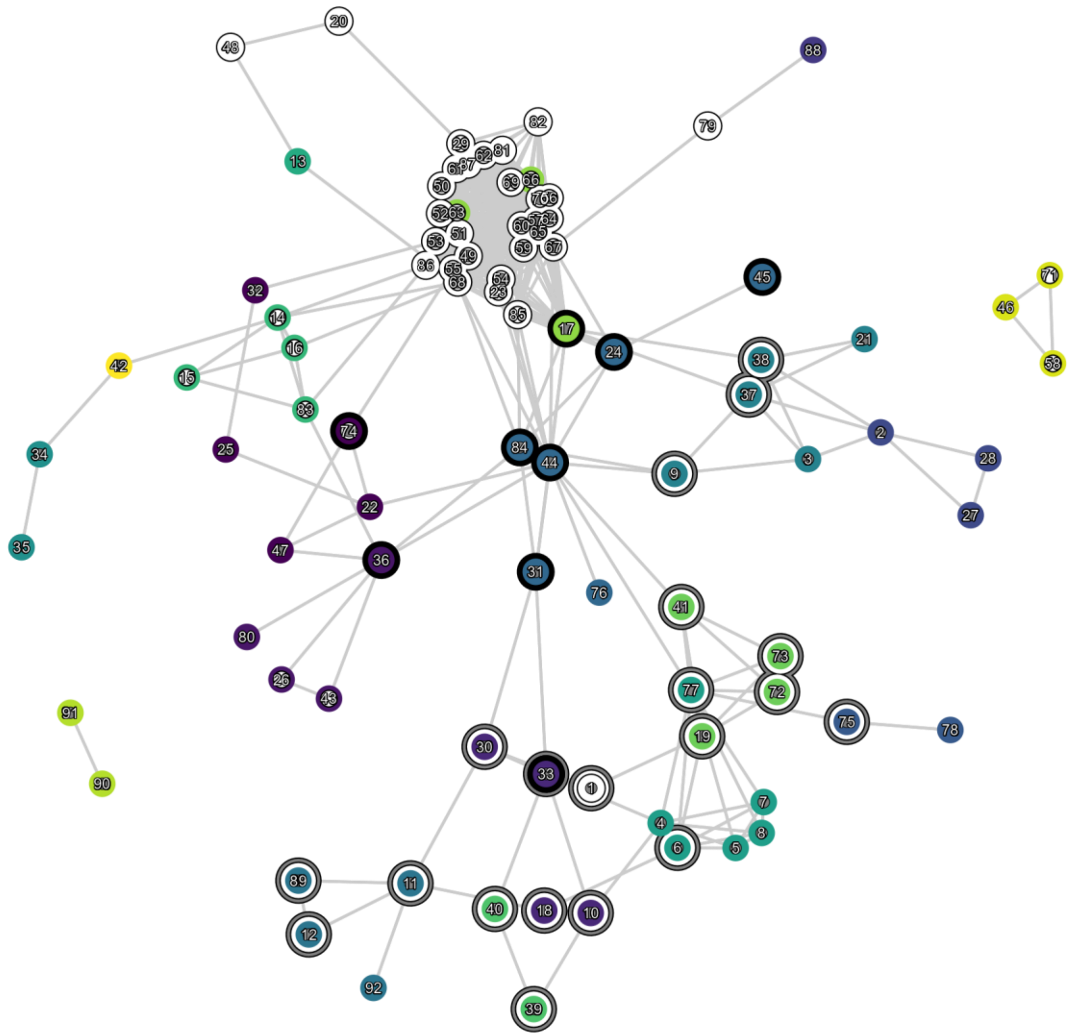

YMD4537-SLAD-2PE_vs_SHAD_up

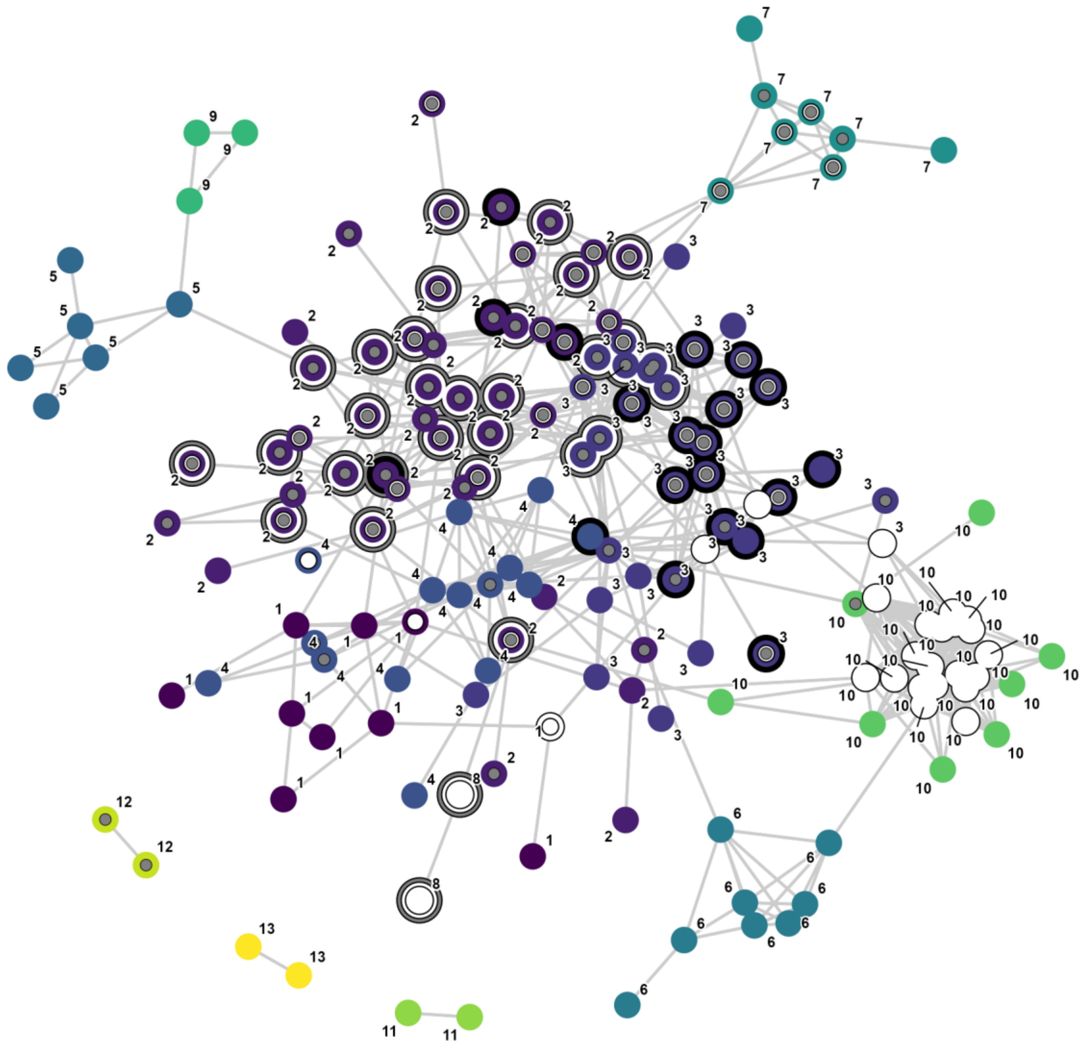

YMD4537-SLAD-2PE_vs_SHAD_up

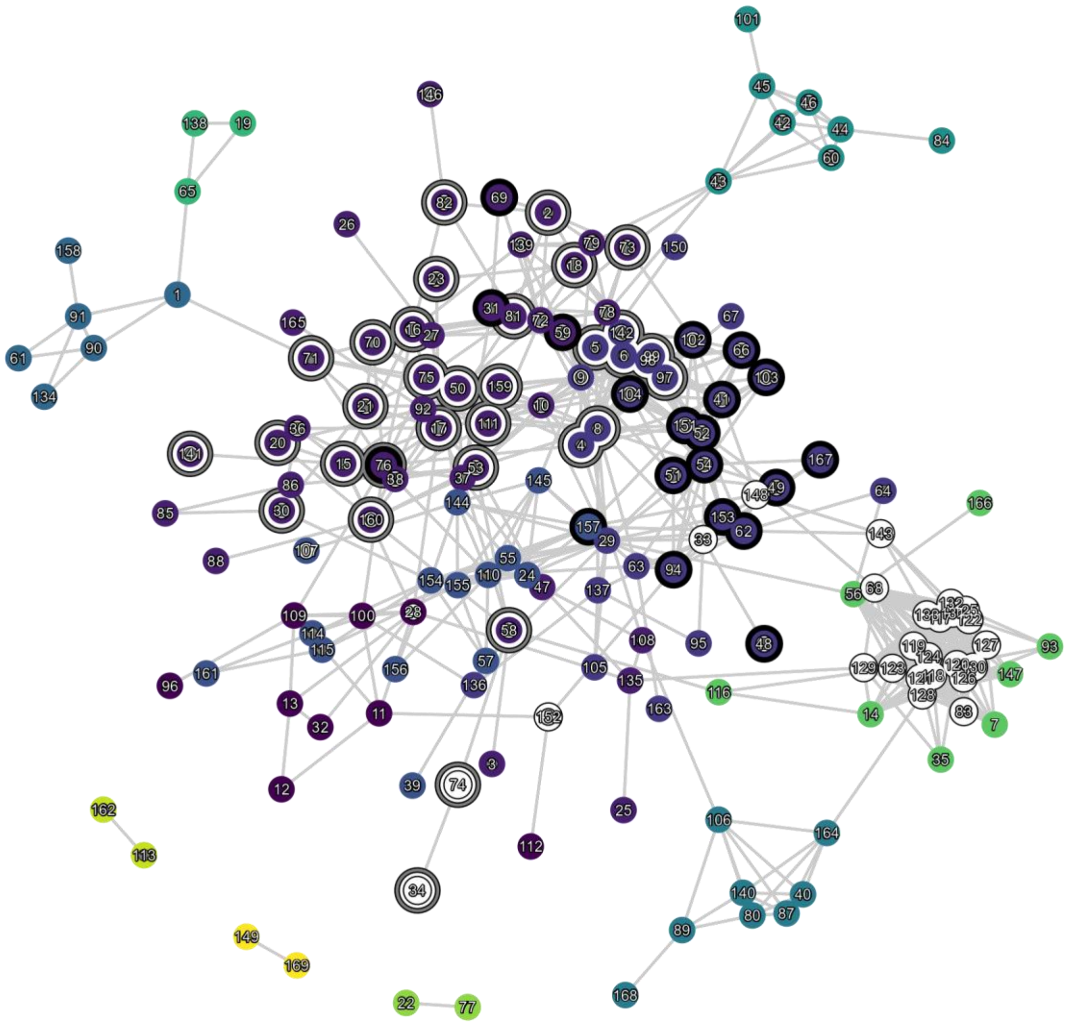

YMD4537-SLAD-2PE_vs_SHAD-2PE_down

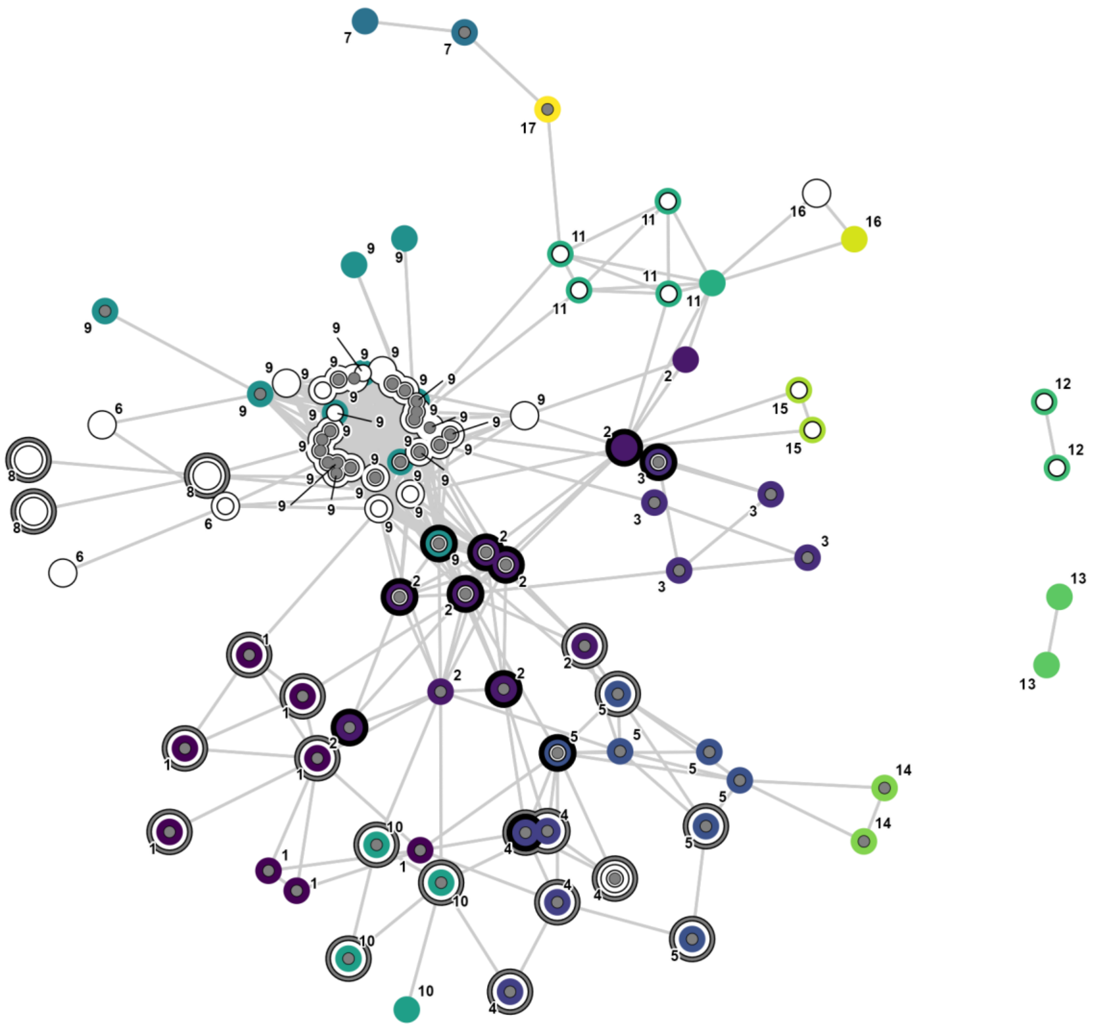

YMD4537-SLAD-2PE_vs_SHAD-2PE_down

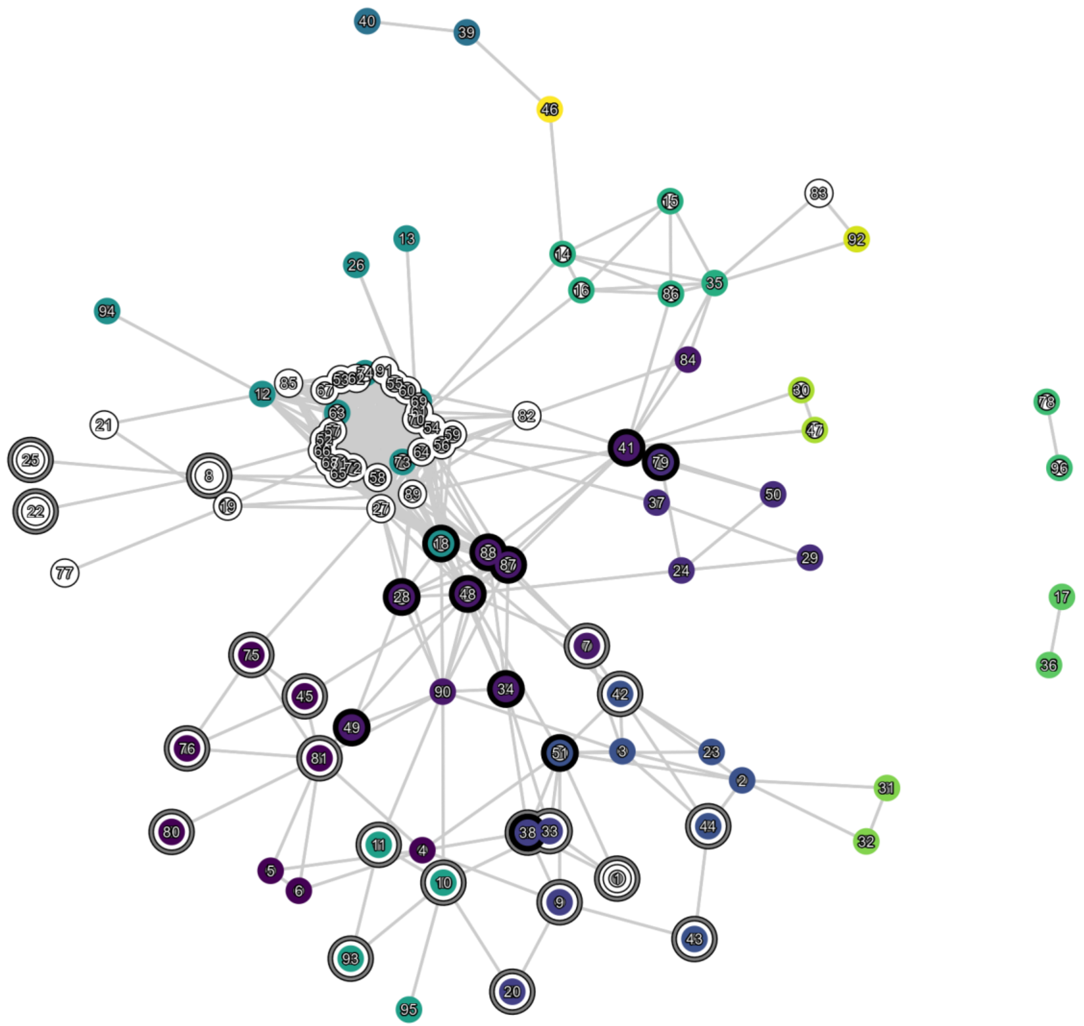

YMD4537-SLAD-2PE_vs_SHAD-2PE_up

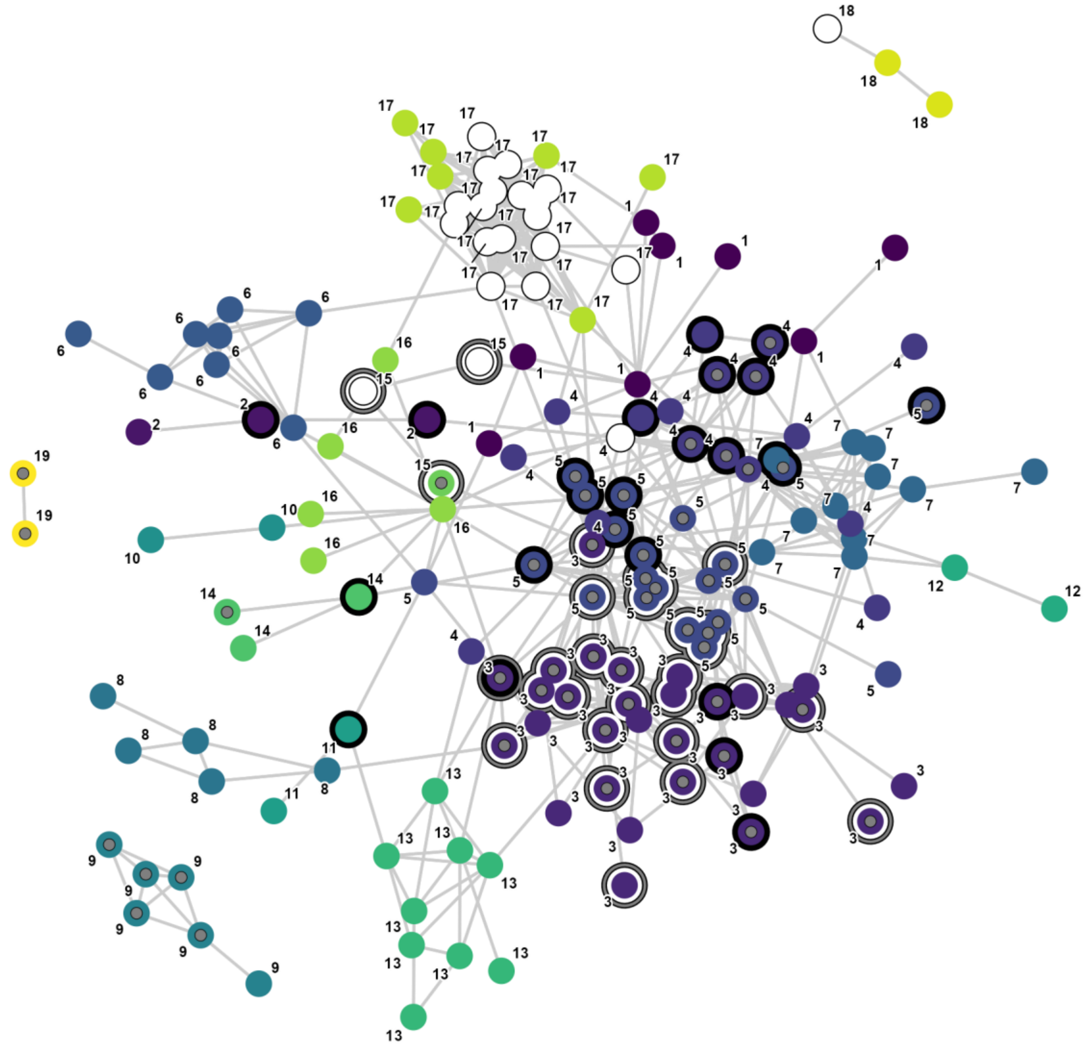

YMD4537-SLAD-2PE_vs_SHAD-2PE_up

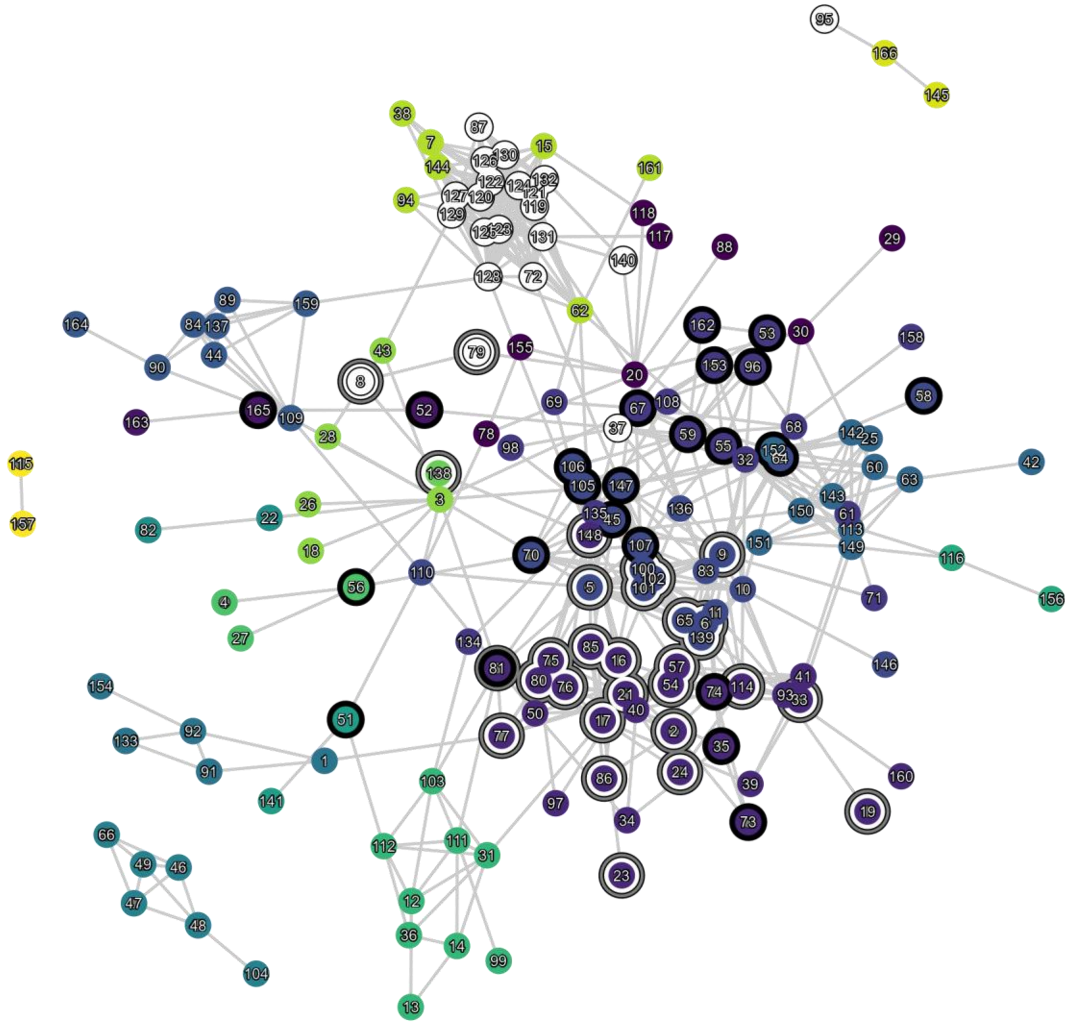

YMD4544-SLAD-2PE_vs_SHAD_down

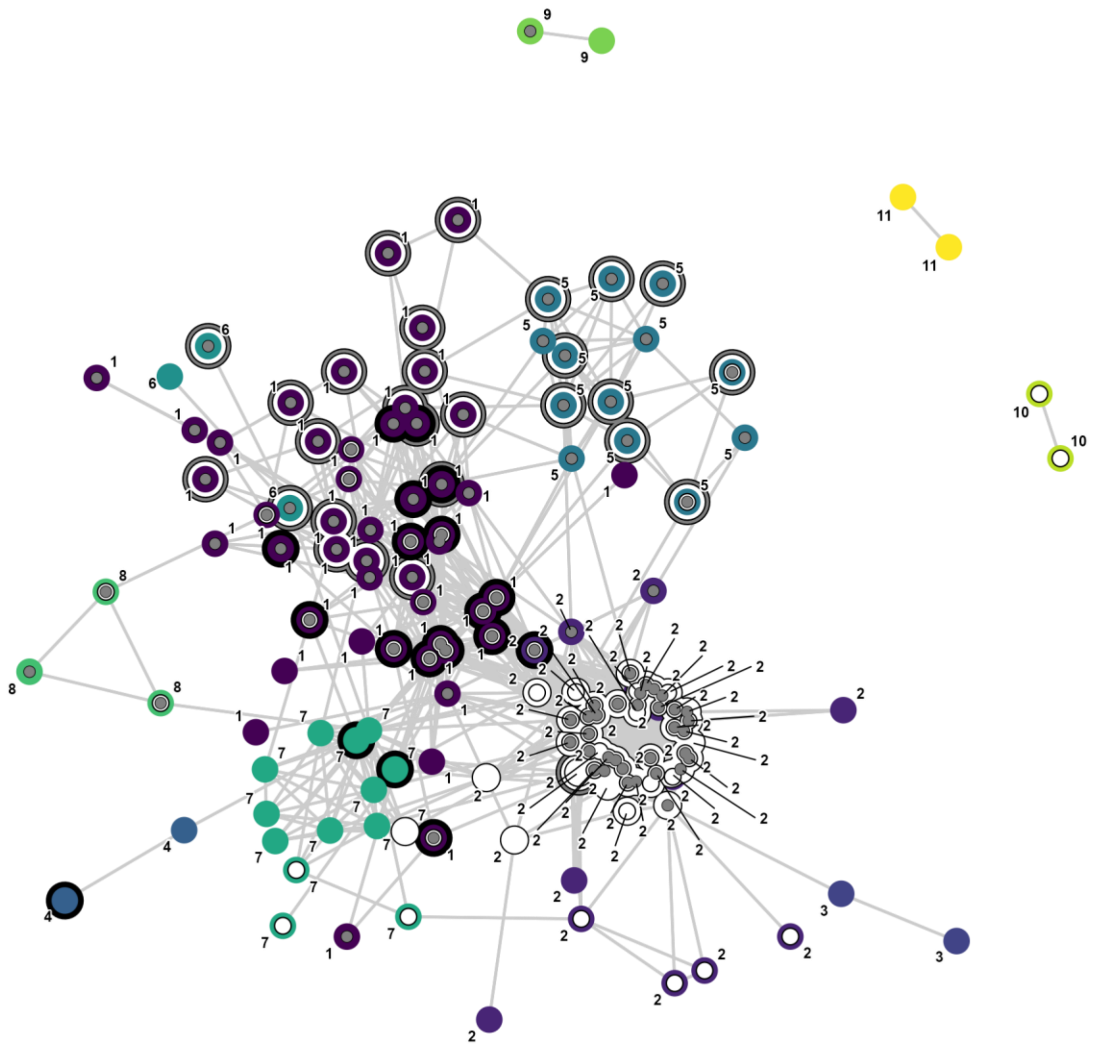

YMD4544-SLAD-2PE_vs_SHAD_down

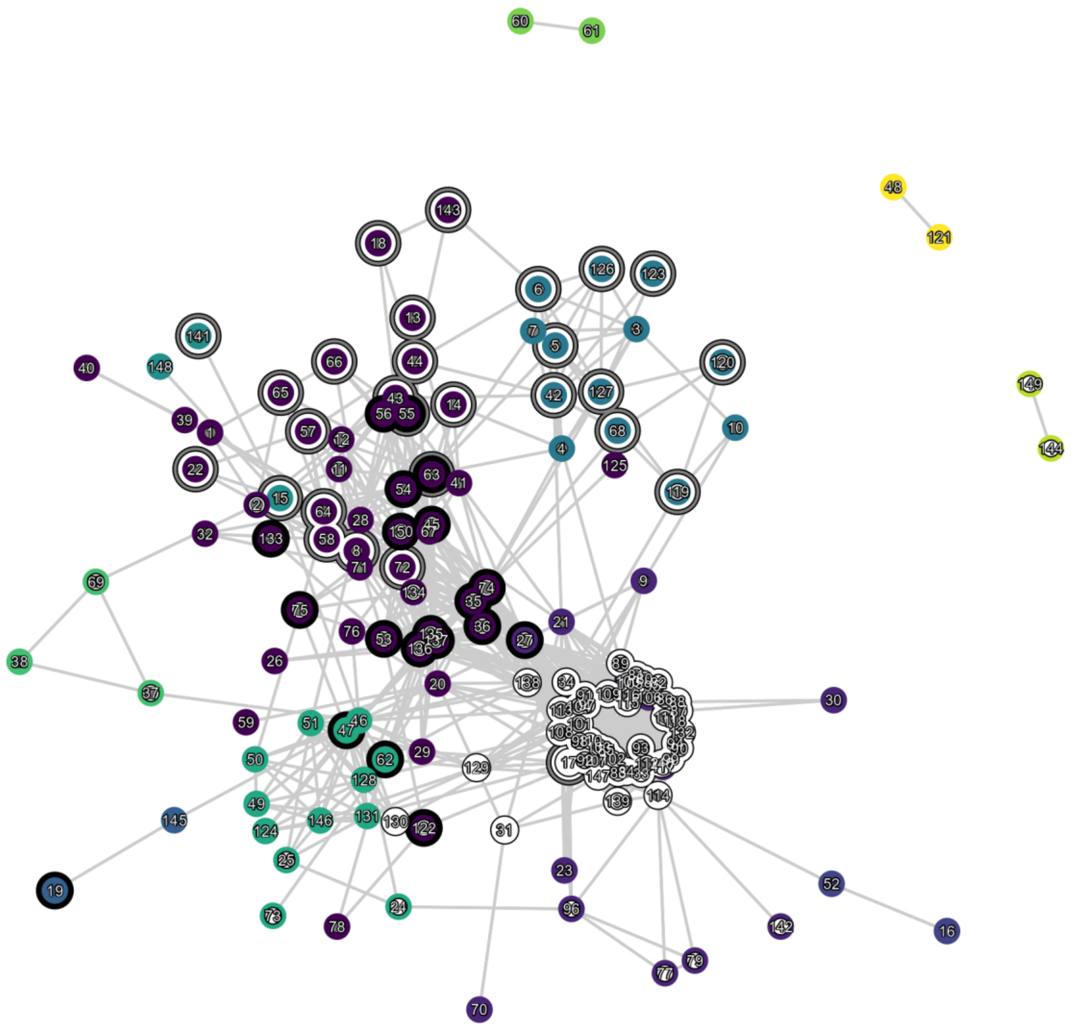

YMD4544-SLAD-2PE_vs_SHAD_up

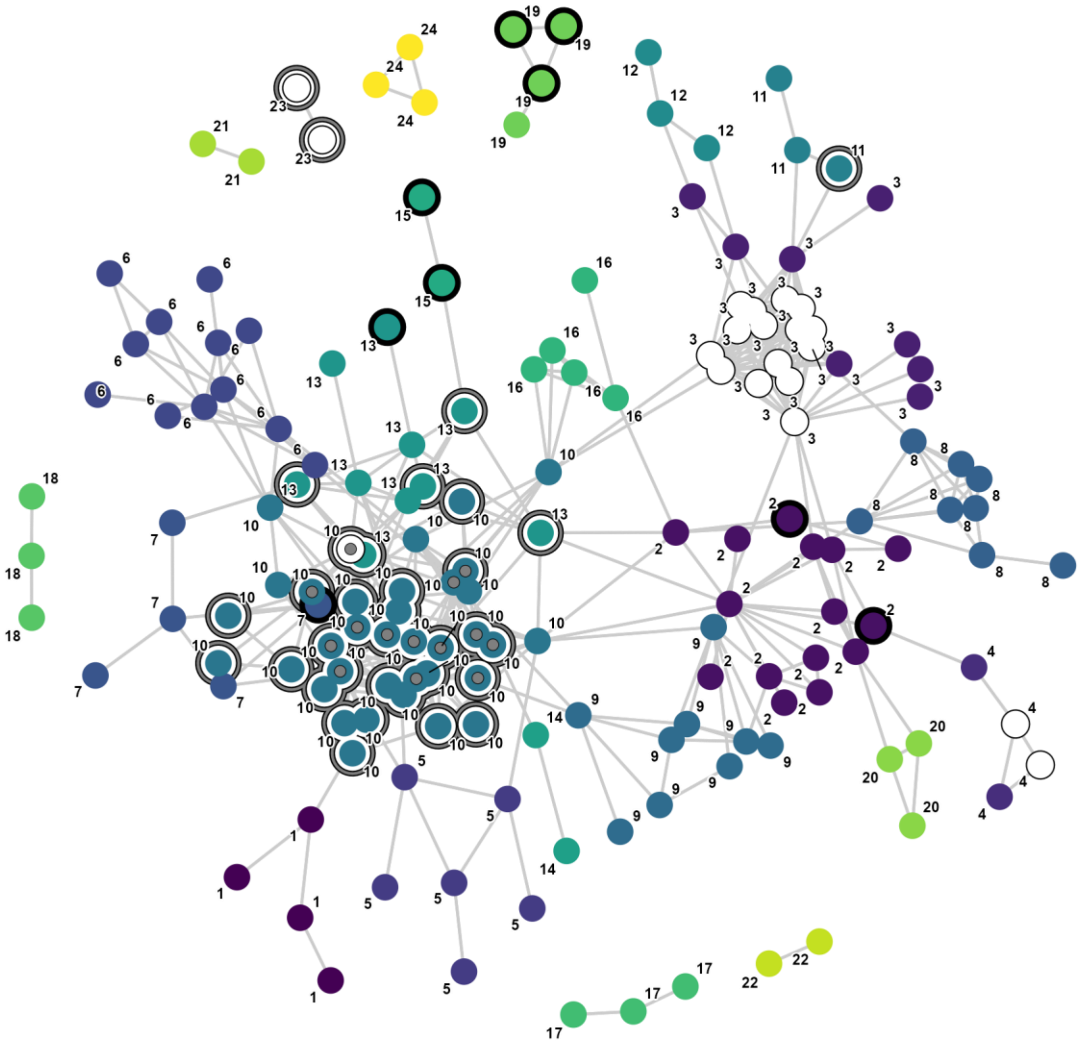

YMD4544-SLAD-2PE_vs_SHAD_up

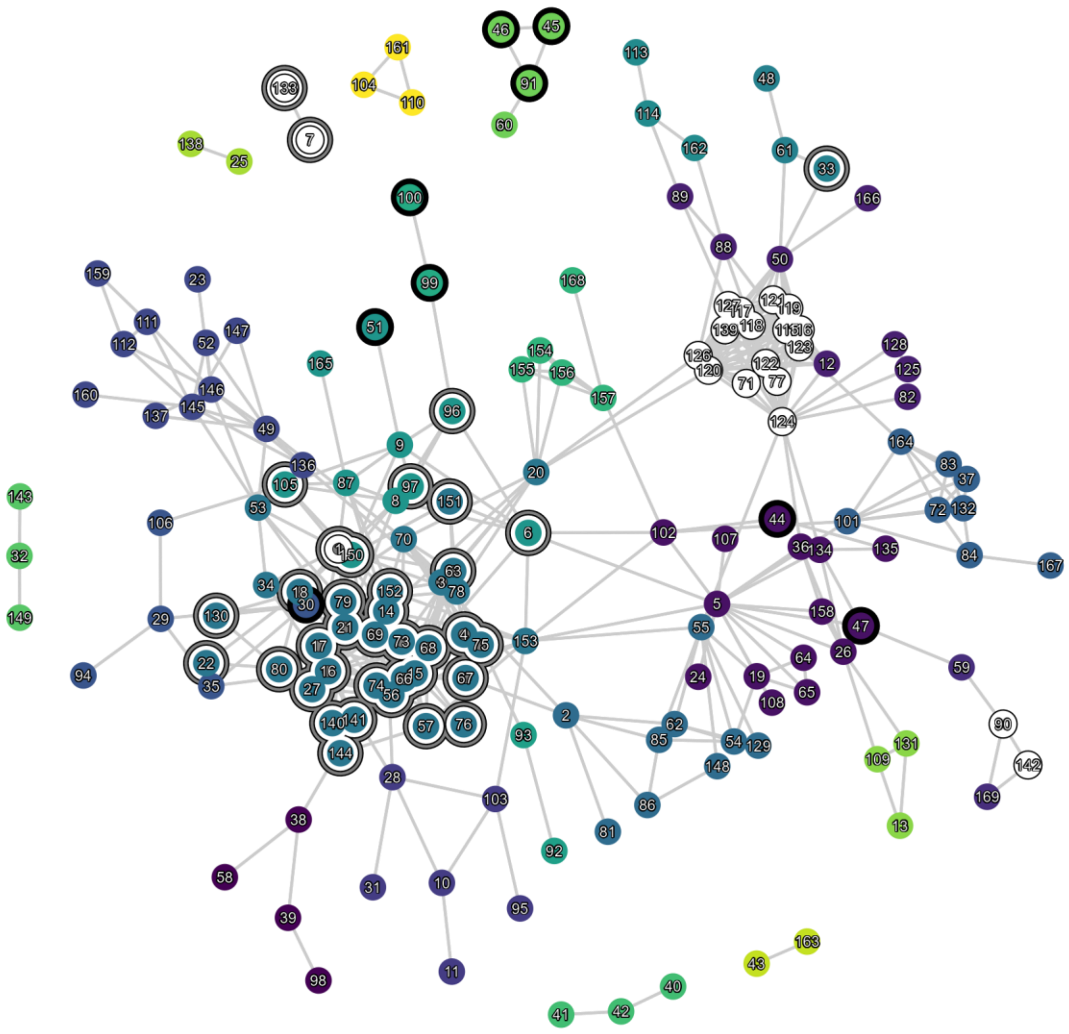

YMD4544-SLAD-2PE_vs_SHAD-2PE_down

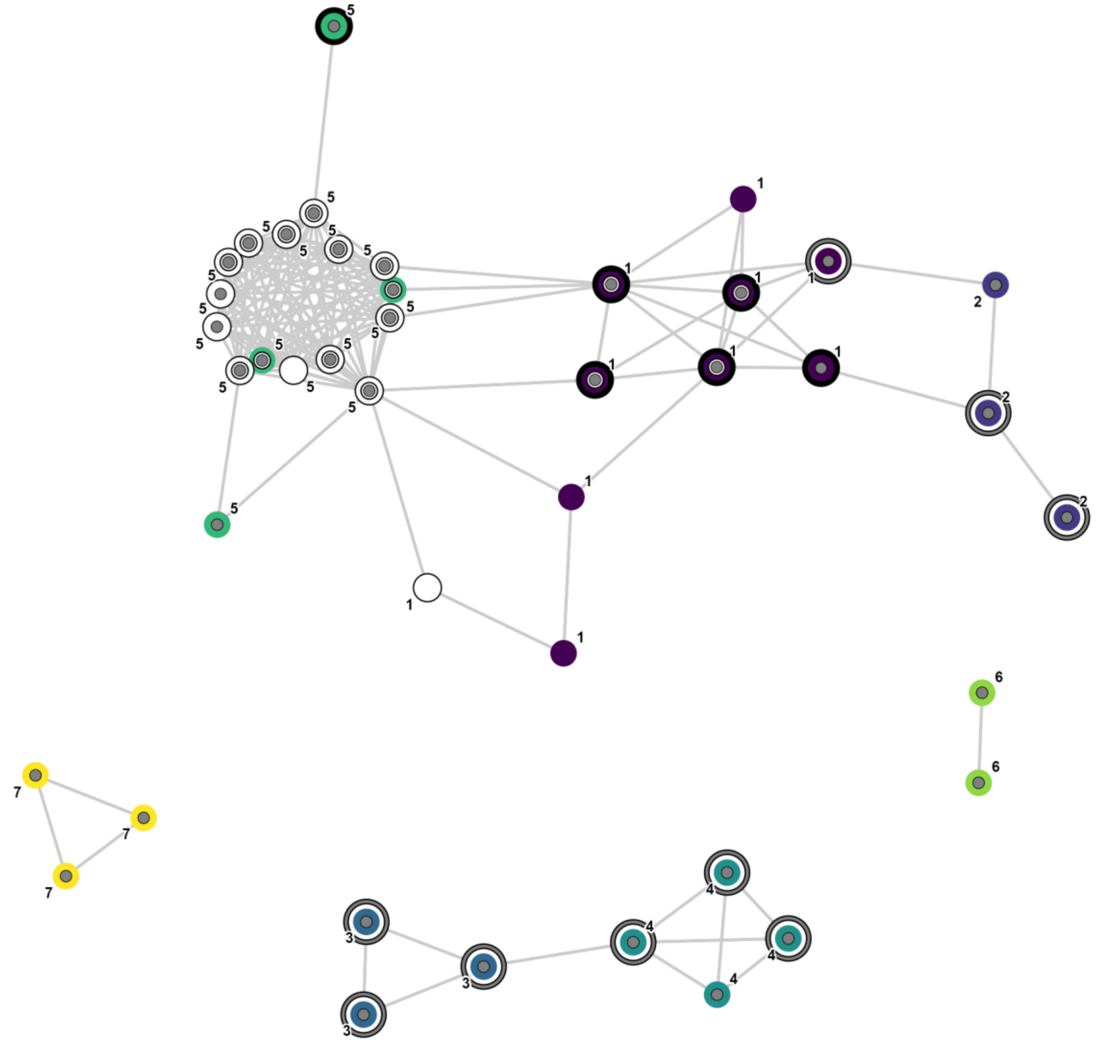

YMD4544-SLAD-2PE_vs_SHAD-2PE_down

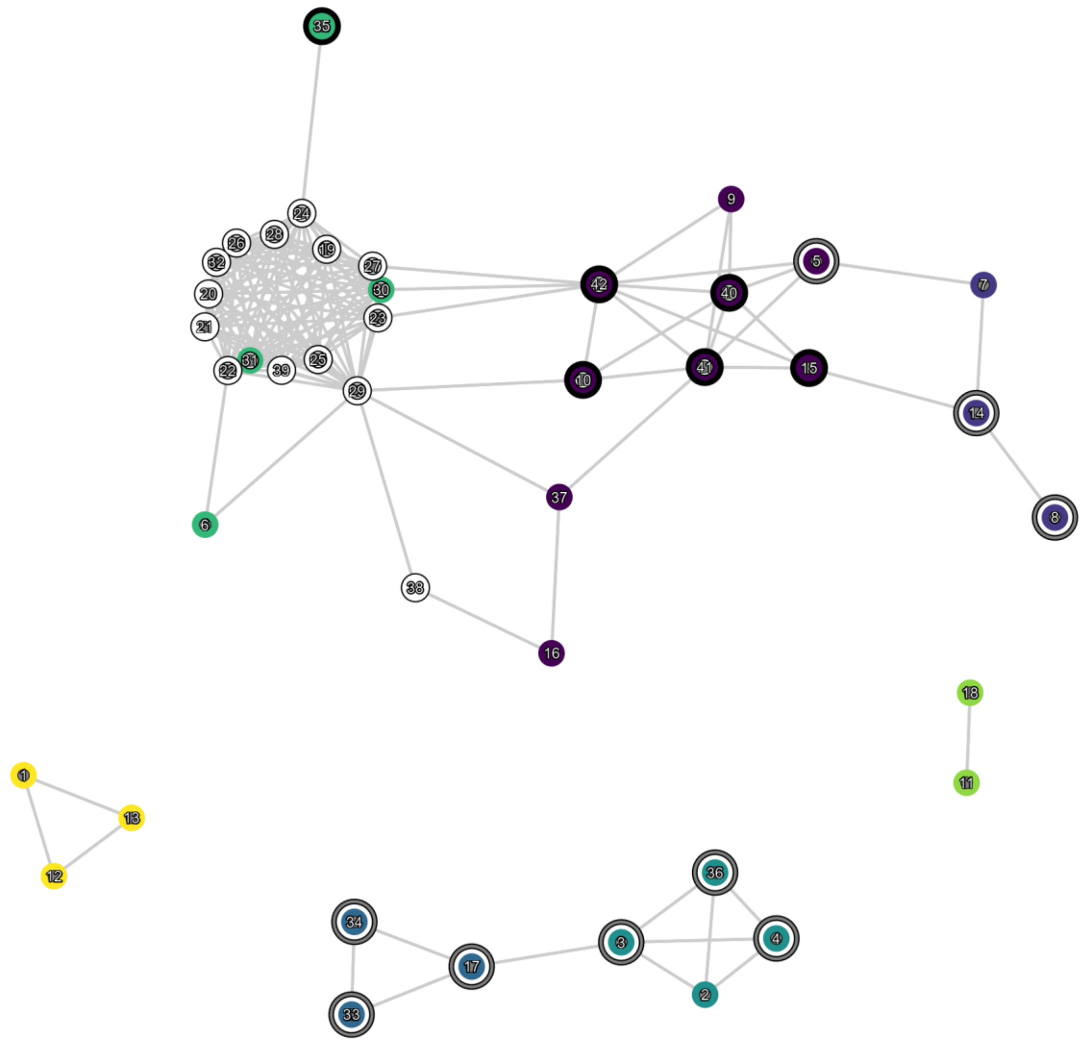

YMD4544-SLAD-2PE_vs_SHAD-2PE_up

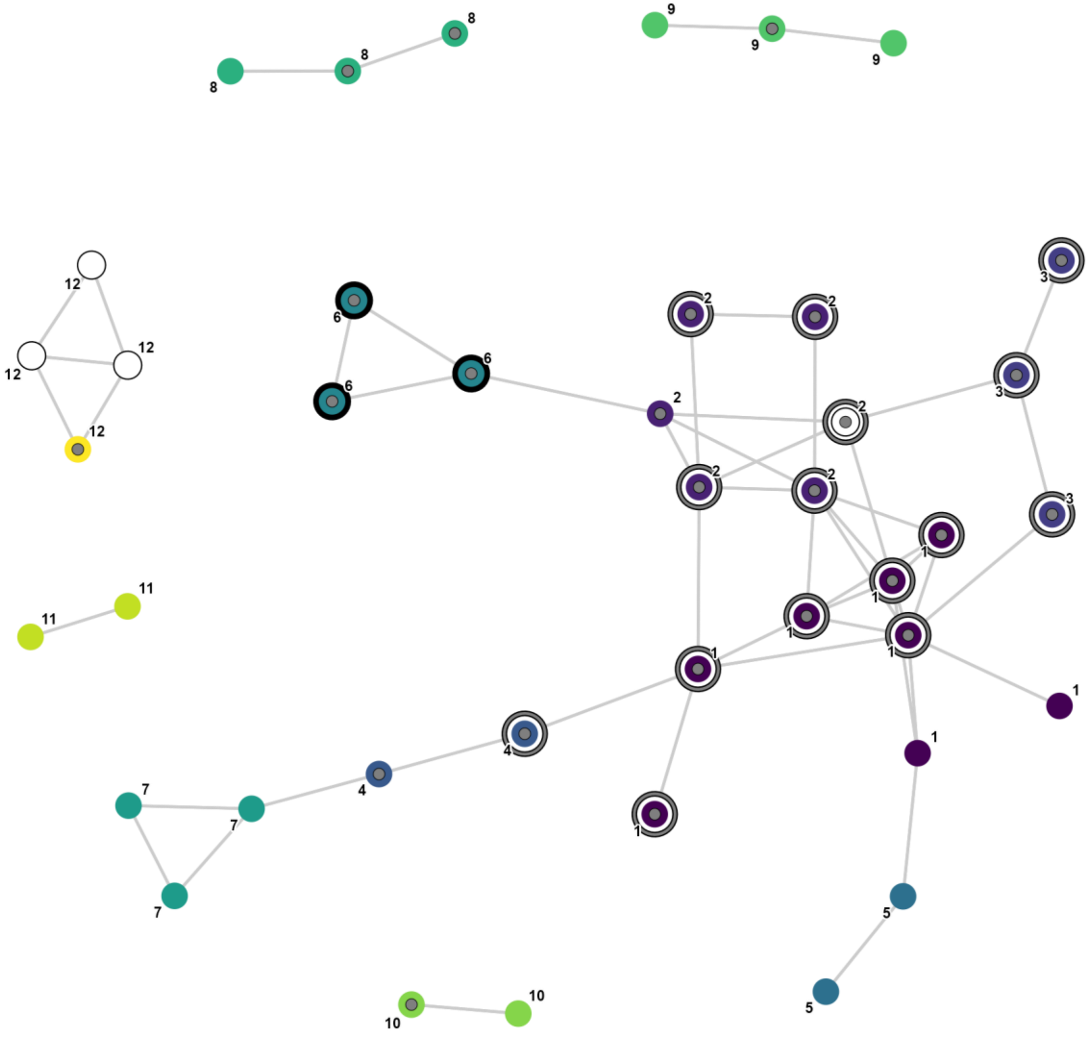

YMD4544-SLAD-2PE_vs_SHAD-2PE_up

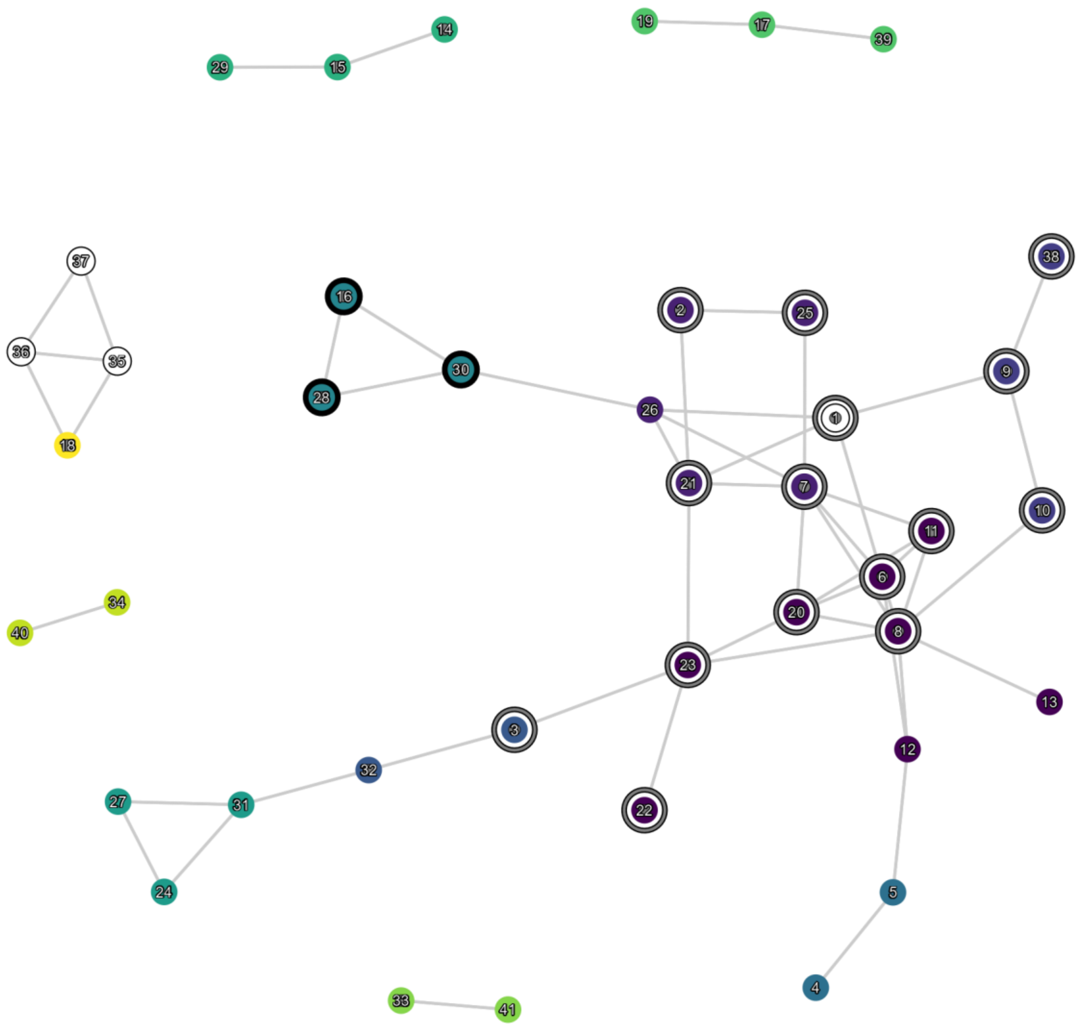

Supplement: foaf036_Supplemental_Files [file foaf036_supplemental_files.zip › Supplement_Figure_02_Proteomics_Networks.pdf]
