# Supplementary material for: Quorum sensing in Saccharomyces cerevisiae brewing strains: effects of 2-phenylethanol on proteomic, lipidomic, and metabolomic profile
Source: FEMS Yeast Res. 2025 Jul 7;25:foaf036. doi: 10.1093/femsyr/foaf036 (PMC12254953; doi:10.1093/femsyr/foaf036)
Supplement: foaf036_Supplemental_Files [file foaf036_supplemental_files.zip › Supplement_Figure_03_Metabolomics_Networks.pdf]

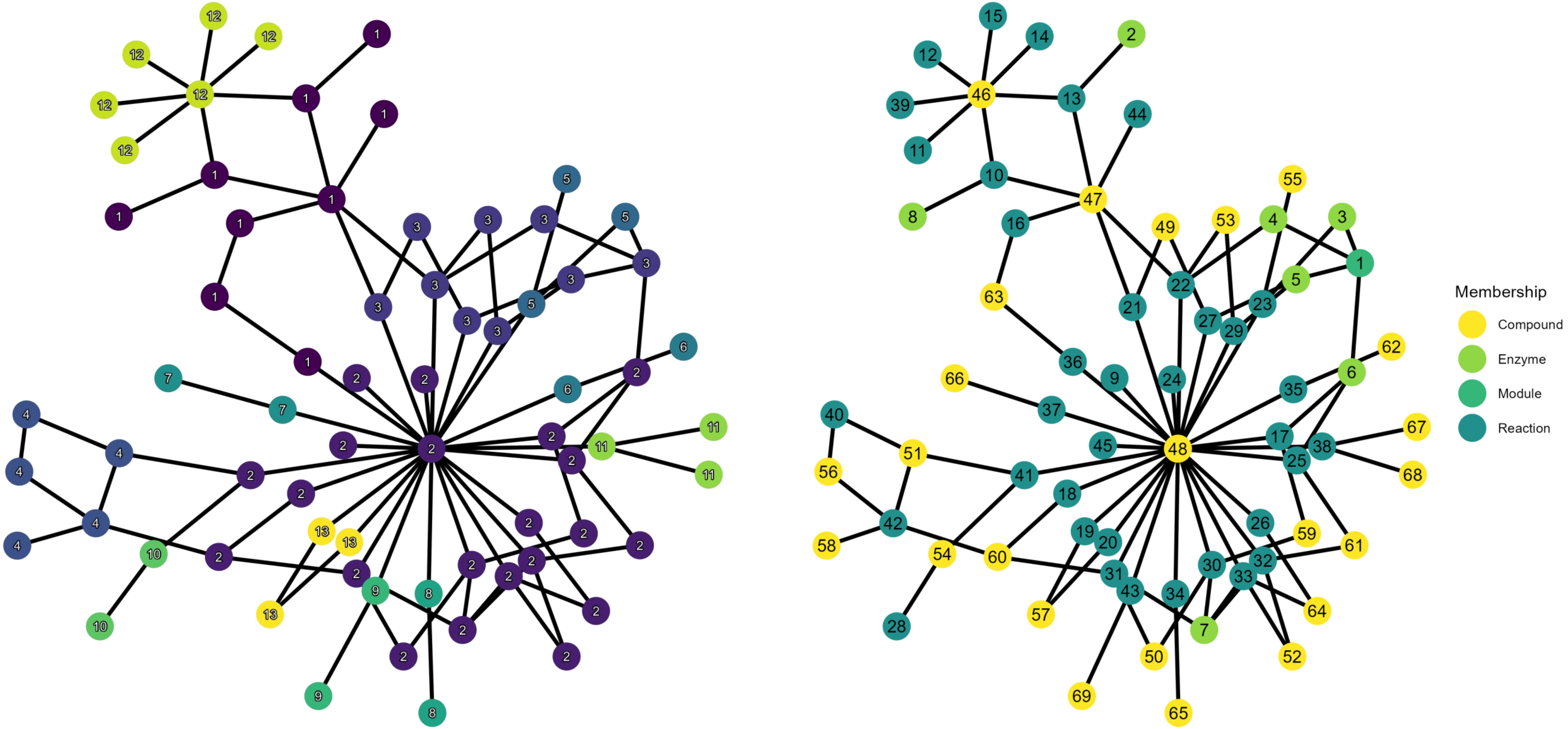

YMD4529_SLAD_vs_SHAD

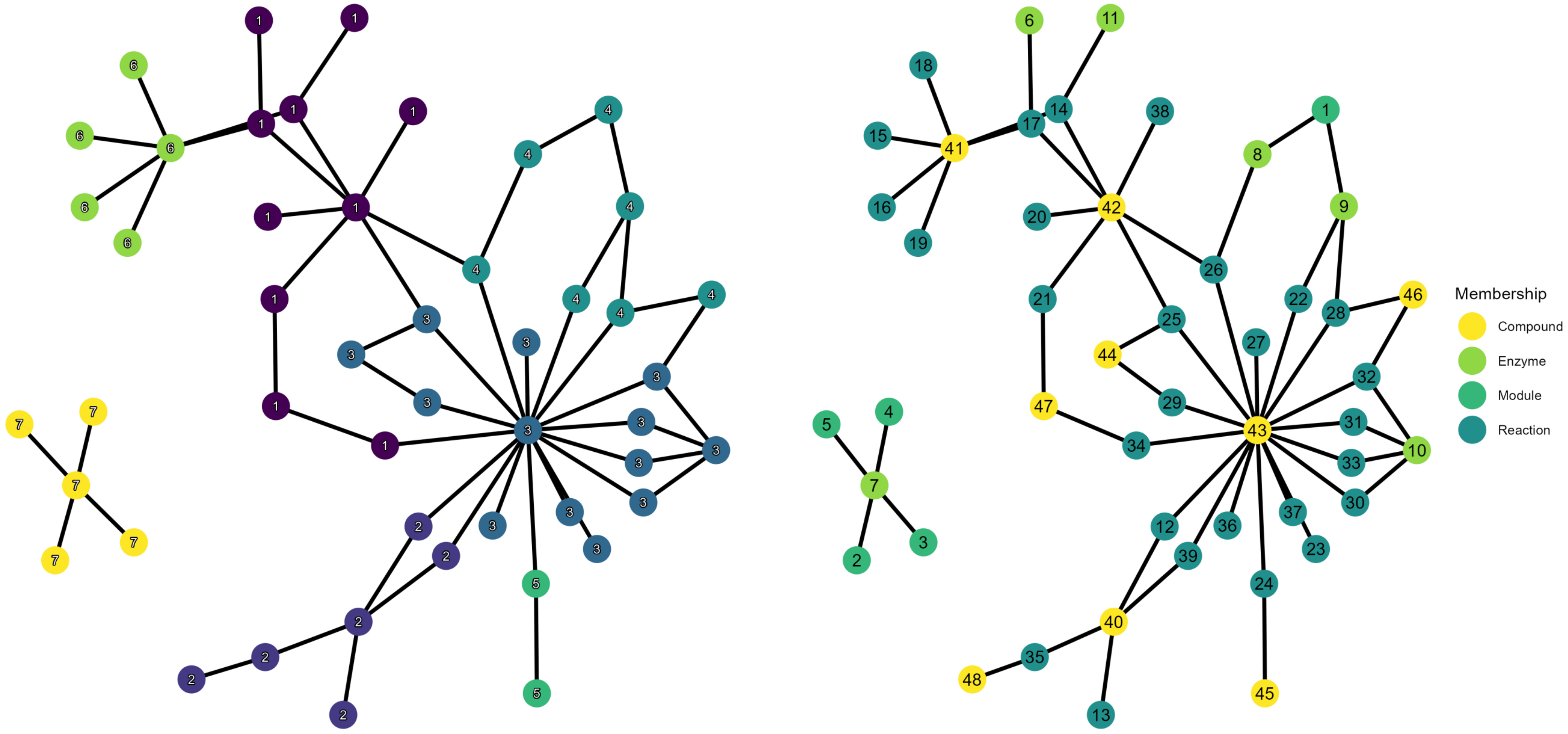

YMD4529_SLAD-2PE_vs_SHAD

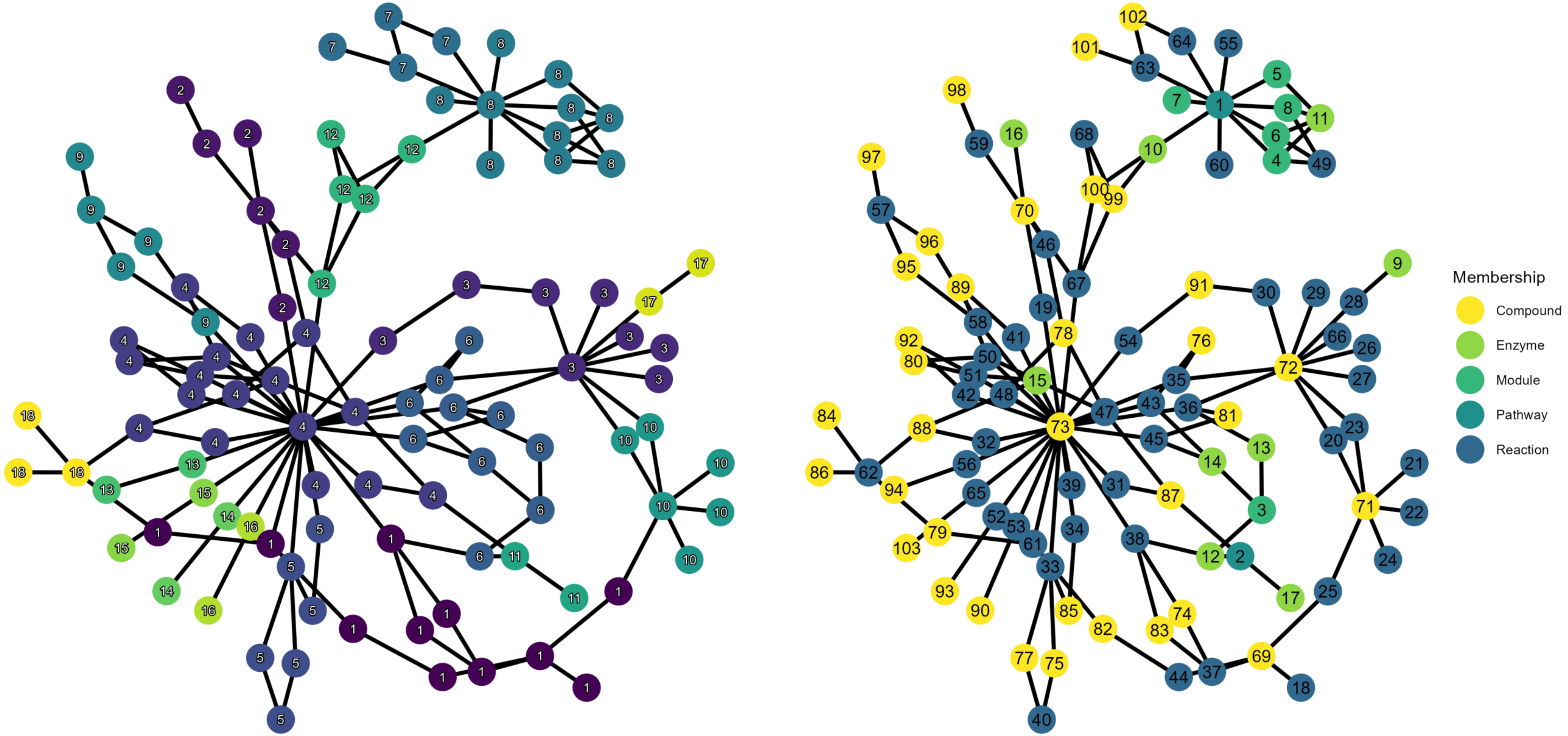

YMD4529_SLAD-2PE_vs_SHAD-2PE

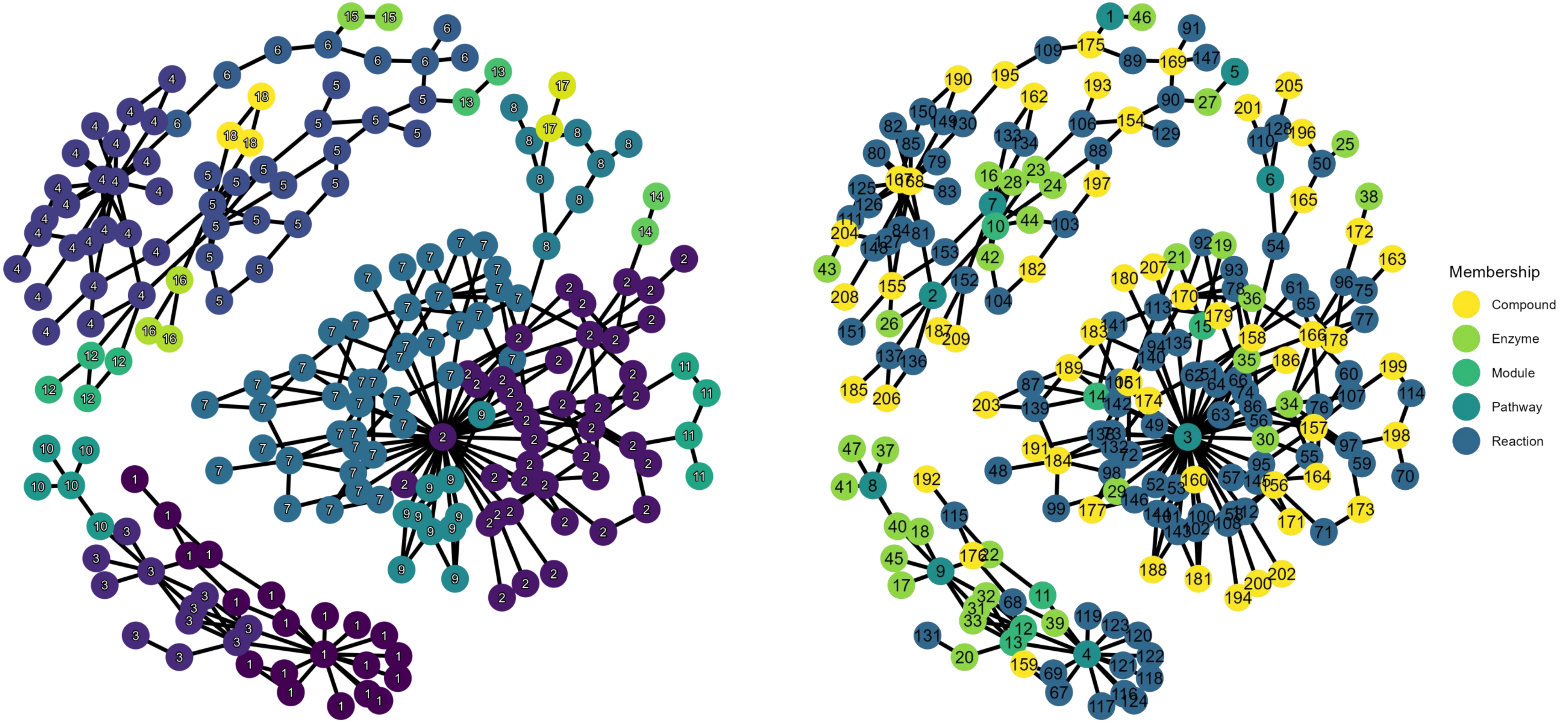

YMD4537_SLAD_vs_SHAD

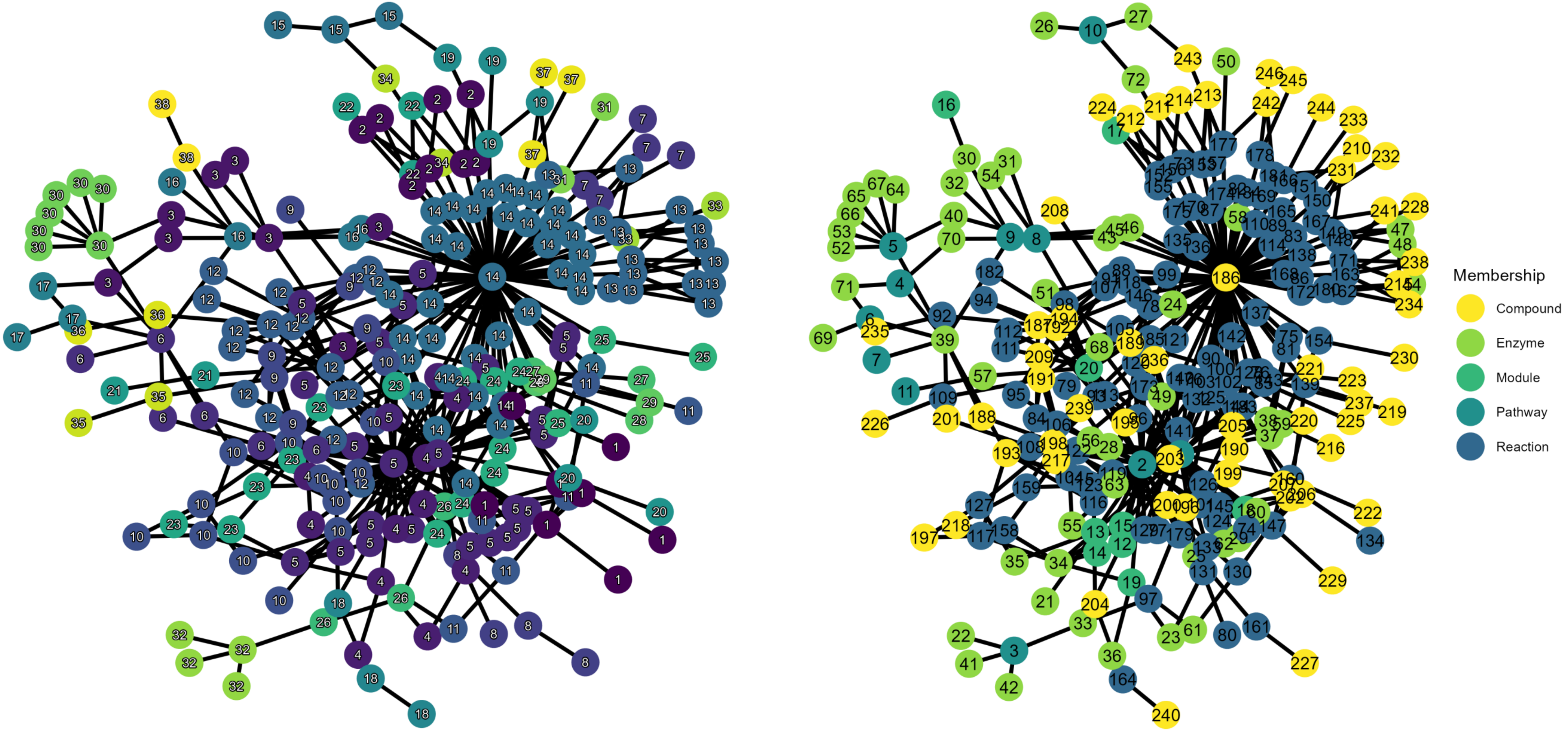

YMD4537_SHAD_vs_SHAD-2PE

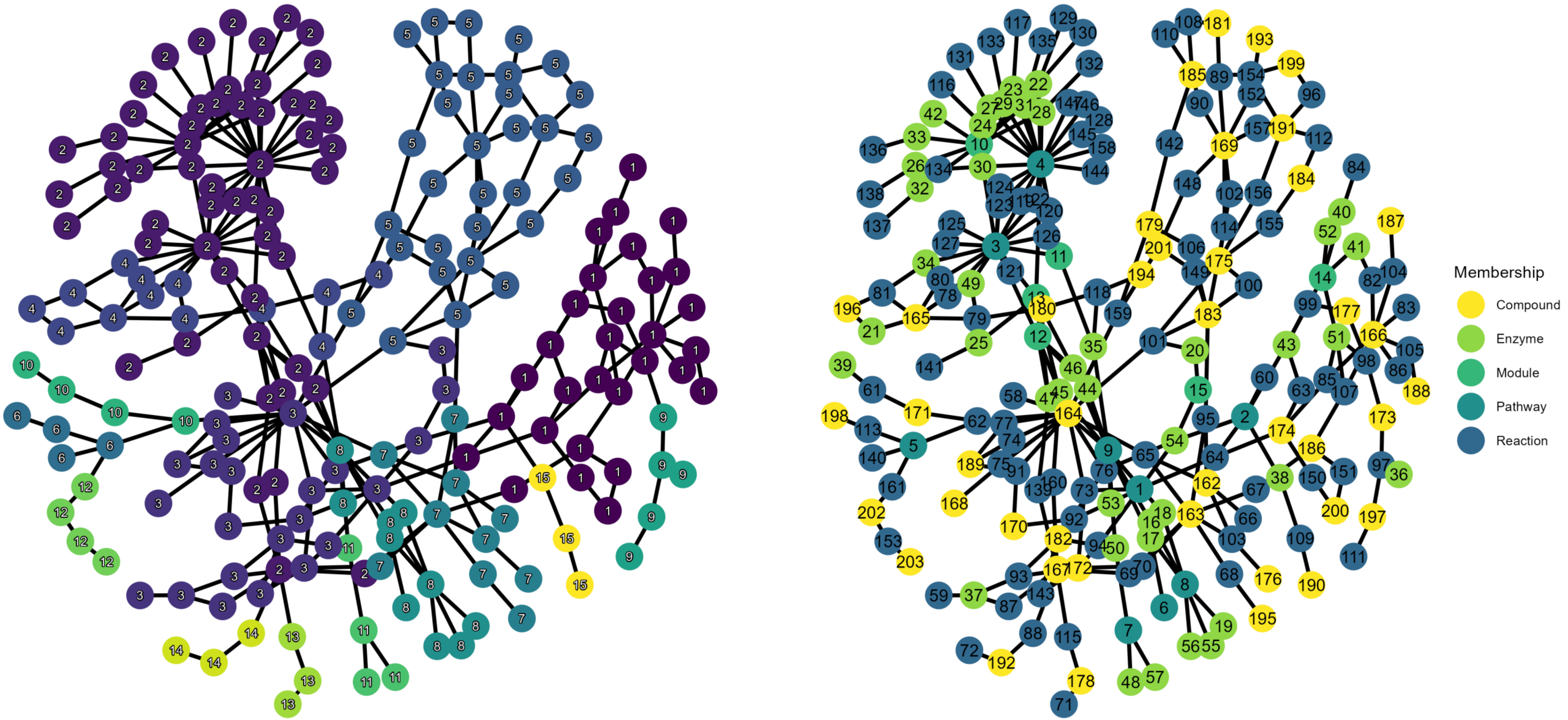

YMD4537_SLAD-2PE_vs_SHAD

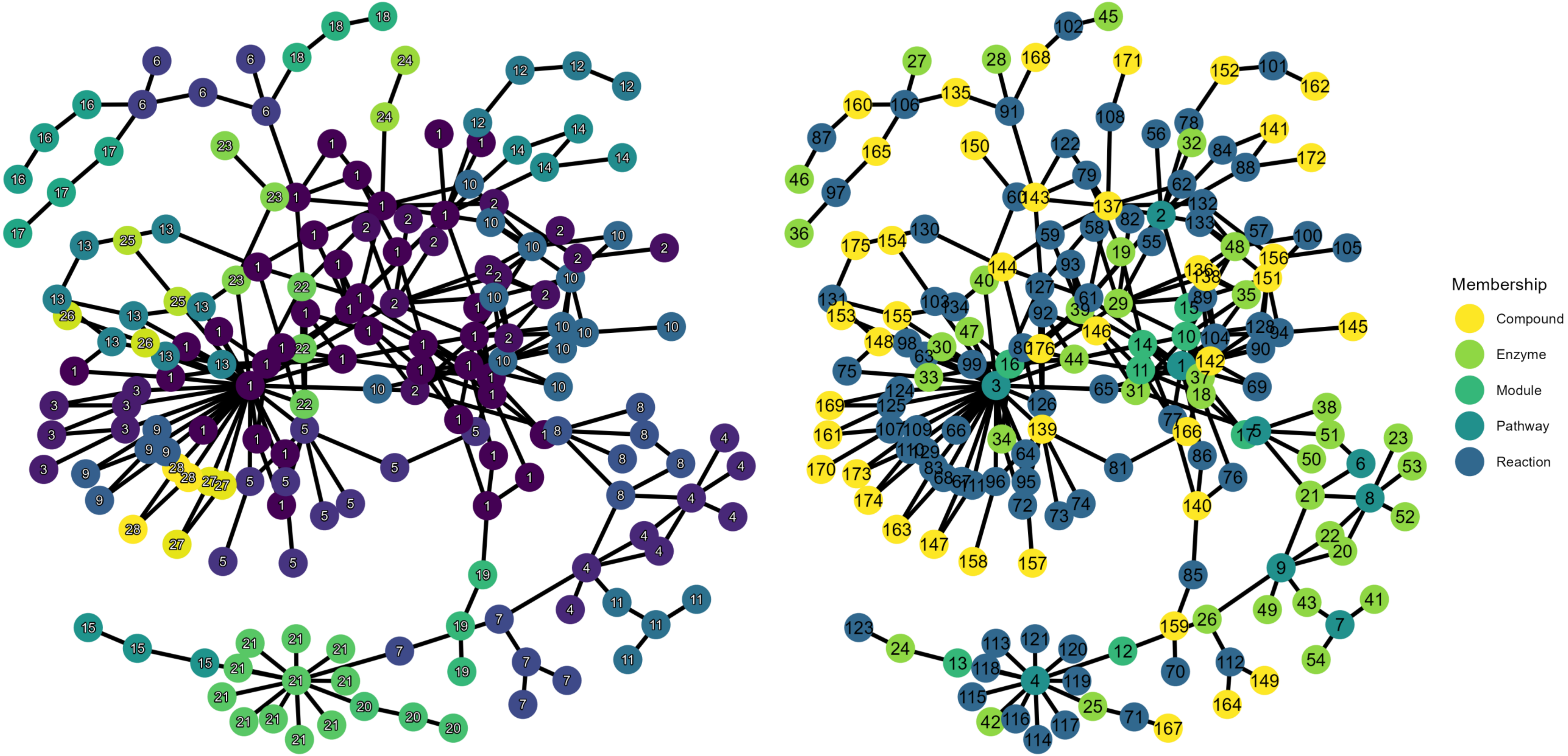

YMD4537_SLAD-2PE_vs_SHAD-2PE

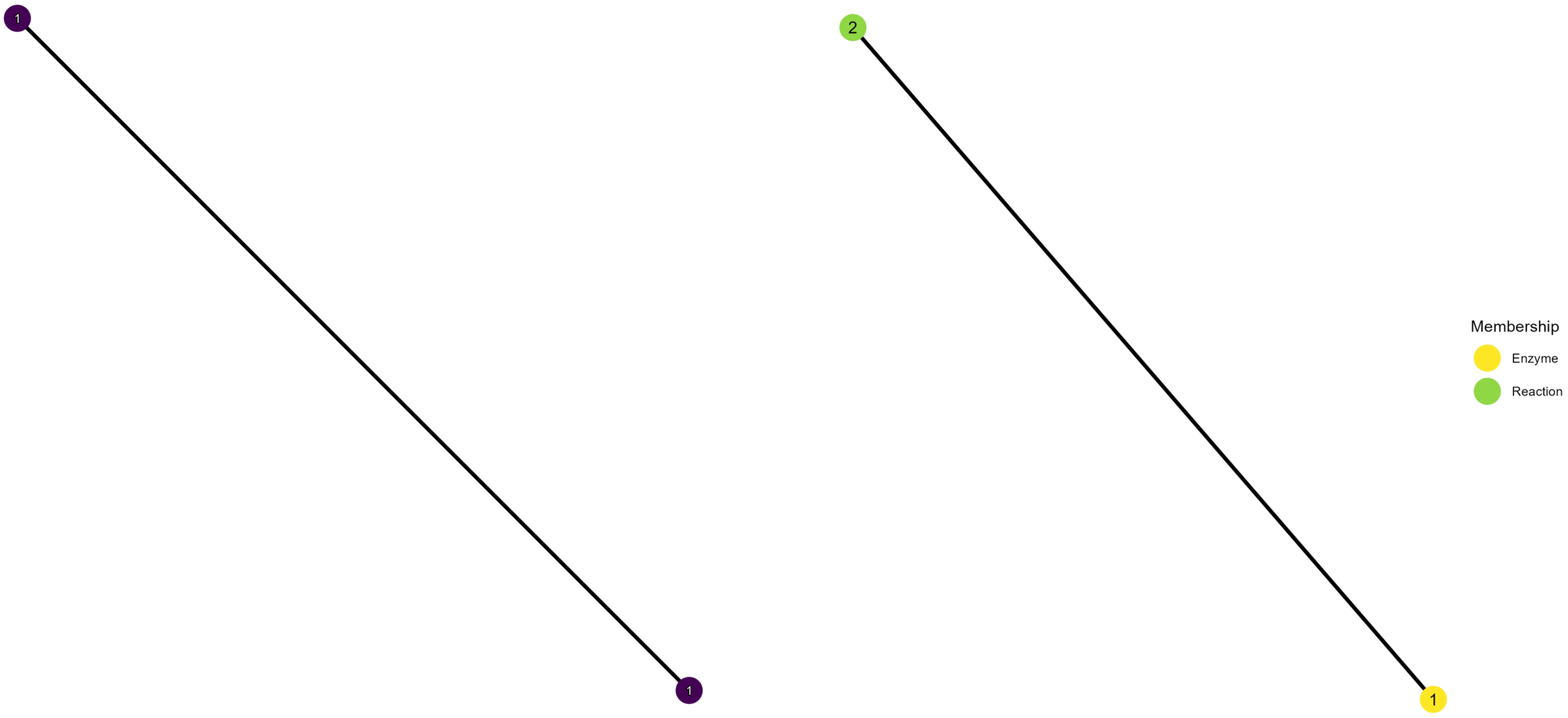

YMD4544_SLAD_vs_SHAD

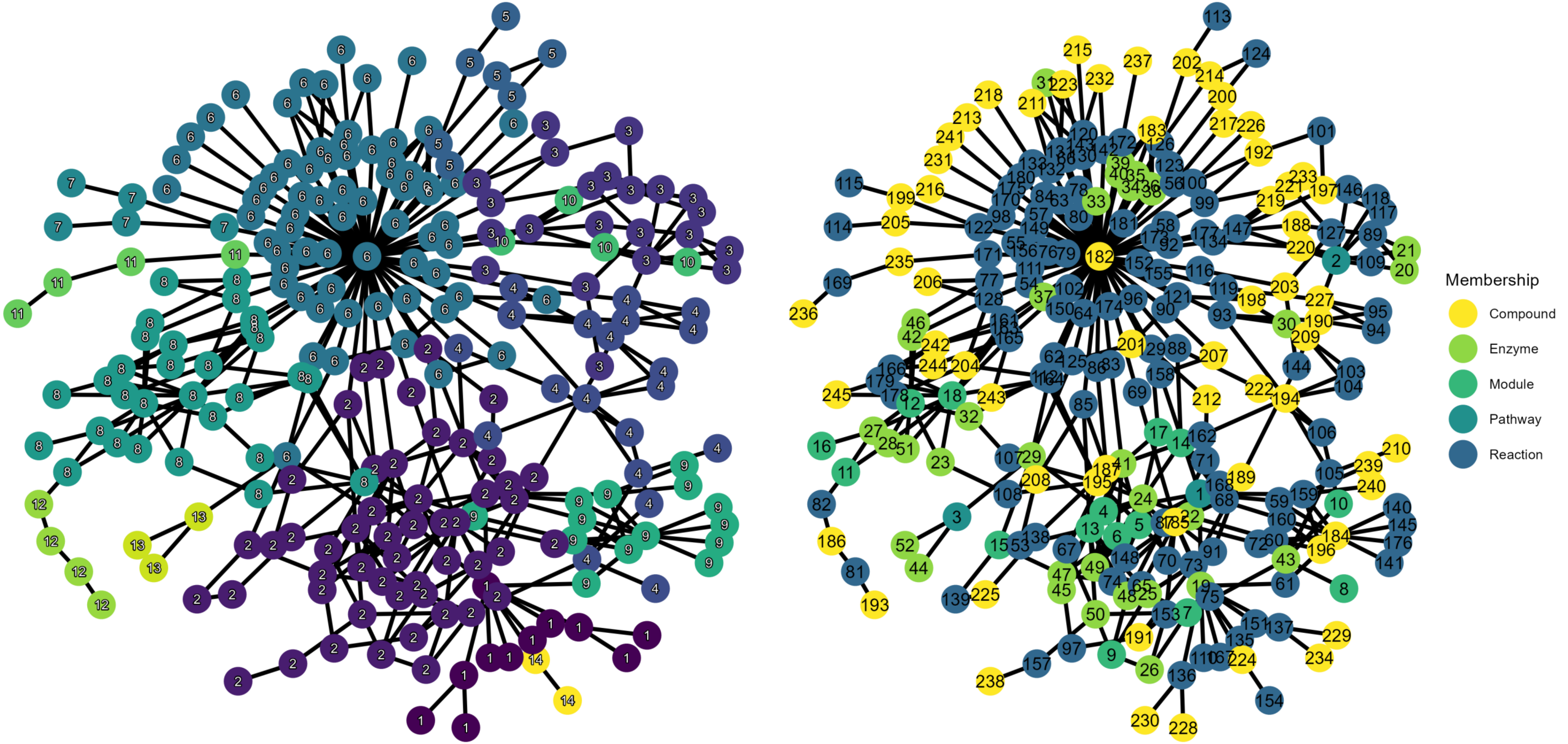

YMD4544_SHAD_vs_SHAD-2PE

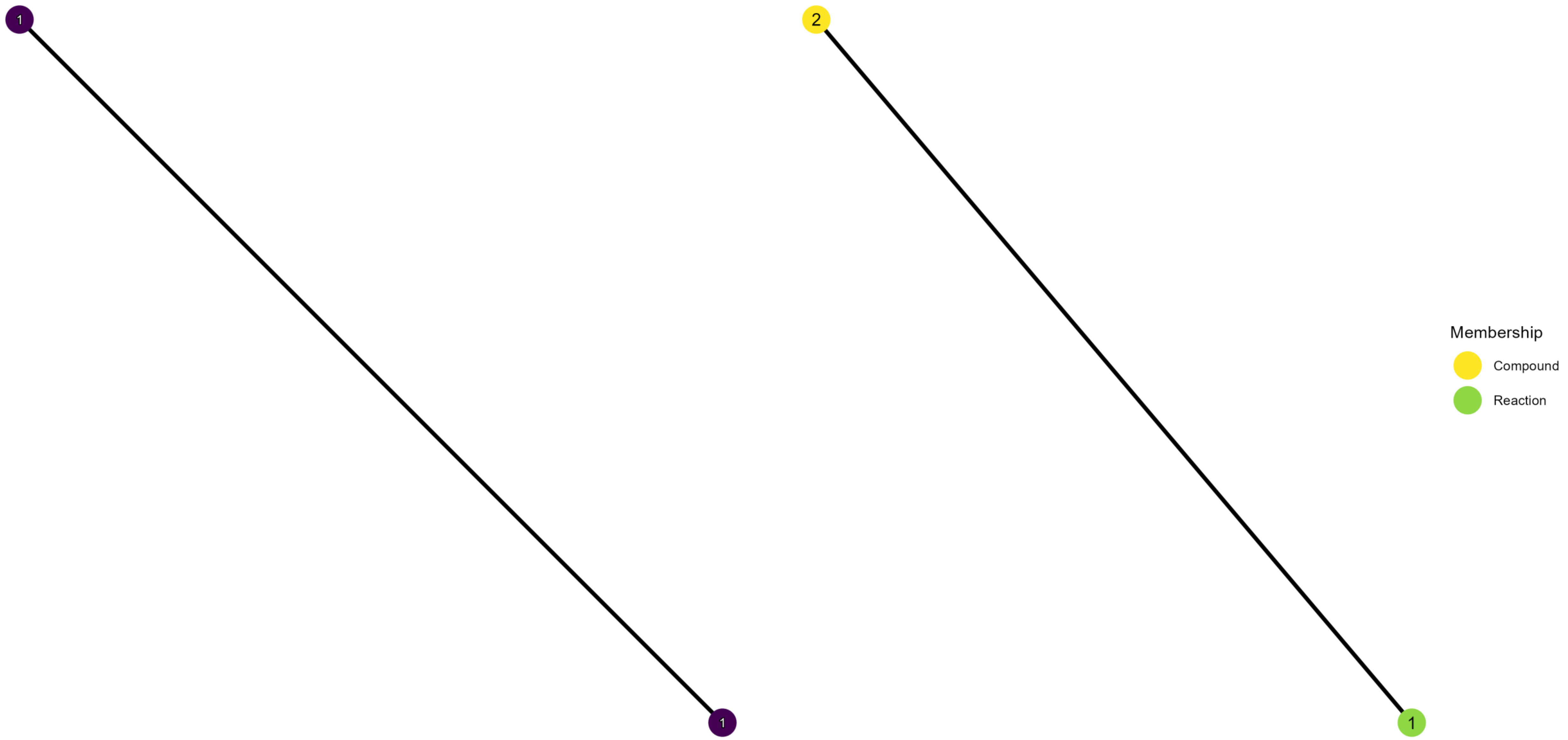

YMD4544_SLAD-2PE_vs_SHAD

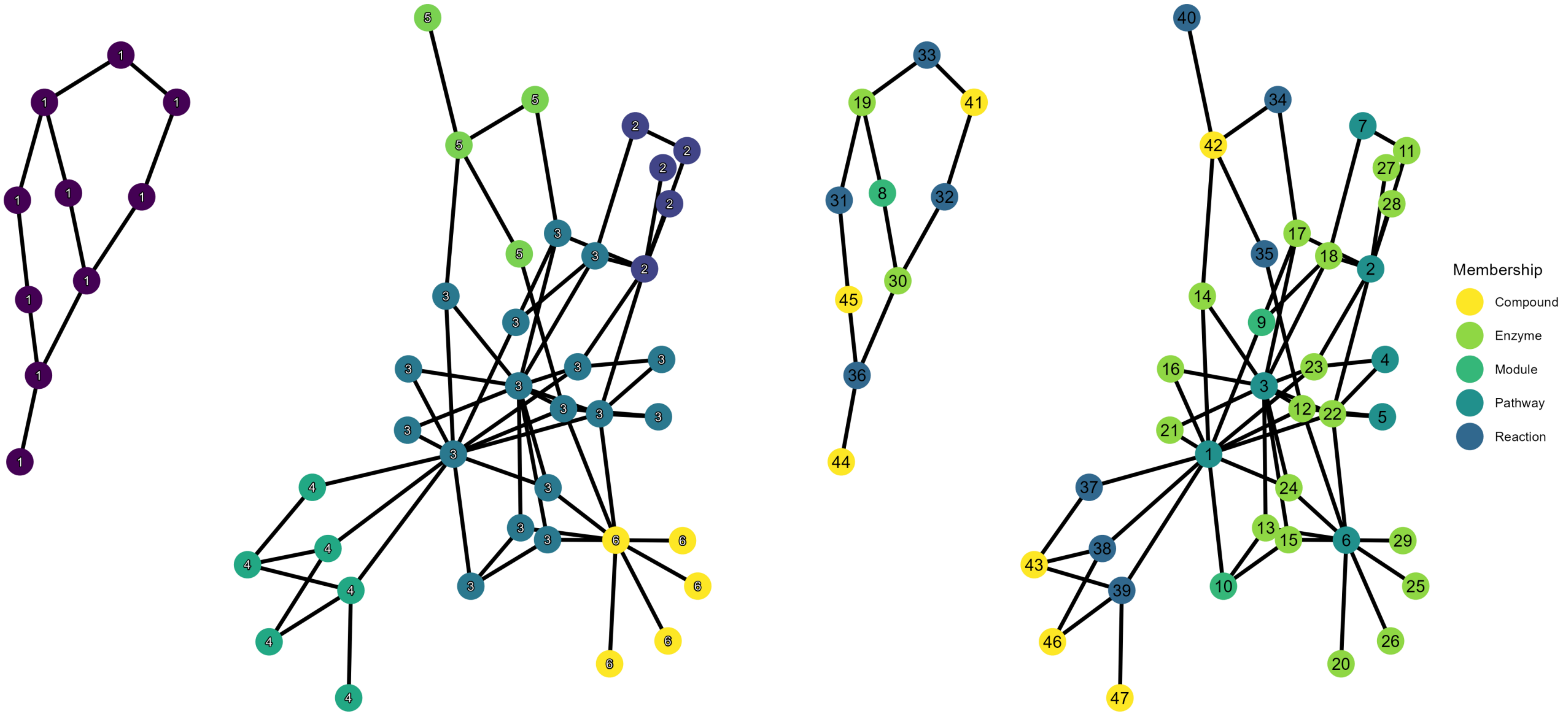

YMD4544_SLAD-2PE_vs_SHAD-2PE
