# Supplementary figures and images for: Quorum sensing in Saccharomyces cerevisiae brewing strains: effects of 2-phenylethanol on proteomic, lipidomic, and metabolomic profile
Source: FEMS Yeast Res. 2025 Jul 7;25:foaf036. doi: 10.1093/femsyr/foaf036 (PMC12254953; doi:10.1093/femsyr/foaf036)

# YMD4529 - Proteome Analysis

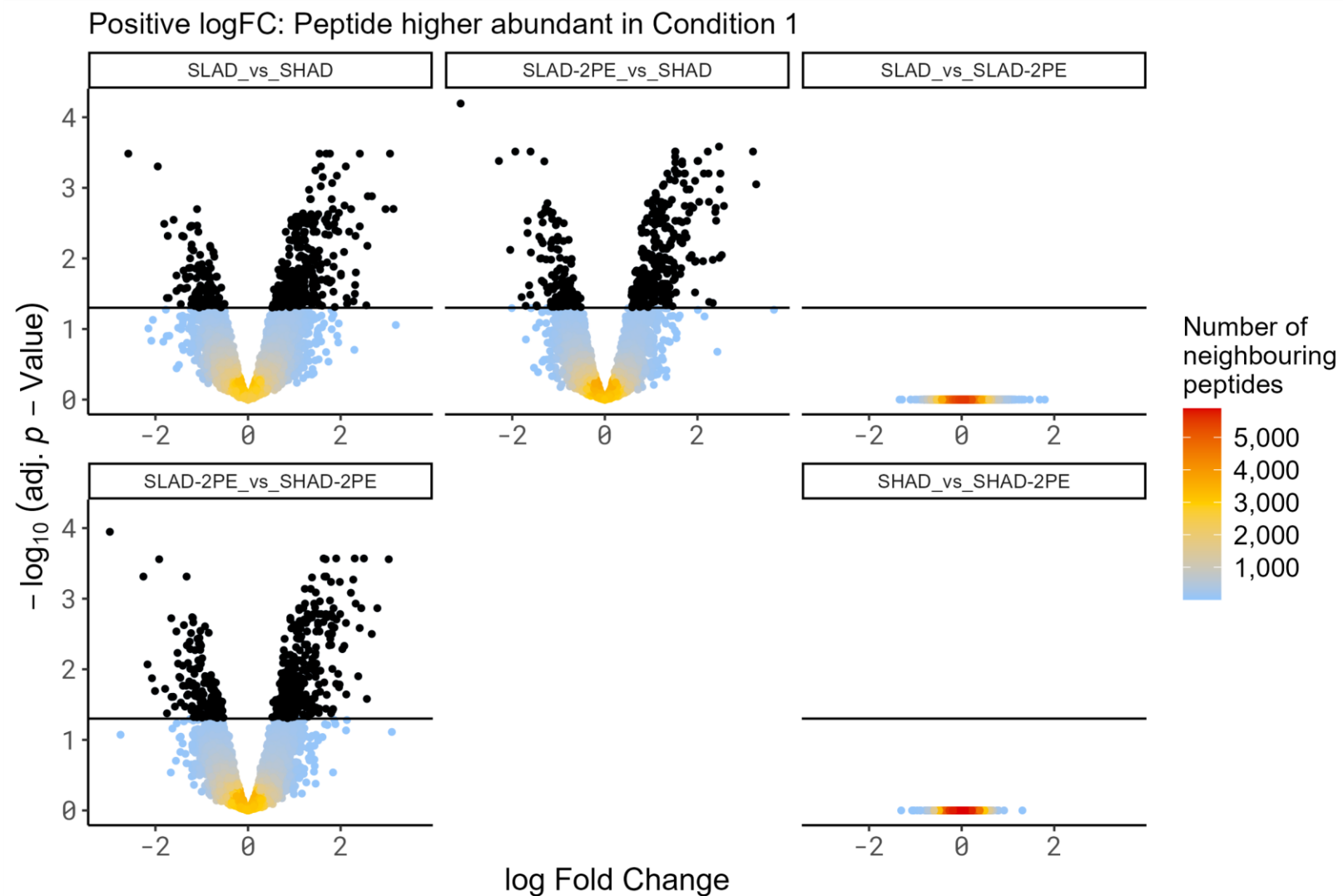

# YMD4537 - Proteome Analysis

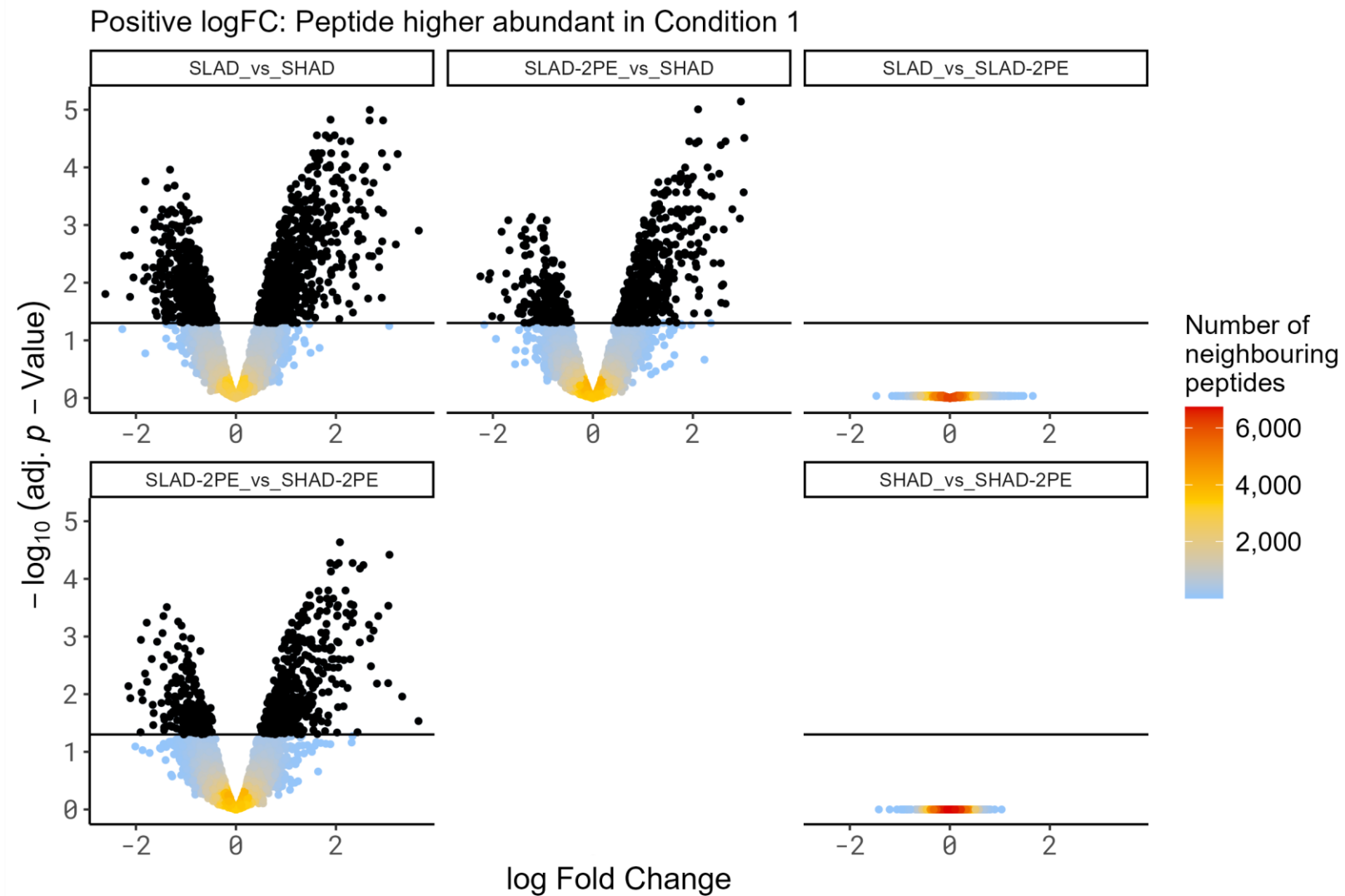

# YMD4544 - Proteome Analysis

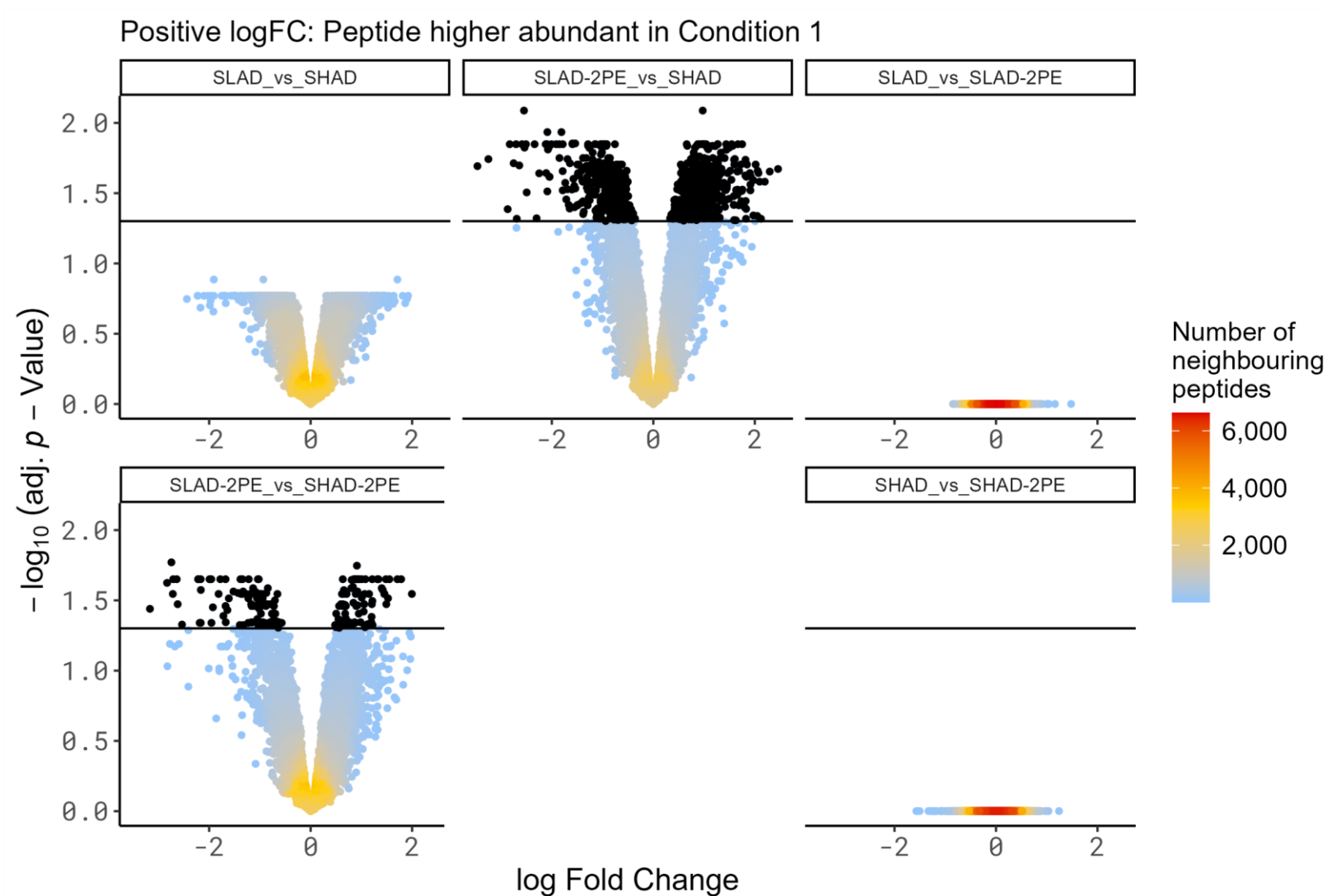

Supplement: foaf036_Supplemental_Files [file foaf036_supplemental_files.zip › Supplement_Figure_01.pdf]
